# Rare earth benzene tetraanion-bridged amidinate complexes†

**DOI:** 10.1039/d4sc05982d

**Published:** 2024-12-17

**Authors:** Peng-Bo Jin, Qian-Cheng Luo, Gemma K. Gransbury, Richard E. P. Winpenny, David P. Mills, Yan-Zhen Zheng

**Affiliations:** a Frontier Institute of Science and Technology, State Key Laboratory of Electrical Insulation and Power Equipment, MOE Key Laboratory for Nonequilibrium Synthesis of Condensed Matter, Xi'an Key Laboratory of Electronic Devices and Materials Chemistry and School of Chemistry, Xi'an Jiaotong University 99 Yanxiang Road Xi'an Shaanxi 710054 P. R. China zheng.yanzhen@xjtu.edu.cn; b Department of Chemistry, The University of Manchester Oxford Road Manchester M13 9PL UK richard.winpenny@manchester.ac.uk david.mills@manchester.ac.uk

## Abstract

The benzene tetraanion-bridged rare earth inverse arene amidinate complexes [{Ln(κ^1^:η^6^-Piso)}_2_(μ-η^6^:η^6^-C_6_H_6_)] (2-Ln, Ln = Gd, Tb, Dy, Y; Piso = {(NDipp)_2_C^*t*^Bu}, Dipp = C_6_H_3_^i^Pr_2_-2,6) were prepared by the reduction of parent Ln(iii) bis-amidinate halide precursors [Ln(Piso)_2_X] (Ln = Tb, Dy; X = Cl, I) or [Ln(Piso)_2_I] (Ln = Gd, Y) with 3 eq. KC_8_ in benzene, or by the reaction of the homoleptic Ln(ii) complexes [Ln(Piso)_2_] (Ln = Tb, Dy) with 2 eq. KC_8_ in benzene. The arene exchange reaction of 2-Tb with toluene gave crystals of [{Tb(κ^1^:η^6^-Piso)}_2_(μ-η^6^:η^6^-C_7_H_8_)] (3-Tb), while no reactions were observed when C_6_D_6_ solutions of 2-Y were separately treated with biphenyl, naphthalene or anthracene. The reactivity study shows that 2-Y can behave as a four-electron reductant to reduce 1,3,5,7-cyclooctatetraene (COT). Complexes 2-Ln were characterized by single crystal X-ray diffraction, elemental analysis, SQUID magnetometry, UV-vis-NIR, ATR-IR, NMR, density functional theory (DFT) and *ab initio* calculations. These data consistently show that 2-Ln formally contain Ln(iii) centres with arene-capped inverse-sandwich Dipp–Ln(iii)–(C_6_H_6_)^4−^–Ln(iii)–Dipp configurations, and DFT calculations on a model of 2-Y revealed strong Y–(C_6_H_6_)^4−^ δ-bonding interactions between the filled π-orbitals of the benzene tetraanion and vacant 4d orbitals of the Y(iii) ions. A strong intermolecular coupling interaction between the two Tb(iii) centres in 2-Tb (*J*_tot_ = −6.84 cm^−1^) was evidenced by a step in a magnetization *vs.* field plot of 2-Tb at *ca*. 3.4 T at 2 K, which we attribute to an anti-ferromagnetic transition of the magnetic moment; we also determined an exchange coupling constant *J*_ex_ = −0.25(1) cm^−1^ for 2-Gd.

## Abstract 2

Arene capped inverse-sandwich rare earth complexes stabilized by Ln–[C_6_H_6_]^4−^ covalent δ-bonding.

## Graphical abstract

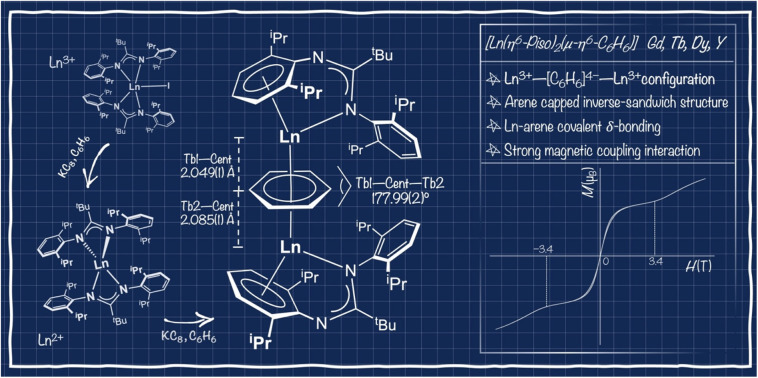

## Introduction

**Fig. 1:**
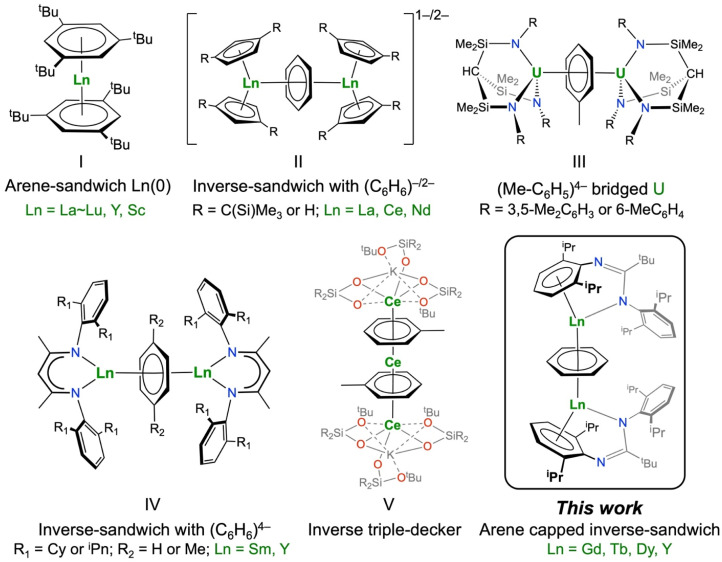
Selected examples of RE and An arene-coordinated sandwich (I), inverse-sandwich (II–IV), and inverse triple-decker (V) structures relevant to this work.

Sandwich compounds, which contain metals/metalloids bound by carbocyclic rings in a multihapto-fashion, have played a pivotal role in organometallic chemistry for nearly 75 years.^1,2^ Substituted cyclopentadienyl (C_5_R_5_, Cp^R^) and cyclooctatetraenyl (C_8_R_8_, COT^R^) ligands have been most extensively investigated for f-element sandwich complexes due to their negatively charged aromatic carbon rings, which are well-suited to the predominantly ionic bonding for these metals.^3–7^ Sandwich complexes of benzene and other substituted arene rings (C_6_R_6_) are well-developed for the d-transition metals,^8^ but because such ligands are typically neutral and aromatic with six π electrons, their rare earth (RE: group 3 and lanthanides; Ln) and actinide (An) chemistry is relatively immature.^9,10^ For example, classic bis(benzene)metal complexes were established for the d-block metals in the 1950s,^11^ but it took another 30 years for analogous RE complexes to be discovered, *e.g.* [M(C_6_H_3_^*t*^Bu_3_-1,3,5)_2_] (M = La–Lu, Y, Sc; I, Fig. 1).^12–15^ A U(iii) complex containing a single arene, [U(C_6_H_6_)(AlCl_4_)_3_], was first reported in 1971,^16^ whilst the permethylated analogues [M(C_6_Me_6_)(AlCl_4_)_3_] (M = U, Sm) were structurally authenticated in 1987.^17^

Extended RE and An sandwich complexes containing stacks of metal ions and aromatic rings have been synthesized; the simplest “inverse-sandwich” examples contain two f-block metals and one bridging carbocyclic ring.^18^ And the simplest “triple-decker” examples contain two f-block metals and three carbocyclic rings, with one ring bridging the two metals and two terminal rings.^19–24^ To date RE multiple-decker complexes have been exclusively constructed using bridging COT^R^ ligands as these large dianionic rings are well-suited for stabilizing Ln(iii) or Ln(ii) ions,^25–27^ including [Ln_2_^III^(COT′′)_3_] (Ln = Y, La, Ce, Nd, Sm, Tb, Dy, Ho, Er, Tm, Lu; COT′′ = C_8_H_6_(SiMe_3_)_2_-1,4),^21,22,28–31^ [Sm_3_^III/II/III^(COT^TIPS^)_4_] (COT^TIPS^ = C_8_H_6_(Si^i^Pr_3_)_2_-1,4)^20,32^ and [*cyclo*-Ln^II^(μ-η^8^:η^8^-COT^TIPS^)]_18_.^33^ f-Block complexes made up of three arene rings have remained elusive to date, though multiple-decker sandwich complexes with bridging arenes such as [V_2_(Cp)_2_(μ-C_6_H_6_)]^34^ and [Cr_2_(C_6_H_3_Me_3_-1,3,5)_2_(μ-C_6_H_3_Me_3_-1,3,5)]^35^ are well-known for the d-block metals.^36,37^

Over the last twenty-five years, many examples of RE and An complexes have been reported that formally contain reduced arene anions and variable metal oxidation states,^10,38–43^ accommodating a variety of arene formal charges including *e.g.* 7π-electron radical monoanions (C_6_H_6_^−^˙) in [K(18-crown-6)(η^2^-C_6_H_6_)_2_][{(Cp^tt^)_2_La^II^}_2_(μ-η^6^:η^6^-C_6_H_6_)] (Cp^tt^ = C_5_H_3_^*t*^Bu_2_-1,3);^18^ 8π e^−^ dianions (C_6_H_6_^2−^) in [{[(N^i^Pr)_2_CN(SiMe_3_)_2_]_2_Ln}(μ-η^6^:η^6^-C_6_H_6_)] (Ln = Y, Dy, Er),^44^ [K(18-crown-6)]_2_[{(Cp′′)_2_Ln^II^}_2_(μ-η^6^:η^6^-C_6_H_6_)] (Ln = La, Ce; Cp′′ = C_5_H_3_(SiMe_3_)_2_-1,3; II),^45–47^ and 10π e^−^ tetraanions (C_6_H_6_^4−^) in [{(Ts^R^)_2_U}_2_(μ-η^6^:η^6^-C_6_H_5_Me)] (Ts^R^ = Tos^Tol^, HC(SiMe_2_NAr′)_3_, Ar′ = C_6_H_4_Me-4; Tos^Xy^, HC(SiMe_2_NAr′′)_3_, Ar′′ = C_6_H_3_Me_2_-3,5; III),^48,49^ [{(BDI)Ln^III^(THF)_*n*_}_2_(μ-η^6^:η^6^-C_6_H_6_)] (Ln = Y, Sm; BDI = HC{C(Me)N[C_6_H_3_(^i^C_5_H_11_)_2_-2,6]_2_}; IV)^50–52^ and [{(C_5_^i^Pr_5_)Ln}(μ-η^6^:η^6^-C_6_H_6_)] (Ln = Y, Gd, Tb, Dy, Tm);^53^ C_6_H_6_^4−^ rings have also been reported for Th arene complexes.^41,54^ In most f-block complexes that contain reduced arenes these anions tend to be stabilized by two metal ions in a bridging motif, since the parent arene has a high reduction potential (*e.g.* benzene, *E*^*Θ*^ = −3.42 V *vs.* SHE)^55^ and metal ions with vacant valence orbitals provide stabilization by accepting electron density *via* δ-bonding.^44–54^ This metal-based stabilization is also seen in an inverse triple-decker complex [K(2.2.2-crypt)]_2_[{(KL_3_Ce)(μ-η^6^:η^6^-C_7_H_8_)}_2_Ce] (V), which formally contains two bridging toluene dianions sandwiching three Ce(ii) ions.^56^ There are a number of reports of RE and An complexes containing terminal arene anions,^57–62^ though mononuclear [K(18-crown-6)][(Cp′′)_2_Ln^III^(C_6_H_6_)] (Ln = La, Ce) have been shown to contain (C_6_H_6_)^2−^ dianions.^63^

**Fig. 2:**
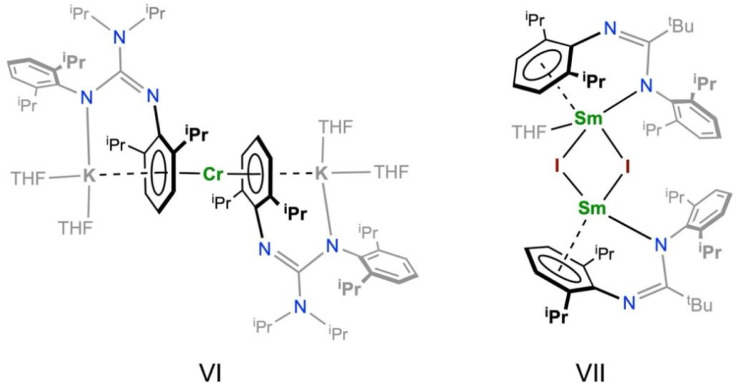
Sterically demanding guanidinate (VI) and amidinate (VII) metal complexes with η^6^-bound N-arene substituents.

It has previously been shown that neutral arene rings of bulky terphenyl ligands such as NHAr^iPr6^ (Ar^iPr6^ = C_6_H_3_(C_6_H_2_^i^Pr_3_-2,4,6)_2_-2,6) can coordinate to RE and An ions to make a bis(arene) sandwich motif with two pendant N-donor ligands for Ln(ii) (La = Sc, Y, La, Sm, Eu, Tm, Yb)^64,65^ and U(ii)^66^ ions. Similarly, amidinate/guanidinate ligands with arene N-substituents have also shown that they can form bis(arene) d-transition metal complexes, *e.g.* the Cr(0) complex [Cr(η^6^-Priso)_2_][K(THF)_2_]_2_ (VI; Priso = {(NDipp)_2_CN^i^Pr_2_}, Dipp = C_6_H_3_^i^Pr_2_-2,6), and f-block complexes with a κ^1^-N,η^6^-chelating binding motif, *e.g.* the Sm(ii) complex [Sm(κ^1^-N,η^6^-Piso)(THF)(μ-I)_2_Sm(κ^1^-N,η^6^-Piso)] (VII; Piso = {(NDipp)_2_C^*t*^Bu}) (Fig. 2).^67,68^ We therefore opted to use the bulky amidinate Piso^−^ as a supporting ligand in this work.^69,70^

Here we present the synthesis and characterization of a series of structurally analogous Ln inverse-sandwich arene amidinate complexes, [{Ln(κ^1^:η^6^-Piso)}_2_(μ-η^6^:η^6^-C_6_H_6_)] (2-Ln, Ln = Gd, Tb, Dy, Y) and [{Tb(κ^1^:η^6^-Piso)}_2_(μ-η^6^:η^6^-C_7_H_8_)] (3-Tb). These complexes show arene-capped inverse structures with two Ln(iii) ions sandwiched in between three arene rings, with a bridging arene tetraanion and two terminal Piso ligands binding in a κ^1^:η^6^-C_6_ fashion. Magnetic studies of 2-Tb and 2-Gd revealed that the C_6_H_6_^4−^ bridge promotes a large intermolecular interaction between the two Ln(iii) centres, with steps at ∼±3.4 T observed in magnetization curves in 2-Tb and an exchange coupling constant *J*_ex_ = −0.25(1) cm^−1^ determined in 2-Gd, while a weak antiferromagnetic interaction is seen between the two Dy(iii) centres in 2-Dy.

## Results and discussion

### Synthesis

**Scheme 1:**
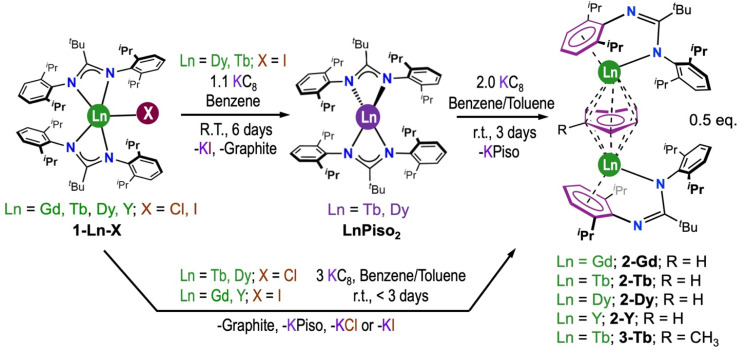
Synthesis of 2-Ln from [Ln(Piso)_2_Cl] (1-Ln-Cl, Ln = Tb, Dy) and [Ln(Piso)_2_I] (1-Ln-I, Ln = Gd, Tb, Dy, Y).

The heteroleptic Ln(iii) bis-amidinate halide complexes [Ln(Piso)_2_Cl] (1-Ln-Cl, Ln = Tb, Dy), [Ln(Piso)_2_I] (1-Ln-I, Ln = Gd, Tb, Dy, Y) were prepared by the respective salt metathesis reactions of parent LnCl_3_ ^71^ or LnI_3_ ^72^ with two equivalents of KPiso^73^ in toluene at reflux, by modifying literature protocols (Scheme 1).^74^1-Ln-I (Ln = Tb, Dy) were reported previously.^74^ The benzene-bridged arene-capped inverse-sandwich Ln(iii) amidinate complexes 2-Ln were synthesized by the reduction of 1-Ln-X with 3 eq. KC_8_ in benzene; dark blue or purple crystals of 2-Ln were isolated in *ca*. 40% yields following work-up and recrystallization from dark purple (2-Gd, 2-Tb and 2-Dy) or dark blue (2-Y) pentane solutions at −35 °C (see Experimental section: synthesis for a more detailed description). As shown in our previous study, mononuclear divalent complexes [Ln(Piso)_2_] (Ln = Tb, Dy) were obtained if [Ln(Piso)_2_I] (Ln = Tb, Dy) are reduced with 1.1 eq. KC_8_ in benzene.^75^ During the synthesis of 2-Tb and 2-Dy, the colourless [Ln(Piso)_2_I] precursors first converted to dark green [Ln(Piso)_2_], and then to dark purple 2-Ln; these complexes can be alternatively prepared by the stoichiometric reaction of [Ln(Piso)_2_] with KC_8_ in benzene (Scheme 1). These reactions therefore formally proceed by the transfer of two electrons from the two Ln(ii) centres and two electrons from two K(0) to form 1 eq. of 2-Ln, with concomitant elimination of 2 eq. KPiso. By contrast, when 1-Y-I was treated with 1.1. eq. KC_8_ in benzene only 2-Y and 1-Y-I could be assigned in the reaction mixture by NMR spectroscopy, and the putative dark green Y(ii) complex [Y(Piso)_2_] was not detected by X-band EPR spectroscopy, which only revealed a sharp feature with *g* = 2.0048 (Fig. S81†); Y(ii) complexes tend to show *g* values slightly lower than 2.00.^76^ We posit that once [Y(Piso)_2_] is formed it reacts rapidly with KC_8_ to give 2-Y, as Y(ii) is more reactive than Tb(ii) and Dy(ii) according to the standard reduction potential of the Y(iii)/(ii) couple (−2.66 V; −3.06 V) being lower than those of Tb(iii)/(ii) (−2.55 V; −2.95 V) and Dy(iii)/Dy(ii) (−2.56 V; −2.96 V) (The former *vs.* SHE and the latter *vs.* Fc^+^/Fc).^77^ The differences in reduction potentials could be even larger for Y(iii)/(ii) and Ln(iii)/(ii) (Tb, Dy) ions in the [Ln(Piso)_2_] coordination environment. The exchange reaction of 2-Tb in neat toluene at room temperature was evidenced by the isolation of crystals of 3-Tb after 24 h. We determined from colour changes of reaction mixtures that 3-Tb and its diamagnetic analogue 3-Y could also be prepared by the respective reactions of parent 1-Ln-X with excess KC_8_ in toluene, analogously to the synthesis of 2-Ln. Elemental analysis and ATR-IR spectroscopy was performed on microcrystalline samples of 1-Ln-X, 2-Ln and 3-Tb, and further characterization data were obtained as appropriate. As we only isolated a small amount of 3-Tb we limited further characterization of this complex to single crystal XRD and UV-vis-NIR spectroscopy only (see below).

### Structural characterization

**Fig. 3:**
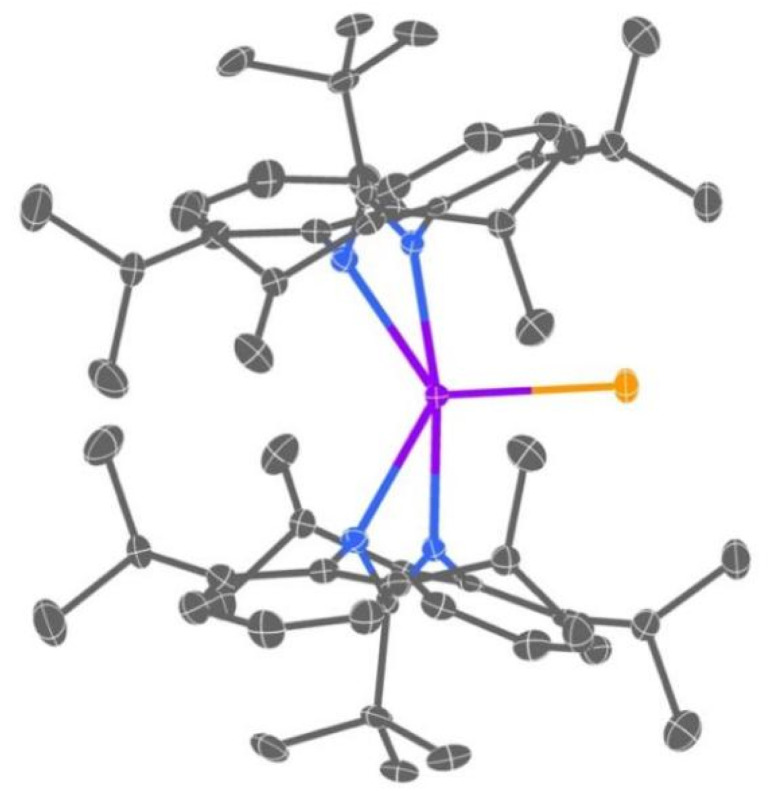
Molecular structure of 1-Tb-Cl with selective atom labelling (C = gray, N = blue, Cl = yellow, Tb = violet). Thermal ellipsoids set at 50% probability level and hydrogen atoms omitted for clarity.

**Fig. 4:**
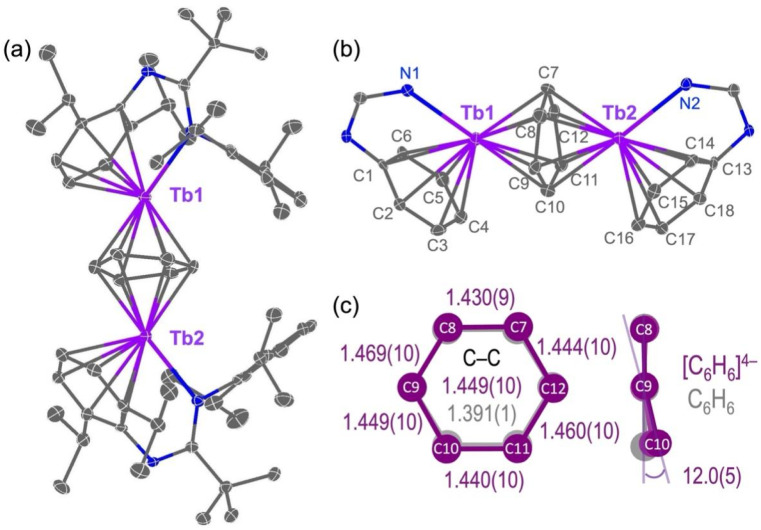
(a) Molecular structure of 2-Tb; (b) central core of 2-Tb with selected atom labelling; and, (c) comparison of structural parameters between (C_6_H_6_)^4−^ and free benzene, which are extracted from the crystallographic data of 2-Tb and Chemcraft software, respectively.^115^ The displacement ellipsoids are set at the 50% probability level, and hydrogen atoms and lattice solvent are omitted for clarity. Key: Tb(iii) = violet, N = blue, C = gray.

The solid-state structures of 1-Ln-X, 2-Ln and 3-Tb were determined by single crystal X-ray diffraction (see Fig. 3 for a depiction of 2-Tb-Cl, Fig. 4a and b for 2-Tb and Fig. S56–S62† for other structures). Crystals of [Ln(Piso)_2_Cl] (Ln = Tb, Dy) are solvent-free, whereas 1-Ln-I (Ln = Gd, Y), 2-Ln and 3-Tb contain stoichiometric toluene, hexane or pentane in their crystal lattices, respectively. Complexes 1-Ln-X are five-coordinate, with the Ln centres bound by one chloride or iodide and four N-donor atoms from the two chelating κ^2^-*N*,*N*′-Piso ligands (Fig. 3 and S56–S58†), in common with the previously reported structures of [Ce(Piso)_2_Cl] and [Ln(Piso)_2_I] (Ln = Tb, Dy).^74,75^ The coordination geometry is intermediate between trigonal bipyramidal and square pyramidal (*τ*_5_ = 0.85 for Gd, 0.84 for Tb, 0.88 for Dy and 0.86 for Y).^78^ Complexes 2-Ln and 3-Tb feature a triple-decker structure with a μ-η^6^:η^6^-arene forming the middle deck and two terminal η^6^-bound Dipp substituents of the Piso ligands, whereby the Piso ligands bind to the two Ln ions in a κ^1^-N,η^6^-fashion to complete the Ln coordination spheres.

The structural parameters for 2-Ln and 3-Tb are summarized in Table 1. The Ln–N distances of 2-Ln (Gd: 2.476(2)–2.488(2) Å; Tb: 2.446(5)–2.480(5) Å; Dy: 2.418(7)–2.438(7) Å and Y: 2.430(2)–2.442(2) Å) are longer than those of 1-Ln-X (Gd: 2.333(2)–2.444(2) Å; Tb: 2.333(3)–2.402(2) Å; Dy: 2.332(3)–2.388(3) Å; Y: 2.296(2)–2.397(2) Å), due to the change in Piso binding mode. By contrast with [Ln_2_(η^8^:η^8^-COT)_2_(μ,η^8^:η^8^-COT)],^28–31^ the central cores of 2-Ln and 3-Tb feature bent geometries, with C_cent1_–Ln1–C_cent2_ angles of 137.16(2)° (2-Gd), 135.34(2)° (2-Tb), 134.45(3)° (2-Dy) and 134.17(2)° (2-Y) and Ln1–C_cent2_–Ln2 angles of 177.50(2)° (2-Gd), 177.99(2)° (2-Tb), 178.20(2)° (2-Dy) and 177.42(1)° (2-Y), due to the steric requirements of the κ^1^-N,η^6^-bound Piso ligand. The μ-η^6^:η^6^-bridging arenes in these complexes bind to the two Ln ions asymmetrically with Ln–C_arene_ distances of 2.446(3)–2.630(3) Å (2-Gd), 2.452(6)–2.632(7) Å (2-Tb), 2.419(9)–2.590(9) Å (2-Dy), 2.403(2)–2.604(2) Å (2-Y), 2.421(3)–2.615(2) Å (3-Tb), and with mean Ln–C_cent2_ distances of 2.070(1) Å (2-Gd), 2.067(1) Å (2-Tb), 2.039(1) Å (2-Dy), 2.032(1) Å (2-Y) and 2.054(1) Å (3-Tb) (Fig. S59–S62† and Table 1). These are ∼0.4 Å shorter than the respective mean distances between the Ln centres and the terminal C_6_-rings, due to the greater electrostatic attraction of tetraanionic (C_6_H_6_)^4−^*vs.* neutral Dipp rings. The carbocyclic C–C bond lengths of the bridging arenes in 2-Ln and 3-Tb are 1.430(9)–1.471(13) Å and 1.455(5)–1.466(4) Å, respectively, which are longer than the terminal Dipp rings of 1.378(16)–1.441(12) Å and the aromatic C–C distances of free benzene (1.391(1) Å)^79^ (Fig. 4c). The bridging arenes in 2-Ln are not planar, *e.g.* in 2-Tb showing a dihedral angle of 12° between the planes defined by C9–C10 and C9, C8, C7, C12 (Fig. 4c), consistent with the arene ring being reduced. We cannot confidently assign (R–C_6_H_5_)^2−^ or (R–C_6_H_5_)^4−^ ring charges from structural data alone, as both rings can exhibit structural distortions from planarity as shown previously for other inverse-sandwich f-block arene complexes,^45–53^ and (R–C_6_H_6_)^4−^ rings can be planar because of 10π-electron aromatic system.^54^ Therefore, we used a combination of methods to probe whether Ln(ii)–C_6_H_6_^2−^–Ln(ii) or Ln(iii)–C_6_H_6_^4−^–Ln(iii) formulations best-describe the electron structures of 2-Ln (see below).

**Table 1:** Selected bond distances (Å) and angles (°) for 2-Ln (Ln = Gd, Tb, Dy, Y) and 3-Tb

Complex	2-Gd^d^	2-Tb	2-Dy	2-Y	3-Tb
Ln1–N1	2.484(4)/2.488(2)	2.480(5)	2.446(7)	2.442(2)	2.469(2)
Ln2–N2	2.476(2)/2.488(2)	2.446(5)	2.424(7)	2.430(2)	2.463(3)
Ln1–C^a^	2.793(3)–2.864(3)	2.766(7)–2.863(7)	2.744(9)–2.811(9)	2.744(2)–2.818(3)	2.793(4)–2.852(3)
	Avg. 2.833(3)	Avg. 2.817(6)	Avg. 2.768(9)	Avg. 2.775(3)	Avg. 2.819(3)
Ln1–C^b^	2.446(3)–2.630(3)	2.452(6)–2.604(7)	2.419(9)–2.590(9)	2.426(3)–2.604(2)	2.437(3)–2.615(2)
	Avg. 2.532(3)	Avg. 2.516(7)	Avg. 2.501(9)	Avg. 2.498(2)	Avg. 2.519(3)
Ln2–C^b^	2.450(3)–2.627(3)	2.456(6)–2.632(7)	2.422(9)–2.573(9)	2.403(2)–2.589(2)	2.421(3)–2.602(3)
	Avg. 2.532(3)	Avg. 2.527(7)	Avg. 2.507(9)	Avg. 2.501(2)	Avg. 2.516(3)
Ln2–C^c^	2.812(3)–2.876(3)	2.795(7)–2.887(7)	2.727(9)–2.840(9)	2.739(2)–2.865(2)	2.798(3)–2.858(2)
	Avg. 2.844(3)	Avg. 2.830(6)	Avg. 2.770(9)	Avg. 2.793(3)	Avg. 2.826(3)
Ln1–C_cent1_^a^	2.489(1)/2.461(1)	2.438(1)	2.392(1)	2.389(1)	2.436(1)
Ln1–C_cent2_^b^	2.062(1)/2.067(1)	2.049(1)	2.035(1)	2.029(1)	2.054(1)
Ln2–C_cent2_^b^	2.083(1)/2.070(1)	2.085(1)	2.042(1)	2.035(1)	2.055(1)
Ln2–C_cent3_^c^	2.472(1)/2.474(1)	2.466(1)	2.405 (1)	2.420(1)	2.450(1)
C––C(ring)^a^	1.387(5)–1.389(5)	1.380(9)–1.425(9)	1.382(14)–1.430(13)	1.391(4)–1.426(3)	1.393(5)–1.432(4)
	Avg. 1.404(5)	Avg. 1.404(9)	Avg. 1.408(13)	Avg. 1.408(3)	Avg. 1.406(4)
C––C(ring)^b^	1.440(5)–1.469(5)	1.430(9)–1.469(10)	1.444(13)–1.471(13)	1.450(3)–1.468(3)	1.455(5)–1.466(4)
	Avg. 1.453(5)	Avg. 1.449(10)	Avg. 1.463(14)	Avg. 1.461(3)	Avg. 1.460(5)
C––C(ring)^c^	1.389(5)–1.433(4)	1.400(10)–1.434(9)	1.369(14)–1.429(12)	1.394(14)–1.428(11)	1.393(4)–1.428(5)
	Avg. 1.404(5)	Avg. 1.411(10)	Avg. 1.401(13)	Avg. 1.409(4)	Avg. 1.408(4)
Ln1–C_cent2_–Ln2	177.50(2)/179.00(2)	177.99(2)	178.18(2)	177.42(1)	175.07(1)

a The C atoms and C_centroid1_ belong to the Dipp group bound to Ln1.

b The C atoms and C_centroid2_ belong to the bridging arene anion.

c The C atoms and C_centroid3_ belong to the Dipp group bound to Ln_2_.

d Two independent molecules of 2-Gd in the asymmetric unit.

### Solution characterisation and reactivity studies

Solutions of 1-Ln-X and 2-Ln in C_6_D_6_ were characterised by NMR spectroscopy. The ^1^H and ^13^C{^1^H} NMR spectra of 1-Y-I and 2-Y in C_6_D_6_ all show resonances that are typical for diamagnetic Y(iii) complexes, supporting the assignment of *S* = 0 singlet ground states of the {Dipp–Y(iii)–(C_6_H_6_)^4−^–Y(iii)–Dipp} motif, with the expected arene resonances presenting ^1^*J*_YC_ coupling constants (^89^Y, *I* = ½, 100% abundant) while no ^2^*J*_YH_ coupling observed. In 1-Y-I, the ^13^C{^1^H} NMR spectrum features a doublet resonance at *δ*_C_ = 182.69 ppm (^2^*J*_YC_ = 2 Hz) due to the quaternary CN_2_ fragment of Piso showing a chelating κ^2^-N,N′-binding mode to the Y(iii) centre (Fig. S6†). By contrast, for 2-Y a singlet peak was observed in the ^13^C{^1^H} NMR spectrum at 172.29 ppm as a κ^1^-N,η^6^-binding mode is adopted. Due to the strong interaction between the Y(iii) centres and C_6_H_6_^4−^, triplet resonances of bound C_6_H_6_^4−^ in 2-Y were seen at *δ*_C_ = 65.51 ppm (^1^*J*_YC_ = 5.1 Hz), but a singlet resonances at *δ*_H_ = 2.05 ppm without ^2^*J*_YH_ coupling observed, which are shifted considerably from free benzene singlet resonances seen at *δ*_C_ = 127.69 ppm and *δ*_H_ = 7.15 ppm, respectively (Fig. S11 and S12†). The chemical shift is primarily influenced by the shielding effect, which is closely related to the charge density around the hydrogen atoms. The charge distribution calculations reveal electron-rich C_6_H_6_^4−^ rings compared to the arene rings of terminal Dipp groups (Tables S30 and S31†), increasing shielding and leading to an upfield chemical shift. Similar triplet resonances were previously observed in the ^1^H and ^13^C{^1^H} NMR spectrum of complex IV for the Y(iii)-bound (C_6_H_6_)^4−^ moiety at *δ*_C_ = 65.74 ppm (^1^*J*_YC_ = 4.8 Hz) and *δ*_H_ = 2.37 ppm (^1^*J*_YH_ = 1.6 Hz),^50^ though we note that there is a large disparity in both the chemical shift and coupling constant for the ^1^H NMR resonances. Four additional arene resonances were observed in the ^13^C{^1^H} NMR spectrum of 2-Y for the *ipso*-, *ortho*-, *meta*- and *para*-positions of the Dipp groups that are bound to the Y(iii) centres, and these resonances are shifted by *ca*. 20 ppm downfield of the respective four peaks of the unbound Dipp groups. The *meta*-positions of the terminally bound Dipp groups also present a doublet of triplets (dt) resonance in the ^1^H NMR spectrum of 2-Y at *δ*_H_ = 6.99 ppm (^2^*J*_HH_ = 7.7 Hz, ^1^*J*_YH_ = 4.2 Hz) for the Fig. S11.† We were unable to interpret the ^1^H and ^13^C{^1^H} NMR spectra of 1-Dy-Cl, 1-Tb-Cl, and 2-Dy due to paramagnetic broadening, whilst the ^1^H NMR spectrum of 2-Tb could be tentatively assigned by relative integrals, though these signals were also paramagnetically broadened and shifted to various extents between −90 and 290 ppm (Fig. S7†). The bridging (C_6_H_6_)^4−^ ring gives a sharp singlet peak at *δ*_H_ = 63.67 ppm, indicating that in solution dynamic processes lead to (C_6_H_6_)^4−^ not retaining the asymmetry seen in the solid state structure, as otherwise there would be several small peaks of varying intensity.

Arene exchange reactivity studies were performed on C_6_D_6_ solutions of 2-Y, and these reaction mixtures were monitored by ^1^H and ^13^C{^1^H} NMR spectroscopy (Fig. S15–S19†); such exchange processes have previously been reported for a wide range of inverse-arene RE and An complexes.^10,38,39,61^ However, arene exchange did not proceed when separate 1 : 1 mixtures of 2-Y and biphenyl, naphthalene or anthracene were heated to 80 °C for over 100 hours, demonstrating the kinetic stability of 2-Y in C_6_D_6_ (Fig. S16–S18†). Similar results for polyaromatic hydrocarbons (PACs) such as naphthalene, anthracene and biphenyl have previously been reported for other Ln and An complexes with tetraanionic (C_6_H_6_)^4−^ bridging units.^50,52,54^ To extend this study and to account for the formation of 3-Tb, we monitored the exchange reaction of a C_7_D_8_ solution of 2-Y in an NMR tube at 80 °C for over 100 hours (Fig. S19†). No peaks corresponding to 3-Y were observed, with the NMR spectra appearing to show 2-Y and some minor decomposition products, indicating that arene exchange did not proceed under these conditions. A similar situation is also found for a solution of 2-Tb in C_7_D_8_ in an NMR tube, where no obvious arene exchange occurred at 80 °C for over 100 hours (Fig. S22†). However, a sample of 2-Y dissolved in toluene at room temperature and stirred for 12 h showed that arene exchange proceeds readily under these conditions to give 3-Y, analogously to the formation of 3-Tb. Volatiles were removed *in vacuo* and the reaction mixture was monitored by ^1^H and ^13^C{^1^H} NMR spectroscopy to reveal signals at *δ*_C_ = 123.04 ppm and *δ*_H_ = 5.2 ppm that may correspond to the Y(iii)-bound (C_6_H_5_CH_3_)^4−^ moiety of 3-Y (Fig. S13 and S14†). However, the arene exchange did not proceed to completion, with traces of the (C_6_H_6_)^4−^ moiety of 2-Y seen at *δ*_C_ = 65.58 ppm and *δ*_H_ = 2.05 ppm, with a 2-Y : 3-Y ratio of 37 : 63. This indicates that using large amounts of toluene with stirring could increase the rate of the exchange reaction and allow it to proceed at a lower temperature. Similar stability differences between benzene anions and alkyl-substituted aromatic rings have been reported in previous reactivity studies of f-block inverse-sandwich complexes.^50,80,81^

The separate reactions of 2-Y with COT under molar ratios of 1 : 2 and 1 : 4 were performed in C_6_D_6_. The reaction mixtures changed colour from dark blue to pale yellow within 5 min, indicating that 2-Y has been fully consumed. Both reactions were monitored by ^1^H NMR spectroscopy, giving relatively clean spectra (Fig. S20†). No traces of (C_6_H_6_)^4−^ remained at 2.05 ppm but a new signal for (C_8_H_8_)^2−^ was seen at 6.59 ppm, showing that C_8_H_8_ had been reduced and (C_6_H_6_)^4−^ had been oxidised. For the 1 : 4 reaction of 2-Y with COT, about 53% free COT was also detected at 5.63 ppm, which lowers to 5% in the 1 : 2 reaction. This indicates that 2-Y acts as a four electron reductant in terms of reducing COT. This is similar to the recently reported (C_6_H_6_)^4−^ bridged Sm_2_ complexes [{(BDI)Sm(THF)_*n*_}_2_(μ-η^6^:η^6^-C_6_H_6_)], in which 1 equiv. (C_6_H_6_)^4−^ can also reduce 2 equiv. COT to yield a mononuclear product [(BDI)Sm(COT)].^50^

We also investigated the reactivity of 2-Y with THF, by treating 2-Y (12 mg, 0.01 mmol) with a drop of THF-d_8_ in C_6_D_6_ (0.6 mL) or neat THF-d_8_ (0.6 mL). The colour of the former solution lowered in intensity but remained blue, while the latter reaction mixture became colourless after several minutes. These two reactions were monitored by ^1^H NMR spectroscopy (Fig. S21†), where in the former a weak trace of triplet signal of C_6_H_6_^4−^ remained, whilst no signal for C_6_H_6_^4−^ was seen for the neat THF-d_8_ solution. This shows that 2-Y is not stable in THF as it reacts with this ethereal solvent, *via* oxidation of C_6_H_6_^4−^.

### UV-vis-NIR spectroscopy

**Fig. 5:**
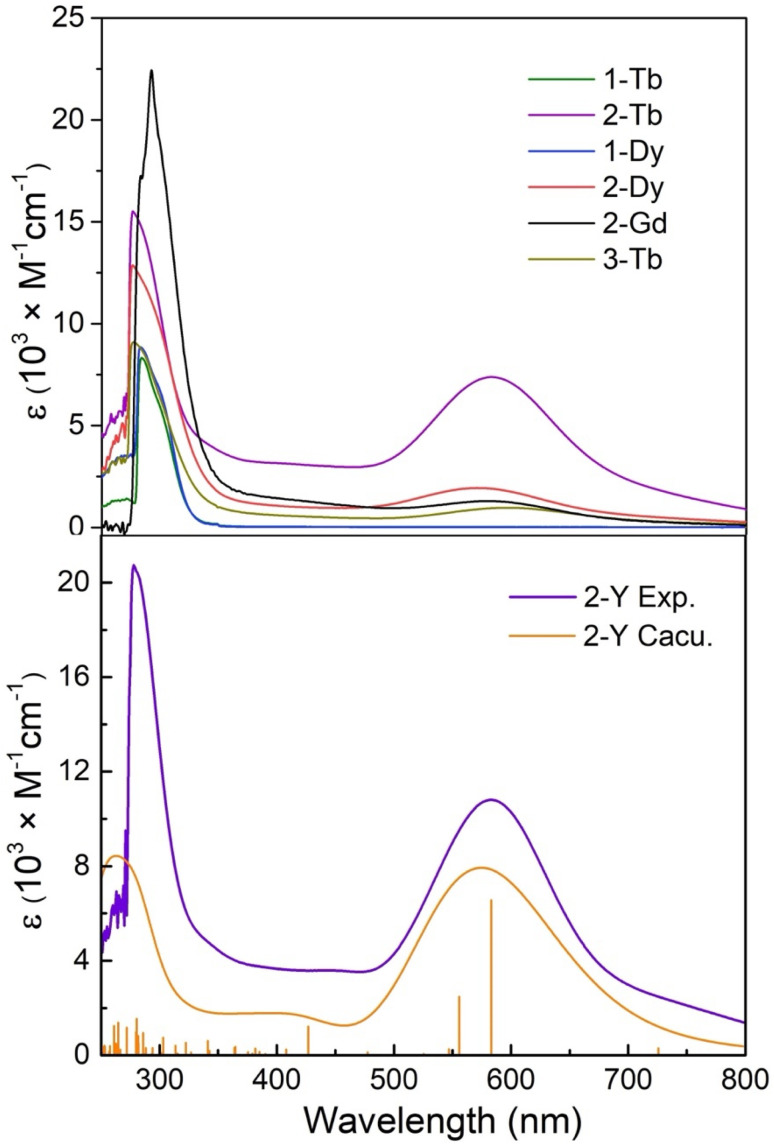
Experimental UV-vis-NIR spectra of solutions of (above) 1-Gd-I, 2-Gd, 1-Tb-Cl, 2-Tb, 1-Dy-Cl, 2-Dy and 3-Tb in benzene at room temperature; (bottom) experimental (purple) and calculated (orange) UV-vis spectra of 2-Y in benzene at room temperature, with pertinent theoretical excitations shown as vertical lines. The full width at half maximum (FWHM) in the simulated UV-vis spectrum is set at 0.5 eV.

The UV-vis-NIR absorption spectra of separate hexane or benzene solutions of 1-Ln-X, 2-Ln and 3-Tb were determined from 250–800 nm (12 500–40 000 cm^−1^) (Fig. 5 and S33–S55†). Strong and sharp absorptions are found below 300 nm (*ε* > 6000 M^−1^ cm^−1^) for all complexes. Similar absorptions have been observed for other Piso^−^ complexes, as well as both HPiso and KPiso, and these are therefore assigned to intraligand π to π* transitions (Fig. 5).^74^ The Ln(iii) complexes 1-Ln-X appear colourless and their electronic absorption spectra are essentially featureless in the visible and NIR region, with no Laporte-forbidden f–f transitions observed for 1-Gd-I, 1-Tb-Cl or 1-Dy-Cl (Fig. S32–S34†).^3^ Hexane or benzene solutions of the arene-bridged complexes 2-Ln and 3-Tb are dark green to dark blue, and show broad absorption bands stretching across most of the visible region from 500–700 nm; the intensities of these transitions likely vary with solvent due to differences in dielectric constants and intermolecular interactions. Amongst this series of complexes, 2-Y gives the largest extinction coefficients, with maxima in benzene at *ṽ*_max_ ∼17 240 cm^−1^/∼580 nm (*ε* = 11 500 M^−1^ cm^−1^) (Fig. S49–S51†); similar features are seen for 2-Gd (*ṽ*_max_ 17 120 cm^−1^; *ε* = 1296 M^−1^ cm^−1^ in benzene), 2-Tb (*ṽ*_max_ 17 240 cm^−1^; *ε* = 8350 M^−1^ cm^−1^ in benzene and *ε* = 5015 M^−1^ cm^−1^ in hexane) (Fig. S36–S48†), 2-Dy (*ṽ*_max_ 17 540 cm^−1^/570 nm; *ε* = 2180 M^−1^ cm^−1^) (Fig. S43–S48†) and 3-Tb (*ṽ*_max_ 16 670 cm^−1^/600 nm; *ε* = 970 M^−1^ cm^−1^) (Fig. S52†). These absorptions are mainly attributed to the charge transfer transitions between the bridging arenes and the phenyl of terminal Piso ligand, which differ from the broad bands covering the visible region in [{(BDI)M(THF)_*n*_}_2_(μ-η^6^:η^6^-arene)] (M = Y, Sm) based on C_6_H_6_^2−^ and R–C_6_H_5_^4−^,^50^ and are bathochromically shifted compared to *ṽ*_max_ ≈ 540 nm in [{(Cp′′)_2_La}_2_(μ-η^6^:η^6^-C_6_H_6_)]^2−^.^45^ The spectra of 2-Ln are comparable to those of parent Ln(ii) complexes [Ln(Piso)_2_] (Ln = Tb, Dy), but the peak maxima shift to higher energy from *ca.* 700 nm to 580 nm, and the extinction coefficients of 2-Tb are much higher than seen for [Tb(Piso)_2_] (2100 M^−1^ cm^−1^). Similar to [Ln(Piso)_2_], the high thermostability of 2-Ln was demonstrated by heating benzene solutions of 2-Ln 80 °C for 12 hours; the intense colours were maintained and the UV-vis-NIR spectra show the same absorption features as fresh solutions, but with lower extinction coefficients (Fig. S53–S55†).^75^

### Magnetism

**Fig. 6:**
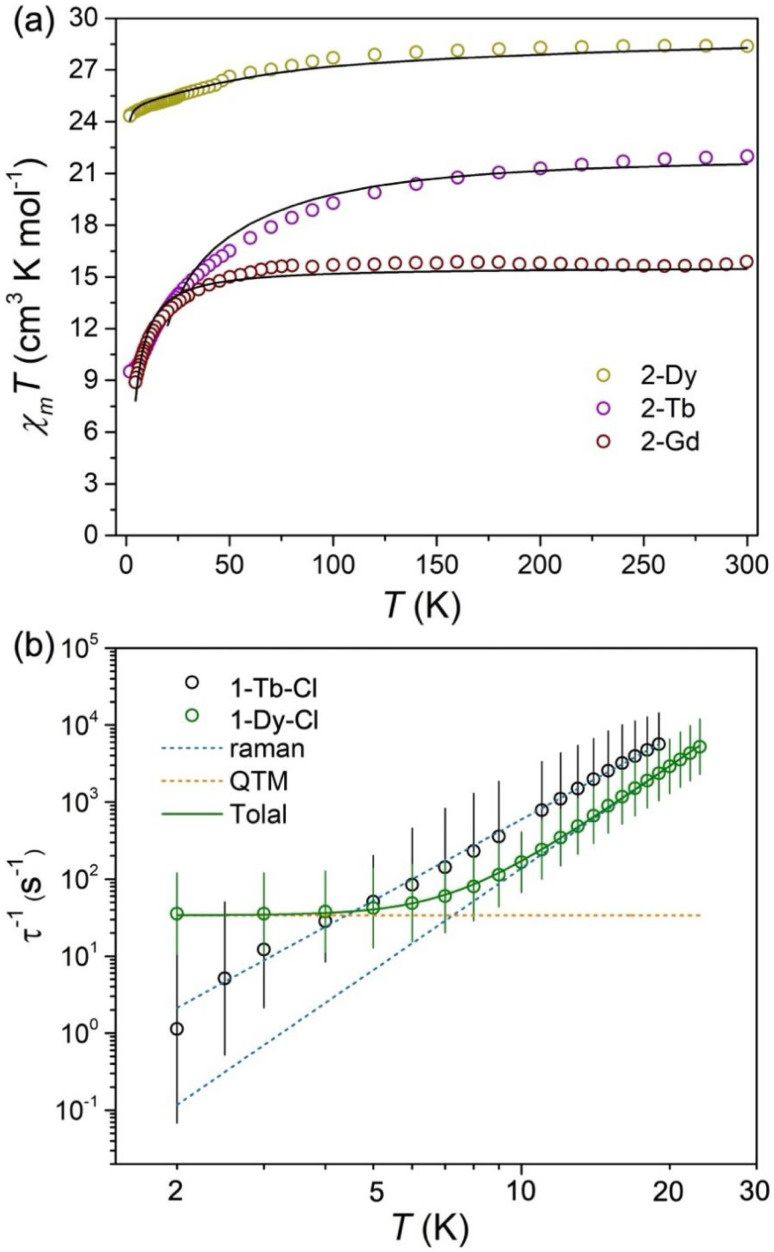
(a) Temperature dependent susceptibility of *χ*_M_*T* in an applied dc magnetic field of 1 kOe for 2-Dy, 2-Tb and 2-Gd. The black solid lines represent the best fit from *ab initio* calculations (2-Dy and 2-Tb) or eqn (1) (2-Gd). (b) Plot of natural log of the inverse relaxation time *vs.* temperature for 1-Tb-Cl and 1-Dy-Cl. The plots are from the ac susceptibility measurements with applied field (0 Oe for 1-Dy-Cl and 2 kOe for 2-Tb-Cl). The lines are the best fit.

To better understand the electronic structures and arene anion exchange coupling between the Ln centres of 2-Ln, static and dynamic magnetic susceptibility data were collected for microcrystalline samples of 1-Tb-Cl, 1-Dy-Cl, 2-Gd, 2-Tb, 2-Dy and 2-Y suspended in eicosane (Fig. 6 and S63–S80†). The magnetic susceptibility temperature product (*χ*_M_*T*) was determined for 1-Ln-Cl and 2-Ln between 1.8 and 300 K under an applied dc field of 1000 Oe. The *χ*_M_*T* products at 300 K of 1-Tb-Cl, 1-Dy-Cl, 2-Tb and 2-Dy are 12.27, 13.80, 21.98 and 28.39 cm^3^ mol^−1^ K, respectively. The values for 1-Ln-Cl are comparable to the corresponding expected free-ion *χ*_M_*T* values for 4f^8^ Tb(iii) (11.81 cm^3^ mol^−1^ K) and 4f^9^ Dy(iii) (14.17 cm^3^ mol^−1^ K), and that of 2-Dy essentially matches the theoretical value for two Dy(iii) ions (28.34 cm^3^ mol^−1^ K), whilst the value for 2-Tb is slightly below that predicted for two Tb(iii) ions (23.62 cm^3^ mol^−1^ K). The free-ion *χ*_M_*T* values of Tb(ii) and Dy(ii) under both 4f^*n*+1^ (4f^9^, Tb(ii), 14.17 cm^3^ mol^−1^ K; 4f^10^, Dy(ii), 14.06 cm^3^ mol^−1^ K) and 4f^*n*^5d^1^ (4f^8^5d^1^, Tb(ii), 12.19–14.43 cm^3^ mol^−1^ K; 4f^9^5d^1^, Dy(ii), 14.55–17.02 cm^3^ mol^−1^ K) valence electronic structures are not consistent with those measured for 2-Ln.^75,82,83^ Additionally, the solution magnetic susceptibilities of 2-Tb and 2-Dy were determined at 298 K by the Evans method;^84^ the values obtained (*χ*_M_*T* = 23.24 and 28.24 cm^3^ mol^−1^ K) are in line with the solid-state magnetic data and that expected for two uncoupled Tb(iii) and Dy(iii) ions (*χ*_M_*T* = 23.62 and 28.34 cm^3^ mol^−1^ K).

For 2-Gd, the *χ*_M_*T* product at 300 K is 15.89 cm^3^ K mol^−1^, which is excellent agreement with the theoretical value of 15.76 cm^3^ K mol^−1^ for two uncoupled Gd(iii) ions (^8^S_7/2_, *g* = 2). When the temperature is decreased, the *χ*_M_*T* value keeps almost constant until around 75 K, and then starts to decrease more rapidly which is mainly attributed to intramolecular antiferromagnetic (AFM) coupling (Fig. 6a). The fitting of *χ*_M_^−1^*versus T* above 50 K based on the Curie–Weiss law gives a negative Weiss constant *θ* = −1.21 K and a Curie constant *C* = 15.83 cm^3^ K mol^−1^. Furthermore, the analytical Van Vleck equation based on the equivalent operator approach and the spin Hamiltonian of *Ĥ* = −*JŜ*_Gd1_*Ŝ*_Gd2_ was used to reproduce the dc magnetic susceptibility data (eqn (1)). The best fit gives the parameter of *J* = −0.25(1) cm^−1^ when the Landé factor *g* is fixed as 2.00, which is smaller but comparable to the values *J* = −0.642(6) cm^−1^ and −2.94(2) cm^−1^ for other biphenyl or benzene inverse-sandwich Gd complexes {[(NN^TBS^)Gd]_2_(μ-biphenyl)}^−^ (NN^TBS^ = fc(NSi^*t*^BuMe_2_)_2_, fc = 1,1′-ferrocenediyl),^53,85^ especially the latter has the shortest Gd–arene distances 2.039(6) Å observed to date. These values are much larger than those reported previously for Gd_2_ systems with non-radical bridges,^86^ indicating that the tetraanionic arene^4−^ bridge is capable of transmitting strong magnetic exchange. In addition, measurements on 2-Y at 300 K and 150 K and under 1000 Oe or 10 000 Oe showed it was diamagnetic. These data indicate that 2-Ln feature Ln(iii)–(C_6_H_6_^4−^)–Ln(iii) rather than Ln(ii)–(C_6_H_6_^2−^)–Ln(ii) configurations.

The *χ*_M_*T* values of 2-Dy decrease gradually with temperature from 300 K to 2 K, where a large *χ*_M_*T* value of 24.33 cm^3^ mol^−1^ K persists, while the *χ*_M_*T* values of 2-Tb decrease more markedly to reach 9.50 cm^3^ mol^−1^ K at 2 K. These data indicate that there is a stronger antiferromagnetic (AFM) interaction in 2-Tb than 2-Dy (Fig. 6a, S65 and S66†). For 1-Dy-Cl, the *χ*_M_*T* value decreases steadily with temperature to *ca*. 4 K followed by a sharp drop-off to 6.60 cm^3^ mol^−1^ K at 2 K, likely because of slow magnetic dynamics (Fig. S63†). By contrast, 1-Tb-Cl exhibits a smooth downturn in *χ*_M_*T* below *ca*. 20 K (Fig. S64†). Together, the static magnetic data for 1-Tb-Cl and 2-Tb indicate that magnetic dynamics are faster than the timescale of the experiment, and that 1-Dy-Cl exhibits slower magnetization dynamics than 1-Tb-Cl and 2-Ln. The zero-field cooled (ZFC) and field-cooled (FC) magnetic susceptibility traces support this assertion, showing bifurcation at 4 K for 1-Dy-Cl (Fig. S67†), while bifurcations are not found for 1-Tb-Cl and 2-Ln.

The magnetic relaxation rates of 1-Ln-Cl and 2-Ln (Ln = Tb, Dy) were probed by ac magnetic susceptibility experiments. Amongst these complexes, 1-Dy-Cl and 1-Tb-Cl exhibit single-molecule magnet (SMM) behaviour. For 1-Dy-Cl, slow magnetic relaxation with peaks in 
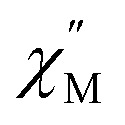
 were observed under zero dc field between 2 and 23 K (Fig. S69†), while for 1-Tb-Cl the non-Kramers Tb(iii) ion has non-zero splitting between the lowest-lying pseudo-doublet states (|+*m*_J_> and |−*m*_J_>), so 
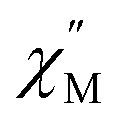
 peaks were observed between 2 and 19 K under an optimal dc field of 2000 Oe (Fig. S68†). No ac signals were seen for 2-Tb and 2-Dy due to the large tunnelling splitting (Δ_tun_) with strong QTM for exchange-coupled ground states (Tables S26 and S27†).^87^ The ac data of 1-Ln-Cl were fitted with a generalized Debye model^88^ in CC-FIT2 ^89,90^ to give the temperature dependence of magnetic relaxation times, which can be described with the general equations *τ*^−1^ = *CT*^*n*^ + *τ*_QTM_^−1^ and *τ*^−1^ = *CT*^*n*^ and fitted using CC-FIT2 to give *C* = 10^−2.253±0.042^ s^−1^ K^−*n*^; *n* = 4.394 ± 0.034; *τ*_QTM_ = 10^−1.531±0.014^ s for 1-Dy-Cl, and *C* = 10^−0.727±0.054^ s^−1^ K^−*n*^ and *n* = 3.502 ± 0.051 for 1-Tb-Cl (Fig. 5b, S72 and S73†). In these equations, two terms are combined: the through-barrier two-phonon Raman mechanism (phenomenological parameters *C* and *n*), and *τ*_QTM_, the QTM timescale. However, the over-barrier Orbach mechanism *τ*^−1^ = *τ*_0_^−1^ exp[−*U*_eff_/*T*] (where *U*_eff_ is the effective barrier to magnetic reversal and *τ*_0_ is the attempt time of the phonon bath) is not included due to an order of magnitude faster Raman process in 1-Tb-Cl compared to its' respective iodide analogues [Tb(Piso)_2_I].^75^ The Ln–X distances in [Ln(Piso)_2_I] are much longer than those in 1-Ln-Cl, leading to weaker Ln–X interactions, a smaller transverse field, slower magnetic relaxation and higher *U*_eff_ = 491 ± 32 and 800 ± 16 K for [Tb(Piso)_2_I] and [Dy(Piso)_2_I], respectively.^75^

Variable temperature magnetization *vs.* dc field measurements of 1-Ln-Cl and 2-Ln were performed with field sweep rates of 22 Oe s^−1^; all traces showed butterfly-shaped hysteresis loops at 2 K, confirming efficient QTM at zero field (Fig. S74–S80†). As expected, by 2 K the hysteresis loops for the non-Kramers Tb(iii) complexes 1-Tb-Cl are closed in the zero field region and this loop is only slightly open at higher fields at this temperature (Fig. S78†). For 1-Dy-Cl the hysteresis loops are completely closed at 4 K (Fig. S79†), which is consistent with the ZFC-FC bifurcation temperature and substantially lower than [Dy(Piso)_2_I], which shows loops that are opened at higher fields up to 10 K.^75^ The hysteresis loops of 2-Tb and 2-Dy are nearly closed at all fields by 2 K (Fig. S80†). A step-like loop is found in 2-Tb at 2 K as the applied magnetic field sweeps between ±5 T (Fig. S80† bottom), while the magnetization of 2-Dy is saturated above ∼1.5 T (10.5 μ_B_ at 5 T) without a step-shape (Fig. S80† above). The same 2-Tb step was observed in magnetization curves from 0 to 7 T at 2 K, but this is not present at 4 K and 7 K due to thermal excitation (Fig. S76 and S77†). The magnetization of 2-Tb doesn't appear to be saturated even at 7 T, with the steps in magnetization for 2-Tb located around ±3.4 T through calculation of its first derivative; this was attributed to the AFM interactions introduced by the arene-bridge.

### Electronic structure calculations

**Fig. 7:**
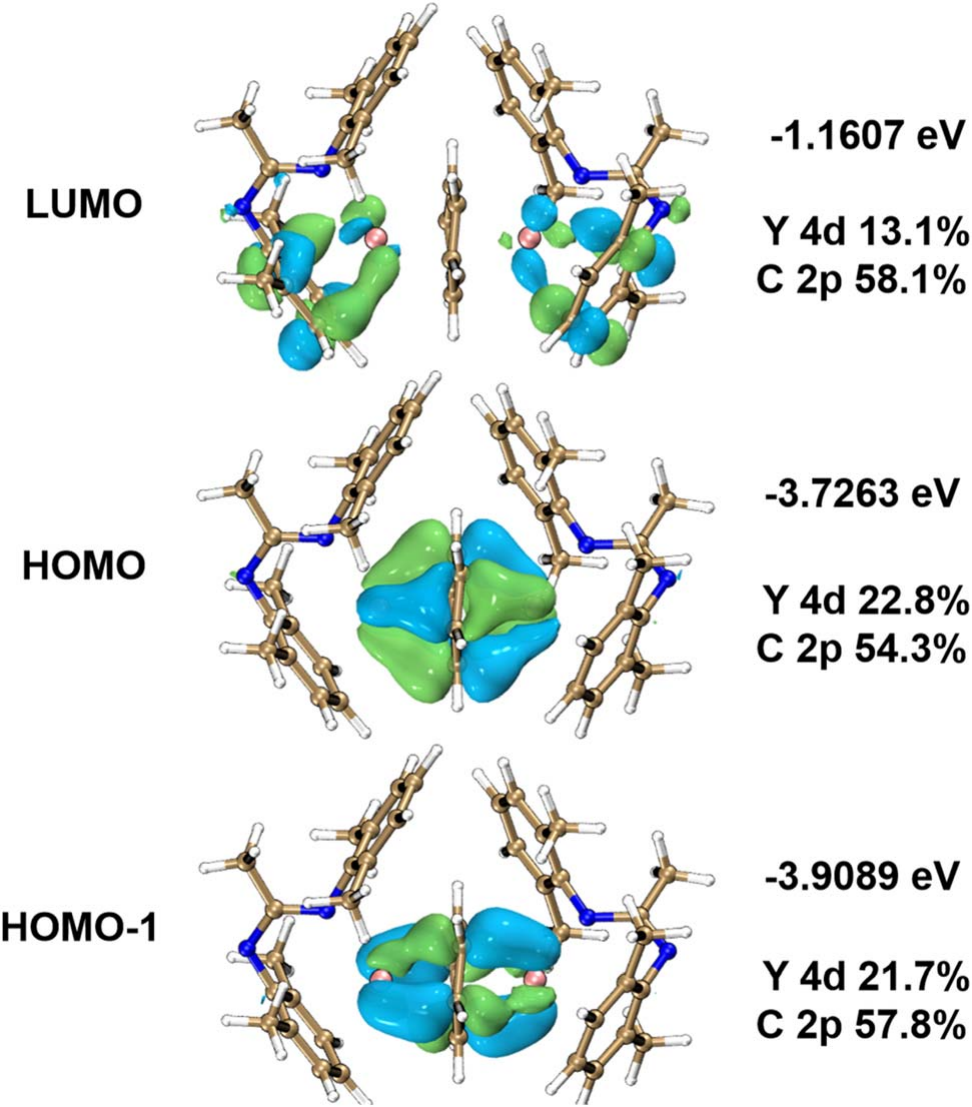
DFT-calculated frontier molecular orbitals (FMOs) for 2-Y^opt^ (isovalue = 0.04 a.u.). Orbital energies and corresponding compositions with Mulliken partitions are given on the right. Colour codes: Y, pink; N, blue; C, tan; H, white.

To gain further insights into the bonding features of 2-Ln and to verify the electronic states of the arene bridges, we performed density functional theory (DFT) calculations on a model complex of 2-Y using Orca 5.0,^91^ whereby the ^*t*^Bu and ^i^Pr groups on the Piso ligands were replaced to methyl groups for simplification. The closed-shell electron configuration was considered in the geometry optimization, and the corresponding optimized structure (2-Y^opt^) gives similar structural parameters compared with the single crystal XRD data for 2-Y (Fig. S82†); minor differences of bond lengths and angles were attributed to changes in steric and electronic effects from replacement of R-groups. For example, the Y⋯Y distance and mean Y–N and Y–C_cent2_ bond lengths calculated for 2-Y^opt^ are 4.00, 2.40 and 2.00 Å, which are slightly shorter than the experimental values for 2-Y of 4.06(1), 2.44(1) and 2.03(1) Å, respectively. Meanwhile, the mean C–C bond distances of the bridging arenes for 2-Y^opt^ and 2-Y are both around 1.46 Å, larger than the typical C–C bond lengths of ∼1.39 Å for a neutral benzene ring;^79^ such C–C distances are also close to the other similar systems.^50,92,93^ The computational results concur with magnetic and spectroscopic data that 2-Ln show Ln(iii)–(C_6_H_6_^4−^)–Ln(iii) configurations. The frontier molecular orbitals of 2-Y^opt^ are depicted in Fig. 7, where both the highest occupied molecular orbital (HOMO) and HOMO−1 show a characteristic δ-bonding feature between the tetraanionic arene bridge and Y(iii) ions.^50,92,93^ These orbitals are close in energy (−3.73 for HOMO and −3.91 eV for HOMO−1), and show respective compositions of 22.8% and 21.7% for the Y(iii) 4d orbitals, and 54.3% and 57.8% for the 2p orbitals of the bound C_6_H_6_^4−^. By contrast the lowest unoccupied molecular orbital (LUMO) consists of weaker π-bonding interactions between the aryl group of the Piso ligands and the Y(iii) centres, with orbital density more localized in the π* orbitals of the terminal arene rings. The LUMO is situated at a much higher energy of −1.16 eV and is comprised of 13.1% 4d orbital character of the Y(iii) ions and 58.1% 2p orbitals of the bound C_6_H_6_^4−^. The δ-bonding feature in these molecular orbitals is similar to that seen previously for other inverse arene f-block complexes, including similar group 3 systems such as [{(BDI)M(THF)_*n*_}_2_(μ-η^6^,η^6^-benzene)] and [[(NN^TBS^)M]_2_(μ-η^6^,η^6^-C_6_H_5_Ph)]^2−^ (M = Y and Sm), *etc.*^50,92,93^

The natural population analysis (NPA) and atoms-in-molecules (AIM) charges were calculated for 2-Y^opt^ (Tables S30 and S31†). The NPA charge on the entire bound arene is −1.43, which is close to other dimeric Ln_2_ complexes possessing such tetraanionic benzene bridges, like [{(BDI)M(THF)_*n*_}_2_(μ-η^6^,η^6^-benzene)] (−1.87 and −1.56 for M = Y and Sm)^50^ and [{(NN^TBS^)M}_2_(μ-η^6^,η^6^-C_6_H_5_Ph)]^2−^ (−1.90 and −1.56 for M = Y and Sm),^92,93^*etc.* Meanwhile, the average NPA charge of Y is 1.24, much lower than 3, which reflects the charge transfer between the Piso and (C_6_H_6_)^4−^ ligands and adjacent Y(iii) ions. In contrast, the corresponding calculated AIM charges show slightly higher values, 1.81 for Y and −1.95 for the whole bound benzene. The Mayer bond orders (MBOs) of the Y–C, Y–N and C–C bonds of the bridging arene in 2-Y^opt^ were calculated (Table S9†). The mean Y–N and Y–C MBOs are 0.15 and 0.14, which agree with polarized covalent bonding, while the bridging C–C arene bonds show MBOs of around 1.08. These latter values are much smaller than those expected for a neutral aromatic ring (MBOs of 1.38–1.46 at different computational levels),^94^ showing that the intra-ring π-bonds have been weakened, and consistent with the longer mean C–C distances and distorted C_6_ ring of C_6_H_6_^4−^.^50,92,93^ Moreover, certain real space functions based on electron densities *ρ*(*r*) at bond critical points (BCPs) were investigated by AIM topology analysis to quantitatively investigate the bonding characteristics of its coordinated bonds (Table S32†).^95,96^ As expected, all BCPs of Y–N and Y–C bonds possess small *ρ*(*r*) values of less than 0.06 a.u., corresponding to typically weak interactions.^97,98^ In particular, the *ρ*(*r*) values at Y–C(C_6_H_6_^4−^) BCPs are comparable to the complex [{(BDI)Sm(THF)_*n*_}_2_(μ-η^6^,η^6^-C_6_H_6_)], where the *ρ*(*r*) of 0.0488 a.u. for the Sm(iii)–C_6_H_6_^4−^ bonding is almost twice as large as those for M(ii)–C_6_H_6_^2−^ bonding (0.0277 a.u. for Ca, 0.0353 a.u. for Yb, 0.0251 a.u. for Sr and 0.0279 a.u. for Sm).^51^ The positive Laplacian functions of the electron density ∇_*ρ*(*r*)_^2^ at the BCPs suggest the electrons are locally depleted, a feature of closed-shell interactions.^99,100^ The negative energy density function *H*(*r*) and the ratio of |*V*(*r*)|/*G*(*r*) larger than one for Y–N and Y–C(C_6_H_6_^4−^) interactions reflect the existence of covalent components in these weak interactions, while the Y–C(Piso) values have the opposite ranges and exhibit more ionic features.^101,102^ The BCPs reveal typical features of dative covalent bonds for Y–C(C_6_H_6_^4−^), demonstrating that the electronic structure of Ln(ii)–(C_6_H_6_)^2−^–Ln(ii) is impossible here where much stronger covalency between Ln and bound arene should be observed.

Time-dependent (TD)-DFT calculations were conducted on 2-Y^opt^ considering the solvent effect from benzene through the SMD solvent model (Table S10†).^103^ As expected, the simulated UV-vis spectrum matches well with the experimental one (Fig. 5 bottom). The calculated transition with the largest oscillator strength (*f*) is located around 583 nm. This absorption has contributions from the excitation of HOMO−1 → LUMO (34.3%), HOMO → LUMO+3 (31.9%) and HOMO → LUMO+1 (21.9%), corresponding to the π → π* transitions from the bridging arenes to the terminal coordinated C_6_ rings. The second strongest transition at 556 nm possesses the same excitation type with HOMO → LUMO+3 (53.4%). The higher energy transitions (<290 nm) are largely localized within Piso-based orbitals, with some excitation to the vacant Y(iii) 4d orbitals.

The magnetic dynamics and relaxation mechanisms of 1-Dy-Cl, 2-Dy, 1-Tb-Cl and 2-Tb were probed by complete active space self-consistent field spin–orbit (CASSCF-SO) calculations.^104,105^ For both dimeric complexes, the single-ion magnetic anisotropy of individual {Ln(Piso)(C_6_H_6_)} fragments were computed. The results revealed Ln(iii) ground states with strong magnetic axiality approaching the Ising limit, with *g*_z_ values close to 20 and 18 for Dy(iii) and Tb(iii), respectively. The highly pure wavefunction compositions of the Ln(iii) ground states are well-described as *m*_J_ = |±15/2> and |±6> for Dy(iii) and Tb(iii), respectively (Tables S11–S16†). The corresponding principal magnetic axes are all coincident with the coordinated nitrogen atoms of the Piso ligands, indicating that they impose a strong crystal field, even stronger than equatorial coordinated chloride in 1-Dy-Cl and 1-Tb-Cl as well as the two arene rings with delocalized electron distributions in 2-Dy and 2-Tb (Fig. S83–S85†). For 1-Tb-Cl and the two fragments of 2-Tb, relatively large tunnelling gaps (*Δ*_tun_) of 0.01 and 0.02 cm^−1^ were calculated between the *m*_J_ = |±6> ground states, which correspond to rather strong QTM (Tables S14–S16†), while superior SMM properties were predicted for 1-Dy-Cl and the two fragments of 2-Dy, with calculated *U*_eff_ values of 585, 495 and 492 K (Fig. S86 and S87†). A relatively small ground-state tunnelling transition moment of 1.77 × 10^−4^*μ*_B_ can be observed in 1-Dy-Cl and this guarantees its zero-field SMM behaviour in ac susceptibility measurements. To determine the intramolecular magnetic couplings in 2-Dy and 2-Tb, the POLY_ANISO program based on the Lines model was used to fit their dc molar magnetic susceptibility data.^106–108^ The best fit gives the coupling parameters listed in Table 2, and the total antiferromagnetic (AFM) interactions (*J*_tot_) of −0.64 (2-Dy) and −6.84 cm^−1^ (2-Tb) originate from the competition between ferromagnetic (FM) dipole–dipole couplings and AFM exchange interactions. We note that the calculated *g*_z_ values of exchange-coupled ground states for 2-Dy and 2-Tb are 26.403 and 7.528, respectively (Tables S26 and S27†), and this deviation from zero is ascribed to the misaligned anisotropic axes of the Ln(iii) ions (Fig. S85†). DFT-BS (broken symmetry) calculations were performed on a Gd(iii) analogue of 2-Y^opt^ (2-Gd*) to verify the AFM contributions of exchange couplings (Table S28†), and after scaling by the corresponding factors, the respective *J*_ex_ values for 2-Dy and 2-Tb are estimated as −5.02 and −3.48 cm^−1^. By contrast, the cycloheptatrienyl trianion (C_7_H_7_^3−^, Cht), of 10π-aromatic system same as (C_6_H_6_)^4−^, is reported to promote significant ferromagnetic exchange coupling between Ln ions in the inverse-sandwich complex [KLn_2_^III^(μ-η^7^:η^7^-Cht){N(SiMe_3_)_2_}_4_] (Ln = Dy, Gd, Er).^109,110^ As described in II (C_6_H_6_)^−^ and (C_6_H_6_)^2−^ feature radical *S* = 1/2 and diradical *S* = 1 and were also shown to transfer extremely strong magnetic exchange coupling, −400 cm^−1^ and −497 cm^−1^ between La(ii) and (C_6_H_6_)^−^/(C_6_H_6_)^2−^ respectively.^42,45^ The exchange and dipolar interactions of 2-Ln can also be compared to the related tetraanionic biphenyl bridged Dy_2_ complexes [{(NN^TBS^)Dy}_2_{(μ-biphenyl)K_2_(solvent)_2_}] (solvent = Et_2_O, toluene, Dy_2_-biph; 18-crown-6, Dy_2_-biph-crown_2_) (Table 2). Both Dy_2_-biph and Dy_2_-biph-crown_2_ show similar structural parameters of the bridging arene^4−^ to 2-Dy, with mean C–C distances of 1.46 Å and torsion angles of 11 and 12°, respectively, and present AFM dipolar coupling and FM exchange coupling.^85^ For 2-Dy and 2-Tb the opposite situation is observed, with FM dipolar and AFM exchange coupling. This discrepancy can be attributed to differences in the N-donor ligands and the amount of electron density in bridged arene^4−^ rings (biphenyl *vs.* benzene) in these complexes,^85^ showing that tetraanionic arene bridges can be designed to promote AFM or FM intramolecular magnetic couplings in Ln_2_ complexes. Despite this, the tunnelling splitting (*Δ*_tun_) for exchange-coupled ground states are large, with magnitudes of ∼10^−6^ (2-Dy) and ∼10^−5^ cm^−1^ (2-Tb) (Tables S26 and S27†), which leads to strong QTM and poor zero-field SMM performance for both complexes.

**Table 2:** The intramolecular magnetic coupling constants in 2-Dy and 2-Tb (cm^−1^), and their comparison to similar tetraanionic biphenyl bridged Dy_2_ complexes Dy_2_-biph and Dy_2_-biph-crown_2_

Complex	POLY_ANISO			DFT-BS
	*J*_ex_	*J*_dip_	*J*_tot_	*J*_ex_
2-Tb	−7.73	0.89	−6.84	−3.48
2-Dy	−1.38	0.74	−0.64	−5.02
Dy_2_-biph^85^	1.06	−2.48	−1.42	—
Dy_2_-biph-crown_2_^85^	7.25	−2.50	4.75	—

## Conclusion

In summary, we have reported straightforward synthetic routes to C_6_ aromatic ring-based arene-capped inverse-sandwich Ln(iii) complexes by the treatment of [Ln(Piso)_2_X] or [Ln(Piso)_2_] with KC_8_ in arene solvents. The Ln(iii) ions in these inverse arene complexes are also bound by pendant amide donor atoms due to κ^1^-N,η^6^-binding of their bulky amidinate ligands. The triple-decker (Dipp)Ln(μ-Arene)Ln(Dipp) frameworks feature near-linear Ln⋯Arene_centroid_⋯Ln and highly bent Arene_centroid_⋯Ln⋯Dipp_centroid_ geometries. Structural parameters, spectroscopic and magnetic data are consistent with the bridging arene rings being formally tetraanionic and the two terminal Dipp rings being neutral. TD-DFT calculations on a model of the Y(iii) analogue showed excellent agreement with experimental UV-vis spectra. Analysis of the DFT-calculated frontier orbitals of [{Y(κ^1^:η^6^-Piso)}_2_(μ-η^6^:η^6^-C_6_H_6_)] indicated that strong Ln-arene δ-bonding interactions are present in this complex, with electron density donated from filled π-orbitals of formal (C_6_H_6_)^4−^ fragments to formally vacant Y(iii) 4d orbitals.

Magnetic studies on paramagnetic [{Ln(κ^1^:η^6^-Piso)}_2_(μ-η^6^:η^6^-C_6_H_6_)] revealed that the bridging benzene tetraanion promotes large AFM interactions between the two Ln(iii) centres, and an exchange coupling parameter of *J*_ex_ = −0.25(1) cm^−1^ was extracted from the *χ*_M_*T* curve of 2-Gd. These results highlight the advantages of the flexible binding mode of the Piso ligand to enable the stabilization of inverse-sandwich Ln arene complexes and provide fresh insights into magnetic communication between two Ln centres promoted by a benzene tetraanion. Arene exchange reactions of [{Y(κ^1^:η^6^-Piso)}_2_(μ-η^6^:η^6^-C_6_H_6_)] with selected PACs in benzene did not proceed under the conditions employed, indicating that these complexes have high kinetic stability, though arene exchange proceeds readily for [{Tb(κ^1^:η^6^-Piso)}_2_(μ-η^6^:η^6^-C_6_H_6_)] in neat toluene. The bridging C_6_H_6_^4−^ ligand functions as an electron reservoir in [{Ln(κ^1^:η^6^-Piso)}_2_(μ-η^6^:η^6^-C_6_H_6_)], and is a potent four electron reductant.

## Experimental Section

### General methods

Dry toluene and pentane were obtained from a solvent purification system, where they were passed through columns containing alumina and molecular sieves, and then stored over K mirrors. Benzene and hexane were dried by refluxing over potassium, and were stored over K mirrors following distillation. All solvents were degassed before use. For NMR spectroscopy C_6_D_6_ was dried by refluxing over K. NMR solvents were vacuum transferred and degassed by three freeze–pump–thaw cycles before use. All experiments were performed under an atmosphere of dry argon with the rigid exclusion of air and moisture, using standard Schlenk and glovebox techniques. ^1^H (400 MHz) and ^13^C{^1^H} (101 MHz) NMR spectra were obtained on an Avance III 400 MHz spectrometer at 298 K. The magnetic susceptibilities of C_6_D_6_ solutions of 1-Ln-Cl and 2-Ln for Ln = Tb and Dy were determined at 298 K by the Evans method;^84^ the lower than expected values obtained for 1-Ln-Cl are attributed to precipitation of sample during the measurement. UV-vis-NIR spectroscopy was performed on samples in Youngs tap-appended 10 mm pathlength quartz cuvettes on an Agilent Technologies Cary Series UV-vis-NIR Spectrophotometer from 175 to 3300 nm. ATR-IR spectra were recorded as microcrystalline powders using a Bruker Alpha II spectrometer. Elemental analysis experiments were performed on an EA 3000 elemental analyser at the Instrument Analysis Center, Xi'an Jiaotong University.

### Single crystal XRD

The crystal data for 1-Ln-X, 2-Ln and 3-Tb are compiled in ESI Tables S1 to S3.† Crystals of 1-Ln-X were collected on an Oxford Diffraction SuperNova Atlas CCD diffractometer using mirror-monochromated MoKα radiation (*λ* = 0.71073 Å), 2-Ln and 3-Tb were collected on a Rigaku XtaLAB Synergy-DW VHF equipped with a HyPix-6000HE photon counting pixel array detector with mirror-monochromated CuKα (*λ* = 1.5418 Å) radiation, and 1-Gd-I and 2-Gd were recorded on a Bruker SMART CCD diffractometer with MoKα radiation (*λ* = 0.71073 Å). All data collections were performed at 100 K. The structures were solved by direct methods and were refined by full-matrix least-squares on all unique *F*^2^ values, with anisotropic displacement parameters for all non-hydrogen atoms, and with constrained riding hydrogen geometries. CrysAlisPro^111^ was used for control and integration, and SHELXT^112,113^ was employed through OLEX2^114^ for structure solution and refinement. Chemcraft and DIAMOND was used for crystallographic images and structural parameters comparasion.^115,116^

### Magnetic measurements

Magnetic measurements were performed on a Quantum Design MPMS3 and PPMS superconducting quantum interference device (SQUID) magnetometer. Samples of 1-Dy-Cl (28 mg), 2-Dy (22.1 mg), 1-Tb-Cl (22.6 mg), 2-Gd (16.0 mg), 2-Tb (27.5 mg) and 2-Y (22.4 mg) were ground to powders, loaded in borosilicate glass NMR tubes, and covered in ground eicosane (10, 10.6, 8.9, 12.5, 4.5, 12.2 and 12.1 mg respectively) in a glovebox. The tubes were removed from the glovebox, kept under a protected atmosphere, and transferred onto a Schlenk line, where the eicosane powder was melted under an argon atmosphere. The tubes were flame-sealed to ∼3 cm under reduced pressure. Kapton tape was wrapped around the top of the tubes, to hold them inside plastic straws by friction, and the straws were attached to the end of the sample rod. The raw data were corrected for the diamagnetic contribution of the sample holder and eicosane using calibrated blanks. The data were corrected for the sample shape using the Quantum Design MPMS3 and PPMS Geometry Simulator resulting in correction values of 0.956–1.039; for each individual sample: height 2.32 mm, diameter 4.08 mm (1-Gd-I), height 3.94 mm, diameter 4.08 mm (1-Tb-Cl), height 2.89 mm, diameter 4.09 mm (2-Tb), height 6.19 mm, diameter 4.09 mm (1-Dy-Cl) and height 4.03 mm, diameter 4.08 mm (2-Dy). Finally, the data were corrected for the intrinsic diamagnetic contribution of the sample, estimated as the molecular weight (g mol^–1^) multiplied by 0.5 × 10^–6^ cm^3^ mol^–1^ K. The dc magnetic susceptibility data of 2-Gd was fitted with eqn (1):1
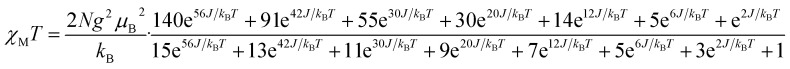


### EPR spectroscopy

Continuous wave EPR spectra were recorded on a Bruker EMX PLUS spectrometer operating at X-band (*ca*. 9.85 GHz). Modulation amplitudes of 4 G were used at microwave powers of 6.325 mW. Spectra were recorded for a reaction mixture containing 1-Y-I and 2 eq. KC_8_ in benzene at room temperature (100 mg for 1-Y-I, 23 mg for KC_8_; the reaction was stopped after 24 h when the mixture gradually changed from colourless to dark blue, and then settled for 3 hours). Approximately 0.3 mL supernatant solution from this reaction mixture was loaded in a 4 mm diameter and 250 mm height J. Young tap quartz tube in an Ar glovebox.

### Calculations

All calculations were performed using Orca 5.0 program.^91^ For geometry optimization calculation towards the model of 2-Y, the hybrid PBE0 functional^117^ was employed with the D3BJ dispersion correction.^118,119^ Scalar relativistic effects were taken into account with the zeroth order regular (ZORA) Hamiltonian.^120^ The SARC-ZORA-TZVP basis set was employed for Y, together with the def2-TZVP basis set for C and N atoms and def2-SVP basis set for H atoms.^121–123^ The SARC/J auxiliary basis set^122^ and the RijCosX approximation^124,125^ were also used throughout. The keyword tightscf was used to improve the convergence threshold and to obtain credible results. The natural population analysis (NPA) was performed with NBO 6.0^126^ and the rest of wavefunction analysis was carried out through Multiwfn program.^127^ The frontier molecular orbitals of 2Y^opt^ were represented and rendered by the visual molecular dynamics (VMD) software.^128^ For TD-DFT calculations, the optimized coordinates of 2-Y^opt^ were used and 75 excited states were considered under SMD solvent model.^103^ In DFT-BS calculations, the same sets were treated to the established model complex 2-Gd*. Ultimately, isotropic exchange coupling constants for 2-Dy and 2-Tb were estimated through the Yamaguchi formula and further scaled for Dy(iii) and Tb(iii) by the factors of 49/25 and 49/36 respectively.^129^

For CASSCF-SO calculations, the individual fragments of 2-Dy and 2-Tb were established according to their X-ray single crystal data without optimization, with one of the paramagnetic Ln(iii) centres replaced with diamagnetic Lu(iii) in each case. Scalar relativistic effects were considered with the second-order Douglas–Kroll–Hess (DKH) Hamiltonian.^130–132^ The SARC2-DKH-QZVP basis set was employed for Dy, Tb and Lu, with the DKH-def2-TZVP basis set used for C and N atoms and the DKH-def2-SVP basis set for H atoms.^123,133^ The keyword AutoAux^134^ and the RijCosX approximation^124,125^ were also used throughout. To save computational resource, only 21 sextets or 7 septets were considered in the state-averaged calculations for Dy(iii) and Tb(iii) complexes, respectively.^104,105^ The static magnetic properties of Dy(iii) or Tb(iii), such as crystal field parameters and *g* tensors were obtained in the SINGLE_ANISO module.^135,136^ The intramolecular exchange and dipole–dipole couplings between both paramagnetic ions in 2-Dy and 2-Tb were then computed by fitting with the experimental dc molar magnetic susceptibilities using POLY_ANISO program.^106–108^

### Synthesis

Anhydrous TbCl_3_, DyCl_3_, YI_3_ and KPiso were prepared according to literature methods,^71–73^ as was KC_8_ (Note: highly reactive and pyrophoric, and must be handled and disposed of with care).^137^ GdI_3_ and cyclooctatetraene was purchased from a commercial source (Aladdin Scientific) and was used as received.

### [GdPiso_2_I]·C_7_H_8_ (1-Gd-I)

GdI_3_ (1.076 g, 2 mmol) and KPiso (1.832 g, 4 mmol) were combined in a 200 mL Rotaflo tap-pressure flask containing a glass-coated magnetic stirring bar, and toluene (50 mL) was added under argon. The reaction mixture was heated gradually to 150 °C with vigorous stirring. After 5 days at 150 °C, the flask was cooled to room temperature, the reaction mixture was filtered, and concentrated under vacuum until copious precipitation of white solid (∼10 mL). The precipitate was re-dissolved by heating the solution to 100 °C. The flask was then wrapped with Al foil and allowed to cool slowly to room temperature overnight. Colourless block crystals of the product formed and were isolated by filtration, washed with cold pentane (2 × 5 mL) and dried under vacuum (1.29 g, 53% based on GdI_3_). C_65_H_94_IN_4_Gd: calcd (%) C 64.22, H 7.79, N 4.61 found (%) C 63.88, H 7.35, N 4.81. UV-vis-NIR *ṽ*_max_/cm^−1^: 34 200 (*ε* = 11 700 M^−1^ cm^−1^). ATR-IR (microcrystalline, cm^−1^): 3339w, 3058w, 2958s, 2868m, 1651w, 1614m, 1584w, 1456m, 1430m, 1408m, 1393w, 1330m, 1305w, 1251w, 1200w, 1168w, 1106w, 1039w, 932m, 826w, 800m, 758s, 716w.

### [TbPiso_2_Cl] (1-Tb-Cl)

TbCl_3_ (0.53 g, 2 mmol) and KPiso (1.832 g, 4 mmol) were combined in a 200 mL Rotaflo tap-appended pressure flask containing a glass-coated magnetic stirring bar, and toluene (50 mL) was added under argon. The reaction mixture was heated gradually to 150 °C with vigorous stirring. After 5 days at 150 °C, the flask was cooled to room temperature, the reaction mixture was filtered, and concentrated under vacuum until copious precipitation of white solid (∼10 mL). The precipitate was re-dissolved by heating the solution to 100 °C. The flask was then wrapped with Al foil and allowed to cool slowly to room temperature overnight. Colourless block crystals of the product formed and were isolated by filtration, washed with cold pentane (2 × 5 mL) and dried under vacuum (0.920 g, 45% based on TbCl_3_). ^1^H and ^13^C{^1^H} NMR spectra could not be interpreted. C_58_H_86_ClN_4_Tb: calcd (%) C 67.39, H 8.39, N 5.42; found (%) C 67.14, H 8.22, N 5.49. *χ*_M_*T* product = 9.15 cm^3^ mol^−1^ K (Evans method). UV-vis-NIR *ṽ*_max_/cm^−1^: 35 200 (*ε* = 8330 M^−1^ cm^−1^). ATR-IR (microcrystalline, cm^−1^): 3339w, 2957s, 2868m, 1614m, 1584w, 1457s, 1424s, 1390m, 1338s, 1299m, 1242w, 1168w, 1099w, 1041w, 932w, 798w, 756s, 744w.

### [DyPiso_2_Cl]·C_5_H_12_ (1-Dy-Cl)

DyCl_3_ (0.54 g, 2 mmol) and KPiso (1.832 g, 4 mmol) were combined in a 200 mL Rotaflo tap-appended pressure flask containing a glass-coated magnetic stirring bar, and toluene (50 mL) was added under argon. The reaction mixture was heated gradually to 150 °C with vigorous stirring. After 5 days at 150 °C, the flask was cooled to room temperature, the reaction mixture was filtered, and concentrated under vacuum until copious precipitation of white solid (∼10 mL). The precipitate was re-dissolved by heating the solution to 100 °C. The flask was then wrapped with Al foil and allowed to cool slowly to room temperature overnight. Colourless block crystals of the product formed and were isolated by filtration, washed with cold pentane (2 × 5 mL) and dried under vacuum (0.770 g, 37% based on DyCl_3_). Crystals of 1-Dy-Cl suitable for single crystal XRD were obtained by recrystallizing from pentane, and elemental analysis results obtained were in agreement with a pentane molecule being present in the lattice. ^1^H and ^13^C{^1^H} NMR spectra could not be interpreted. C_63_H_98_ClN_4_Dy: calcd (%) C 68.20, H 8.83, N 5.05 found (%) C 68.12, H 8.77, N 5.14. *χ*_M_*T* product = 11.12 cm^3^ mol^−1^ K (Evans method). UV-vis-NIR *ṽ*_max_/cm^−1^: 35 200 (*ε* = 8860 M^−1^ cm^−1^). ATR-IR (microcrystalline, cm^−1^): 3345m, 2953s, 2868m, 1614s, 1582m, 1466m, 1436m, 1399w, 1317s, 1242w, 1202w, 1166w, 1099w, 1050w, 926w, 759s.

### [YPiso_2_I]·C_7_H_8_ (1-Y-I)

YI_3_ (0.94 g, 2 mmol) and KPiso (1.832 g, 4 mmol) were combined in a 200 mL Rotaflo tap-pressure flask containing a glass-coated magnetic stirring bar, and toluene (50 mL) was added under argon. The reaction mixture was heated gradually to 150 °C with vigorous stirring. After 5 days at 150 °C, the flask was cooled to room temperature, the reaction mixture was filtered, and concentrated under vacuum until copious precipitation of white solid (∼10 mL). The precipitate was re-dissolved by heating the solution to 100 °C. The flask was then wrapped with Al foil and allowed to cool slowly to room temperature overnight. Colourless block crystals of the product formed and were isolated by filtration, washed with cold pentane (2 × 5 mL) and dried under vacuum (1.060 g, 46% based on YI_3_). ^1^H NMR (400 MHz, C_6_D_6_), *δ*, ppm: 0.83 (s, 18H, C(C*H*_3_)_3_), 1.16 (d, ^3^*J*_HH_ = 6.8 Hz, 12H, CH(C*H*_3_)_2_), 1.28 (d, ^3^*J*_HH_ = 6.8 Hz, 36H, CH(C*H*_3_)_2_), 3.57 (m, 8H, C*H*(CH_3_)_2_), 6.99 (m, 12H, C_6_*H*_3_-aryl). ^13^C{^1^H} NMR (101 MHz, C_6_D_6_), *δ*, ppm: 23.42 (CH(*C*H_3_)_2_), 28.74 (C(*C*H_3_)_3_), 30.92 (*C*H(CH_3_)_2_), 44.63 (*C*(CH_3_)_3_), 123.94, 125.31, 128.18, 143.10 (*C*_6_H_3_-aryl), 182.69 (d, ^2^*J*_YC_ = 2.0 Hz, N–*C*–N). C_65_H_94_IN_4_Y: calcd (%) C 68.05, H 8.26, N 4.88 found (%) C 67.74, H 8.19, N 4.92. UV-vis-NIR *ṽ*_max_/cm^−1^: 36 200 (*ε* = 6820 M^−1^ cm^−1^). ATR-IR (microcrystalline, cm^−1^): 3340w, 2959m, 2868m, 1615s, 1585m, 1459m, 1431m, 1410m, 1393w, 1329m, 1306w, 1244w, 1201w, 1169w, 1096w, 1040w, 945m, 827w, 700m, 768s, 724s.

### [{Gd(κ^1^:η^6^-Piso)}_2_(μ-η^6^:η^6^-C_6_H_6_)] (2-Gd)

A solution of 1-Gd-I (0.607 g, 0.5 mmol) in benzene (20 mL) was added dropwise to a stirred suspension of KC_8_ (0.203 g, 1.5 mmol) in benzene (10 mL) at room temperature. The mixture was allowed to stir at 25 °C for 3 days (reaction mixture gradually changed from colourless to blue and bronze KC_8_ slowly converted to black graphite). The reaction mixture was allowed to settle overnight and filtered to another flask. The solvent was removed under vacuum and the residue was extracted by stirring with hexane (25 mL) for 15 min. The solution was filtered and concentrated to ∼5 mL under vacuum, then left at −35 °C to crystallize. Dark blue octahedral crystals of 2-Gd were isolated after several days (0.132 g, 43% based on 1-Gd-I). C_64_H_92_N_4_Gd_2_: Anal. Calcd (%) C 62.40, H 7.53, N 4.55; found (%) C 62.12, H 7.45, N 4.62. UV-vis-NIR *ṽ*_max_/cm^−1^: 17 120 (*ε* = 1296 M^−1^ cm^−1^), 34 200 (*ε* = 22 200 M^−1^ cm^−1^). ATR-IR (microcrystalline, cm^−1^): 3340w, 2957s, 2868m, 1615s, 1585w, 1462w, 1430w, 1392m, 1382m, 1361m, 1320m, 1243w, 1209w, 1187w, 1096w, 945m, 799w, 767s, 756s, 723w.

### [{Tb(κ^1^:η^6^-Piso)}_2_(μ-η^6^:η^6^-C_6_H_6_)] (2-Tb)

A solution of 1-Tb-Cl (0.517 g, 0.5 mmol) in benzene (20 mL) was added dropwise to a stirred suspension of KC_8_ (0.203 g, 1.5 mmol) in benzene (10 mL) at room temperature. The mixture was allowed to stir at 25 °C for 3 days (reaction mixture gradually changed from colourless to blue and bronze KC_8_ slowly converted to black graphite). The reaction mixture was allowed to settle overnight and filtered to another flask. The solvent was removed under vacuum and the residue was extracted by stirring with hexane (25 mL) for 15 min. The solution was filtered and concentrated to ∼5 mL under vacuum, then left at −35 °C to crystallize. Dark blue octahedral crystals of 2-Tb were isolated after several days (0.145 g, 47% based on 1-Tb-Cl). Alternative method: 2-Tb can be also synthesized by the reaction between TbPiso_2_ (0.1 g, 0.1 mmol) and KC_8_ (0.027 g, 0.2 mmol) in benzene (0.051 g, 83% based on TbPiso_2_). ^1^H NMR (400 MHz, C_6_D_6_), *δ*, ppm: −91.37 (s, 18H, C(C*H*_3_)_3_), 1.19 (d, *J* = 145.3 Hz, 12H, CH(C*H*_3_)_2_), 34.99 (s, 8H, C*H*(CH_3_)_2_), 52.44 (s, 18H, CH(C*H*_3_)_2_), 51.69 (s, 18H, CH(C*H*_3_)_2_), 63.67 (s, 6H, C_6_*H*_6_^4−^), 131.57 (br, 4H, *p*-C_6_*H*_3_-aryl), 250.12 (br, 8H, *m*-C_6_*H*_3_-aryl). ^13^C{^1^H} NMR spectra could not be interpreted. C_64_H_92_N_4_Tb_2_: Anal. Calcd (%) C 62.23, H 7.51, N 4.54; found (%) C 61.52, H 7.15, N 4.60. *χ*_M_*T* product = 23.24 cm^3^ mol^−1^ K (Evans method). UV-vis-NIR *ṽ*_max_/cm^−1^: 17 240 (*ε* = 8340 M^−1^ cm^−1^), 35 590 (*ε* = 16 280 M^−1^ cm^−1^). ATR-IR (microcrystalline, cm^−1^): 3336w, 2957s, 2862m, 1621m, 1582w, 1439s, 1378s, 1353s, 1308w, 1250w, 1199w, 1166m, 1099w, 1020w, 941w, 795m, 759s, 717w.

### [{Dy(κ^1^:η^6^-Piso)}_2_(μ-η^6^:η^6^-C_6_H_6_)]·C_6_H_14_ (2-Dy)

A solution of 1-Dy-Cl (0.519 g, 0.5 mmol) in benzene (20 mL) was added dropwise to a stirred suspension of KC_8_ (0.203 g, 1.5 mmol) in benzene (10 mL) at room temperature. The mixture was allowed to stir at 25 °C for 3 days (reaction mixture gradually changed from colourless to purple and bronze KC_8_ slowly converted to black graphite). The reaction mixture was allowed to settle overnight and filtered to another flask. The solvent was removed under vacuum and the residue was extracted by stirring with hexane (25 mL) for 15 min. The solution was filtered and concentrated to ∼5 mL under vacuum, then left at −35 °C to crystallize. Dark purple octahedral crystals of 2-Dy were isolated after several days (0.156 g, 49% based on 1-Dy-Cl). Alternative method: 2-Dy can be also synthesized by the reaction between DyPiso_2_ (0.1 g, 0.1 mmol) and KC_8_ (0.027 g, 0.2 mmol) in benzene (0.05 g, 79% based on DyPiso_2_). ^1^H NMR (400 MHz, C_6_D_6_) spectra could not be interpreted, *δ*, ppm: 0.85 (m, 40H, C(C*H*_3_)_3_), 3.55 (d, *J* = 70.7 Hz, 2H), 5.57 (s, 1H), 115.19 (s, 7H), 205.02 (s, 10H). ^13^C{^1^H} NMR spectra could not be interpreted. C_70_H_106_N_4_Dy_2_: Anal. Calcd (%) C 63.20, H 8.04, N 4.22; found (%) C 62.91, H 7.88, N 4.35. *χ*_M_*T* product = 28.24 cm^3^ mol^−1^ K (Evans method). UV-vis-NIR *ṽ*_max_/cm^−1^: 17 540 (*ε* = 2180 M^−1^ cm^−1^), 35 840 (*ε* = 13 020 M^−1^ cm^−1^). ATR-IR (microcrystalline, cm^−1^): 2959s, 2866m, 1478s, 1412s, 1341s, 1308m, 1238w, 1199w, 1153s, 1089m, 1038w, 938m, 843w, 783m, 756m, 719s, 653w, 628w, 562w.

### [{Y(κ^1^:η^6^-Piso)}_2_(μ-η^6^:η^6^-C_6_H_6_)]·C_6_H_14_ (2-Y)

A solution of 1-Y-I (0.574 g, 0.5 mmol) in benzene (20 mL) was added dropwise to a stirred suspension of KC_8_ (0.203 g, 1.5 mmol) in benzene (10 mL) at room temperature. The mixture was allowed to stir at 25 °C for 3 days (reaction mixture gradually changed from colourless to blue and bronze KC_8_ slowly converted to black graphite). The reaction mixture was allowed to settle overnight and filtered to another flask. The solvent was removed under vacuum and the residue was extracted by stirring with hexane (25 mL) for 15 min. The solution was filtered and concentrated to ∼5 mL under vacuum, then left at −35 °C to crystallize. Dark blue octahedral crystals of 2-Y were isolated after several days (0.160 g, 56% based on 1-Y-I). ^1^H NMR (400 MHz, C_6_D_6_), *δ*, ppm: 1.34 (d, ^3^*J*_HH_ = 6.9 Hz, 48H, CH(C*H*_3_)_2_), 1.48 (s, 18H, C(C*H*_3_)_3_), 2.05 (s, 6H, C_6_*H*_6_^4−^), 3.32 (m, 8H, C*H*(CH_3_)_2_), 5.88 (t, ^1^*J*_YH_ = 7.6 Hz, 2H, *p*-C_6_*H*_3_-aryl), 6.73 and 6.91 (d, ^3^*J*_HH_ = 7.6 Hz, 4H, *m*-C_6_*H*_3_-aryl), 6.99 (dt, ^3^*J*_HH_ = 7.7 Hz, ^1^*J*_YH_ = 4.2 Hz, 2H, *p*-C_6_*H*_3_-aryl). ^13^C{^1^H} NMR (101 MHz, C_6_D_6_), *δ*, ppm: 13.94, 20.10, 22.38, 23.58, 24.63, 26.31, 28.07, 28.67 (CH(*C*H_3_)_2_), 31.75 (C(*C*H_3_)_3_), 34.08 (*C*H(CH_3_)_2_), 41.95 (*C*(CH_3_)_3_), 65.51 (t, ^1^*J*_YC_ = 5.1 Hz, *C*_6_H_6_^4−^), 111.61, 115.83, 122.80, 139.51 (*C*_6_H_3_-aryl), 123.77, 142.66, 145.12, 157.09 (Y–*C*_6_H_3_-aryl), 172.29 (N–*C*–N). C_70_H_106_N_4_Y_2_: Anal. Calcd (%) C 71.16, H 9.04, N 4.74; found (%) C 70.96, H 9.34, N 4.65. UV-vis-NIR *ṽ*_max_/cm^−1^: 17 240 (*ε* = 11 500 M^−1^ cm^−1^), 35 970 (*ε* = 21 400 M^−1^ cm^−1^). ATR-IR (microcrystalline, cm^−1^): 3340w, 2959m, 2868m, 1616s, 1585m, 1461m, 1431m, 1411m, 1394w, 1361w, 1341m, 1330m, 1307w, 1243w, 1201w, 1096w, 1040w, 946w, 932m, 827w, 800m, 767s, 716w.

### [{Tb(κ^1^:η^6^-Piso)}_2_(μ-η^6^:η^6^-C_7_H_8_)]·2C_5_H_12_ (3-Tb)

2-Tb (0.050 g, 0.04 mmol) was dissolved in toluene (10 mL) and then stirred for 24 hours at room temperature to give a blue solution. The solvent was removed under vacuum and the residue was extracted by stirring with pentane (25 mL) for 15 min. The solution was filtered and concentrated to ∼2 mL under vacuum, then left at −35 °C to crystallize. Dark blue crystals of 3-Tb were isolated after several days (0.041 g, 74% based on 2-Tb). Alternative method: 3-Tb can be also independently synthesized by stirring 1-Tb-Cl (0.103 g, 0.1 mmol) and KC_8_ (0.04 g, 0.3 mmol) in toluene for 3 days, as determined by the formation of a dark blue reaction mixture. C_74_H_118_N_4_Tb_2_: Anal. Calcd (%) C 64.33, H 8.61, N 4.05; found (%) C 63.54, H 8.47, N 4.02. UV-vis-NIR *ṽ*_max_/cm^−1^: 16 670 (*ε* = 970 M^−1^ cm^−1^), 36 100 (*ε* = 9100 M^−1^ cm^−1^). ATR-IR (microcrystalline, cm^−1^): 3339w, 2953m, 2870m, 1615s, 1584m, 1411w, 1394w, 1330m, 1307w, 1244w, 1201w, 1170m, 1158m, 1096w, 1054w, 1040w, 932m, 882w, 801m, 768s, 715w.

### Arene exchange reactions of 2-Y

Separate mixtures of 2-Y (23 mg, 0.02 mmol) and or biphenyl (3 mg, 0.02 mmol) or naphthalene (2.6 mg, 0.02 mmol) or anthracene (3.6 mg, 0.02 mmol) were dissolved in C_6_D_6_ (*ca.* 0.6 mL) and the dark blue solution was transferred to a J. Young NMR tube. Arene exchange reactions were also conducted between 2-Y (12 mg, 0.01 mmol) and C_6_D_6_ (0.6 mL) or C_7_D_8_ (0.6 mL). The arene exchange reactions were monitored by ^1^H NMR spectroscopy at 80 °C over the course of 100–150 h. There was no exchange when C_6_D_6_ solutions of 2-Y were separately treated with biphenyl, naphthalene or anthracene after 150 h (Fig. S15–S18†), and no arene exchange was observed between 2-Y and C_6_D_6_ or C_7_D_8_, in spite of some minor decomposition (Fig. S19†). The reaction between 2-Y (12 mg, 0.01 mmol) and an excess of toluene (5 mL) was also performed in an Rotaflo tap-appended ampoule. The reaction mixture was allowed to stir for 12 hours at room temperature, then toluene was removed under vacuum and the residue was dissolved in 0.6 mL C_6_D_6_ for ^1^H and ^13^C{^1^H} NMR spectroscopy, where the arene exchange was shown to have occurred with a signal corresponding to (C_7_H_8_)^4−^ detected (Fig. S13 and S14†). Alternative method: 3-Y can also be independently synthesized by stirring 1-Y-I (0.11 g, 0.1 mmol) and KC_8_ (0.04 g, 0.3 mmol) in toluene for 3 days, as determined by the formation of a dark blue reaction mixture.

### Reactions of 2-Y with COT

Two separate reactions were conducted between 2-Y and COT with molar ratios of 1 : 2 and 1 : 4. C_6_D_6_ (0.3 mL) solutions of COT (2 mg, 0.02 mmol; or 4 mg, 0.04 mmol) were separately added dropwise to two C_6_D_6_ (0.3 mL) solutions of 2-Y (12 mg, 0.01 mmol) in J. Young NMR tubes. The reaction mixtures were agitated by hand and the dark blue reaction mixtures changed colour to pale yellow within 5 min. For the 1 : 2 reaction: ^1^H NMR (400 MHz, C_6_D_6_), *δ*, ppm: 0.62 (s, 9H, C(C*H*_3_)_3_), 1.20 (d, ^3^*J*_HH_ = 6.67 Hz, 12H, CH(C*H*_3_)_2_), 1.32 (d, ^3^*J*_HH_ = 6.83 Hz, 12H, CH(C*H*_3_)_2_), 2.88 (m, 4H, C*H*(CH_3_)_2_), 5.63 (s, C_8_*H*_10_, COT), 6.60 (s, 8H, C_8_*H*_8_, COT^2−^), 7.00 (m, 6H, C_6_*H*_3_-aryl); For 1 : 4 reaction: ^1^H NMR (400 MHz, C_6_D_6_), *δ*, ppm: 0.61 (s, 9H, C(C*H*_3_)_3_), 1.21 (d, ^3^*J*_HH_ = 6.77 Hz, 12H, CH(C*H*_3_)_2_), 1.33 (d, ^3^*J*_HH_ = 6.84 Hz, 12H, CH(C*H*_3_)_2_), 2.88 (m, 4H, C*H*(CH_3_)_2_), 5.63 (s, 10H, C_8_*H*_10_, COT), 6.60 (s, 8H, C_8_*H*_8_, COT^2−^), 6.99 (m, 6H, C_6_*H*_3_-aryl).

## Data availability

NMR spectra, X-ray crystallographic data, UV-vis-NIR and IR spectra, magnetic data, EPR spectroscopy and DFT calculation details are documented in the ESI.† CCDC 2402307 (1-Gd-I), 2370381 (1-Tb-Cl), 2370379 (1-Dy-Cl), 2370380 (1-Y-I), 2402308 (2-Gd), 2370378 (2-Tb), 2370382 (2-Dy), 2370383 (2-Y) and 2370384 (3-Tb) contain the ESI† crystallographic data for this paper. These data can be obtained free of charge *via*https://www.ccdc.cam.ac.uk/data_request/cif, or by emailing data_request@ccdc.cam.ac.uk, or by contacting The Cambridge Crystallographic Data Centre, 12 Union Road, Cambridge, CB2 1EZ, UK; fax: +44 1223 336033.

## Author contributions

P.-B. J. synthesized and characterized the complexes. P.-B. J. collected, solved, and refined the single crystal XRD data. G. K. G. and P.-B. J. performed SQUID magnetometry measurements. P.-B. J. and Q.-C. L. interpreted the magnetic data. Q.-C. L. performed the theoretical calculations and interpretation. R. E. P. W., D. P. M. and Y.-Z. Z. supervised the study. P.-B. J., Q.-C. L. and D. P. M. wrote the manuscript with input from all the authors.

## Conflicts of interest

The authors declare no competing interests.

## Supplementary Material

SC-016-D4SC05982D-s001

SC-016-D4SC05982D-s002
