# Supplementary material for: Rare earth benzene tetraanion-bridged amidinate complexes
Source: Chem Sci. 2024 Dec 17;16(4):1907–24. doi: 10.1039/d4sc05982d (PMC11667736; doi:10.1039/d4sc05982d)
Supplement: SC-016-D4SC05982D-s001 [file SC-016-D4SC05982D-s001.pdf]

Supplementary Information (SI) for Chemical Science.
This journal is © The Royal Society of Chemistry 2024

Rare Earth Benzene Tetraanion-Bridged Amidinate Complexes

Peng-Bo Jin,[a,†] Qian-Cheng Luo,[a,†] Gemma K. Gransbury,[b] Richard E. P. Winpenny,*[,b] David P. Mills,*[,b] and Yan-Zhen Zheng*[,a]

[a]*Frontier Institute of Science and Technology, State Key Laboratory of Electrical Insulation and Power Equipment, MOE Key Laboratory for Nonequilibrium Synthesis of Condensed Matter, Xi'an Key Laboratory of Electronic Devices and Materials Chemistry and School of Chemistry, Xi'an Jiaotong University, 99 Yanxiang Road, Xi'an, Shaanxi 710054, P. R. China.*

[b]*Department of Chemistry, The University of Manchester, Oxford Road, Manchester, M13 9PL, U.K.*

[†]*These authors contributed equally to this work.*

**Email: richard.winpenny@manchester.ac.uk; david.mills@manchester.ac.uk; zheng.yanzhen@xjtu.edu.cn*

Table of Contents

1. NMR spectra **S3**

2. Infrared spectra **S18**

3. UV-vis-NIR spectroscopy **S23**

4. Crystallography **S35**

5. Magnetism **S42**

5.1 Temperature-swept magnetic measurements **S42**

5.2 Ac magnetic measurements **S45**

5.3 Magnetic relaxation profiles **S49**

5.4 Field-swept magnetic measurements **S54**

6. EPR spectroscopy **S58**

7. Calculations **S59**

8. References **S85**

1. NMR spectra

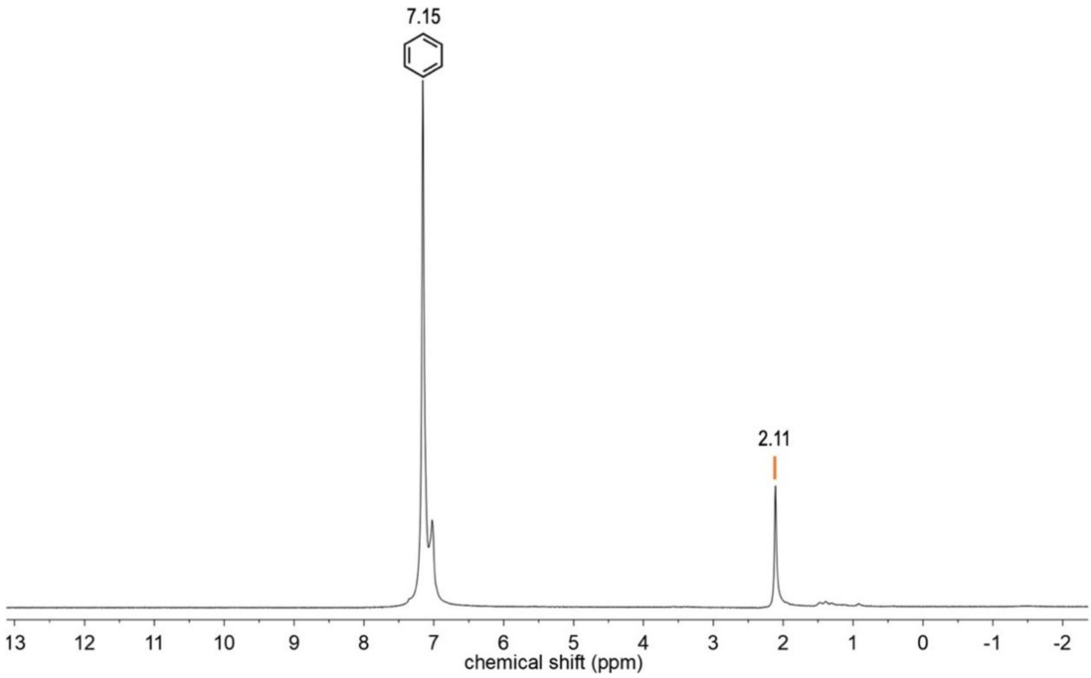

Figure S1 ^{1}H NMR spectrum of **1-Tb-Cl** in C_6D_6 in the region -200 to 200 ppm, zoomed in -2 to 13 ppm.

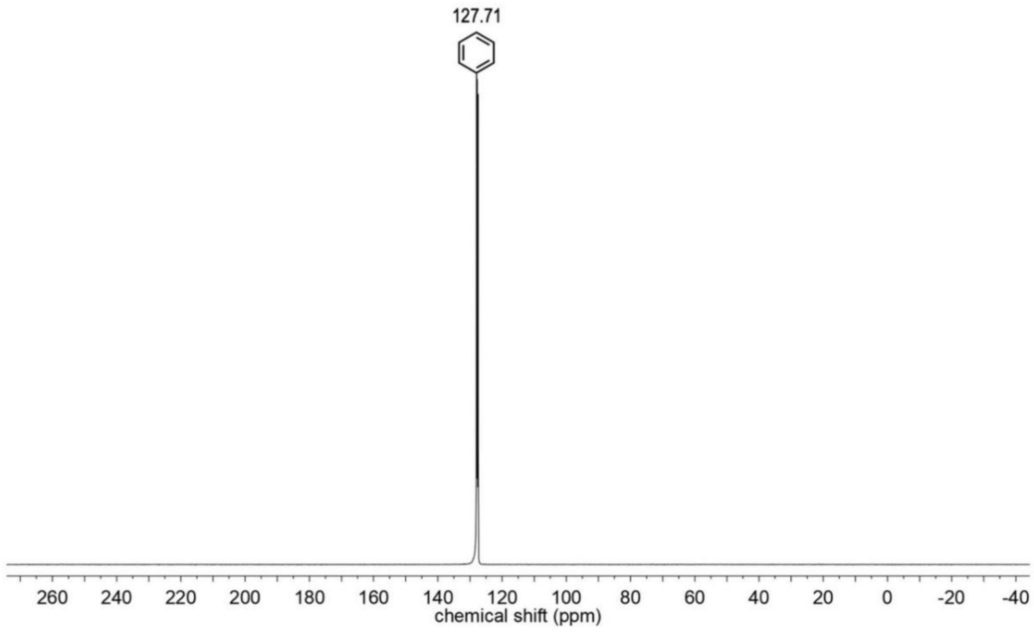

Figure S2 $^{13}C\{^{1}H\}$ NMR spectrum of **1-Tb-Cl** in C_6D_6 in the region -40 to 270 ppm.

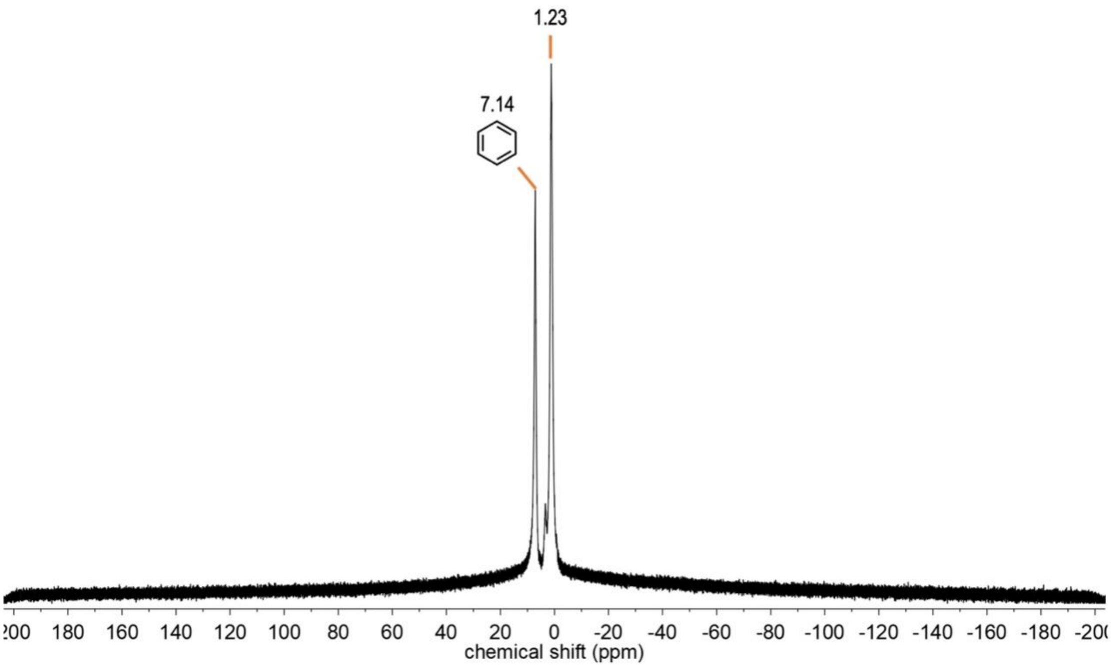

Figure S3 ^{1}H NMR spectrum of **1-Dy-Cl** in C_6D_6 in the region -200 to 200 ppm.

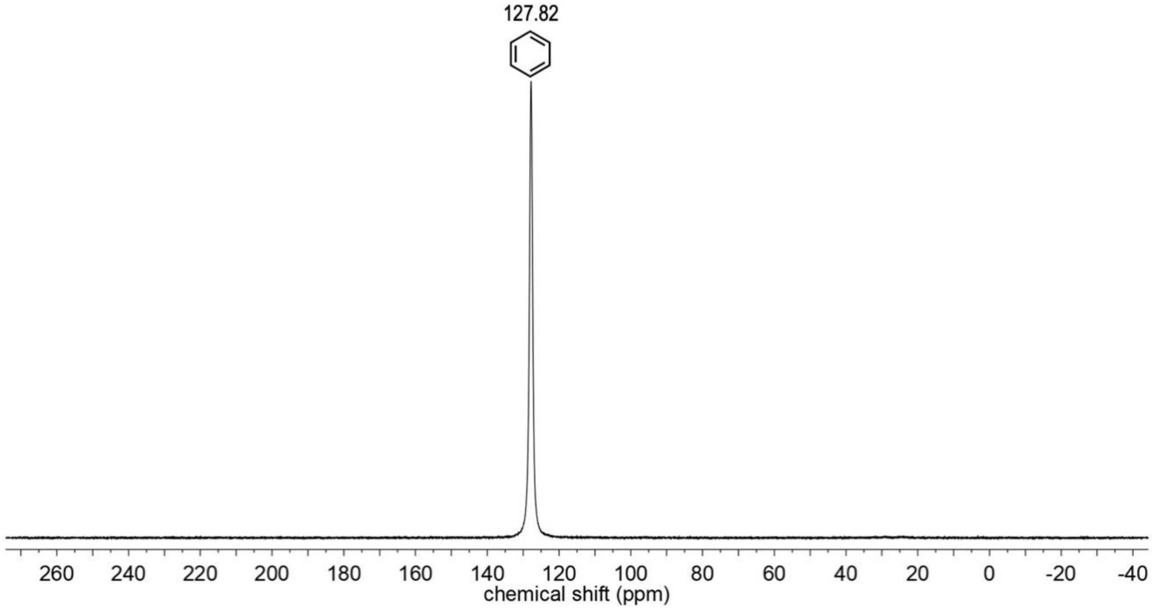

Figure S4 $^{13}C\{^{1}H\}$ NMR spectrum of **1-Dy-Cl** in C_6D_6 in the region -40 to 270 ppm.

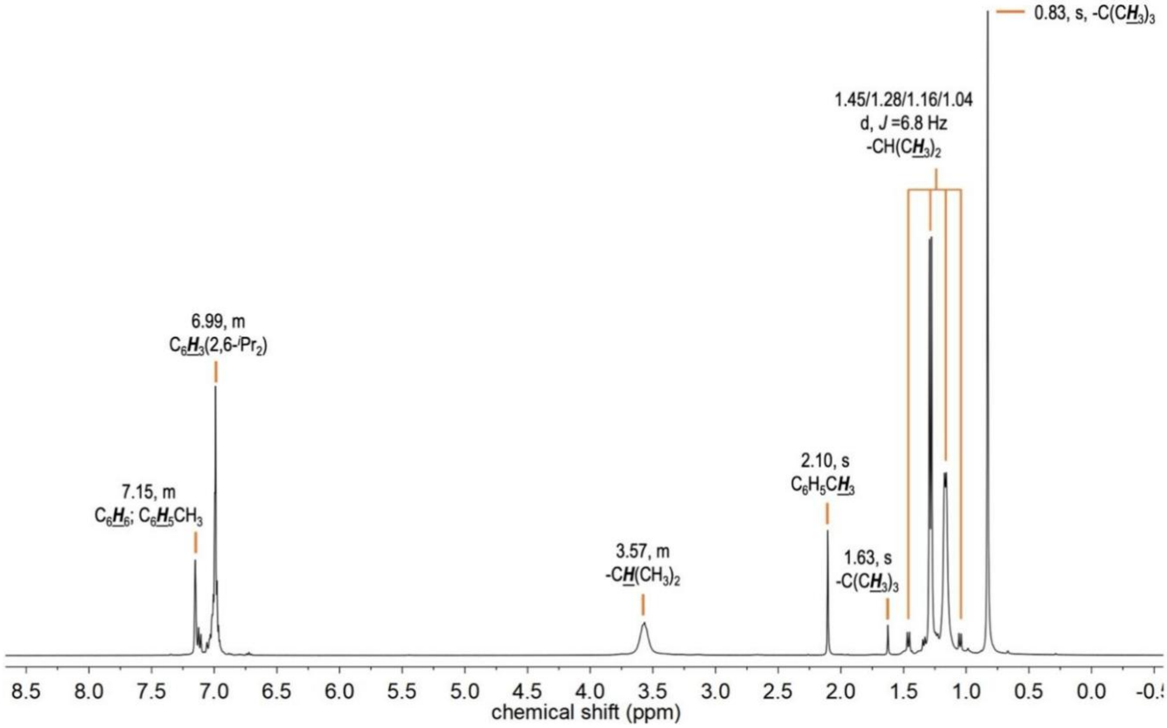

Figure S5 ^{1}H NMR spectrum of **1-Y-I** in C_6D_6 in the region -4 to 16 ppm, zoomed in 0 to 9 ppm.

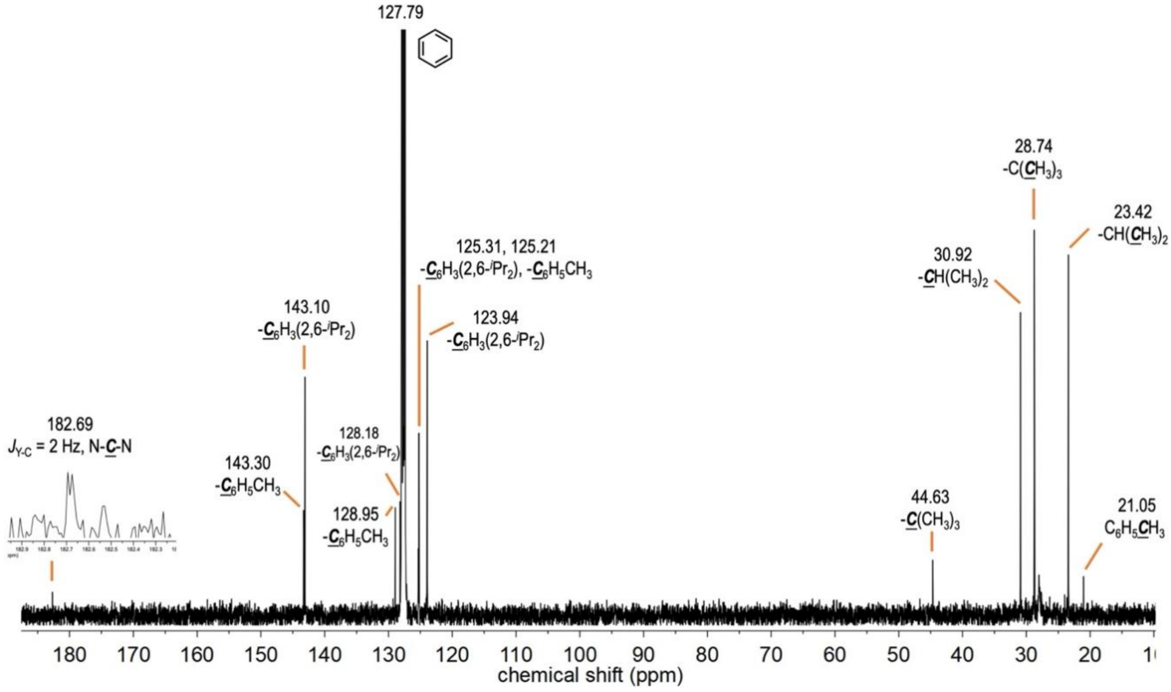

Figure S6 $^{13}C\{^{1}H\}$ NMR spectrum of **1-Y-I** in C_6D_6 in the region -60 to 260 ppm, zoomed in 10 to 190 ppm.

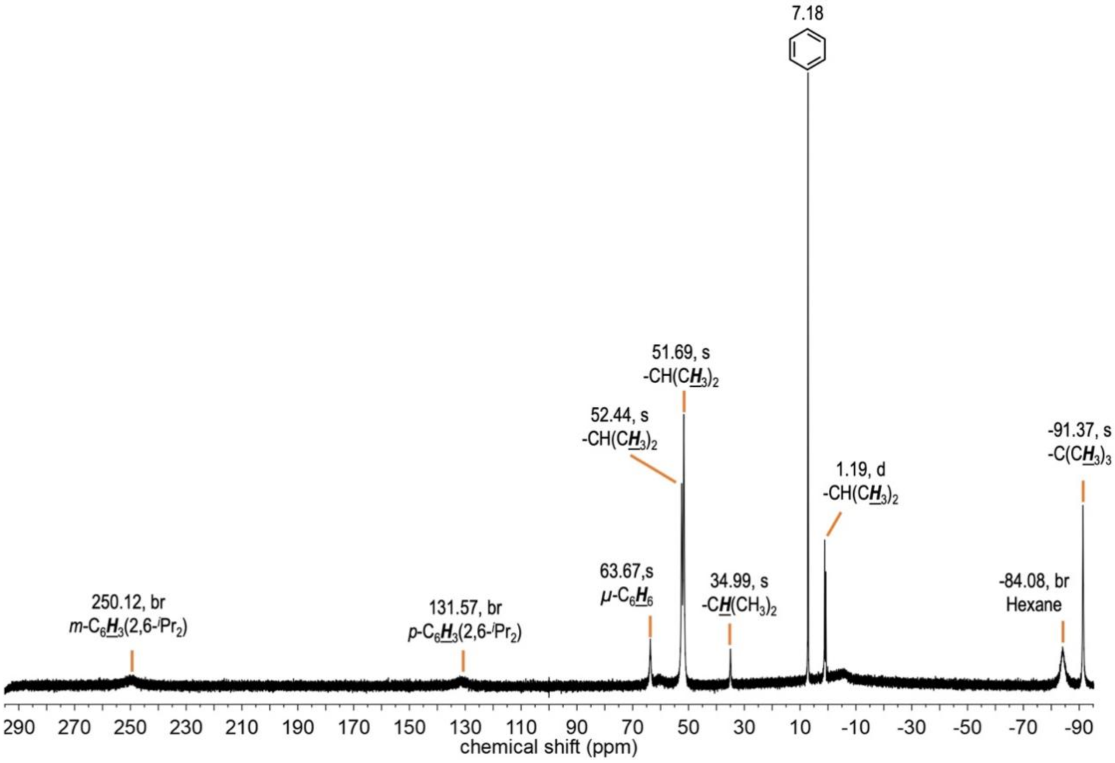

Figure S7 ^{1}H NMR spectrum of **2-Tb** in C_6D_6 in the region -90 to 290 ppm.

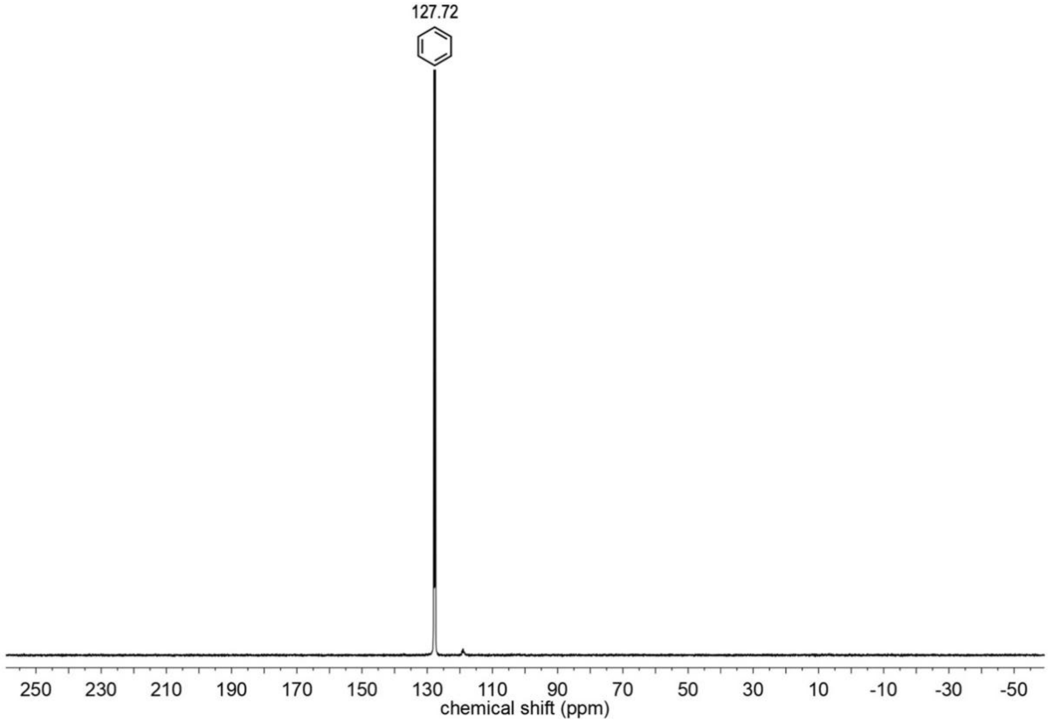

Figure S8 ^{13}C{^{1}H} NMR spectrum of **2-Tb** in C_6D_6 in the region -60 to 260 ppm.

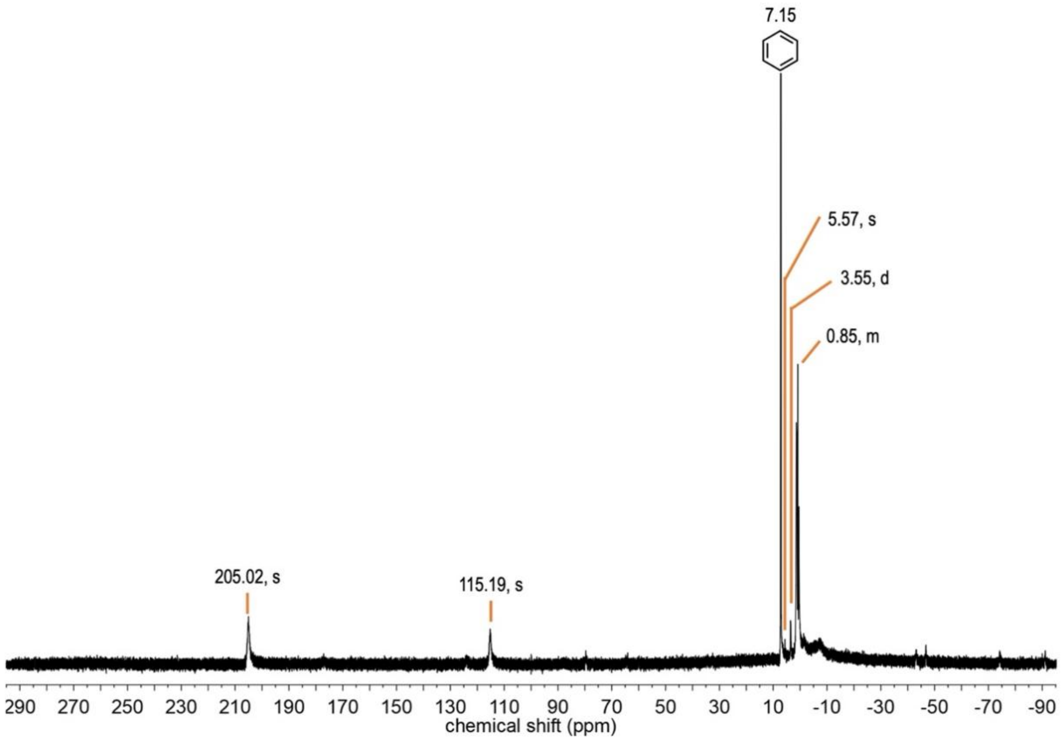

Figure S9 ^{1}H NMR spectrum of **2-Dy** in C_6D_6 in the region -90 to 290 ppm.

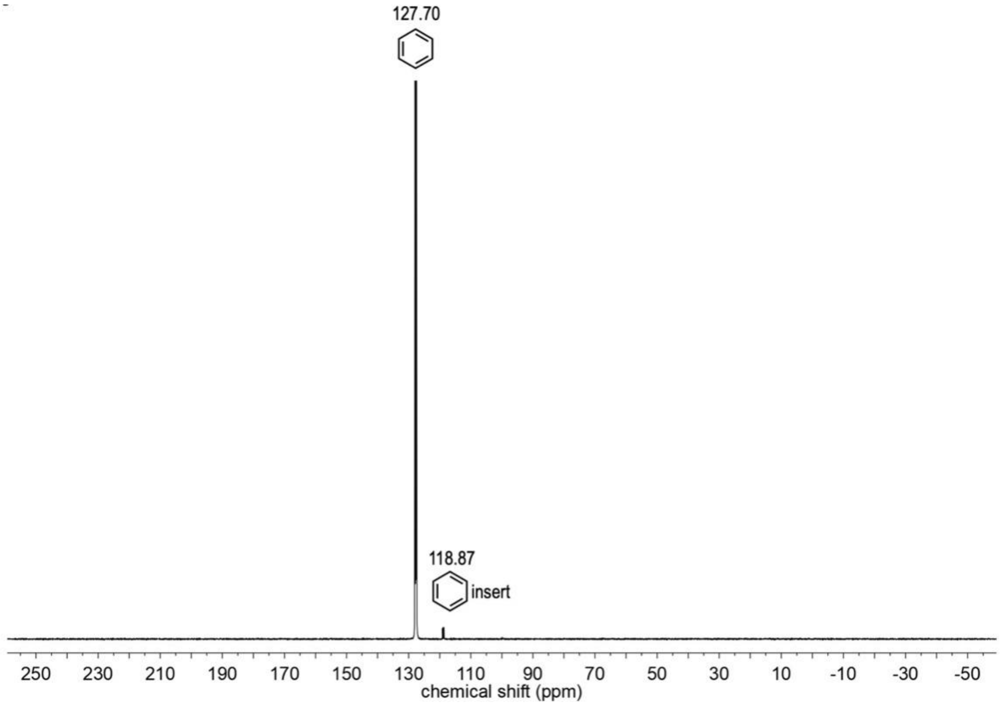

Figure S10 $^{13}C\{^{1}H\}$ NMR spectrum of **2-Dy** in C_6D_6 in the region -60 to 260 ppm.

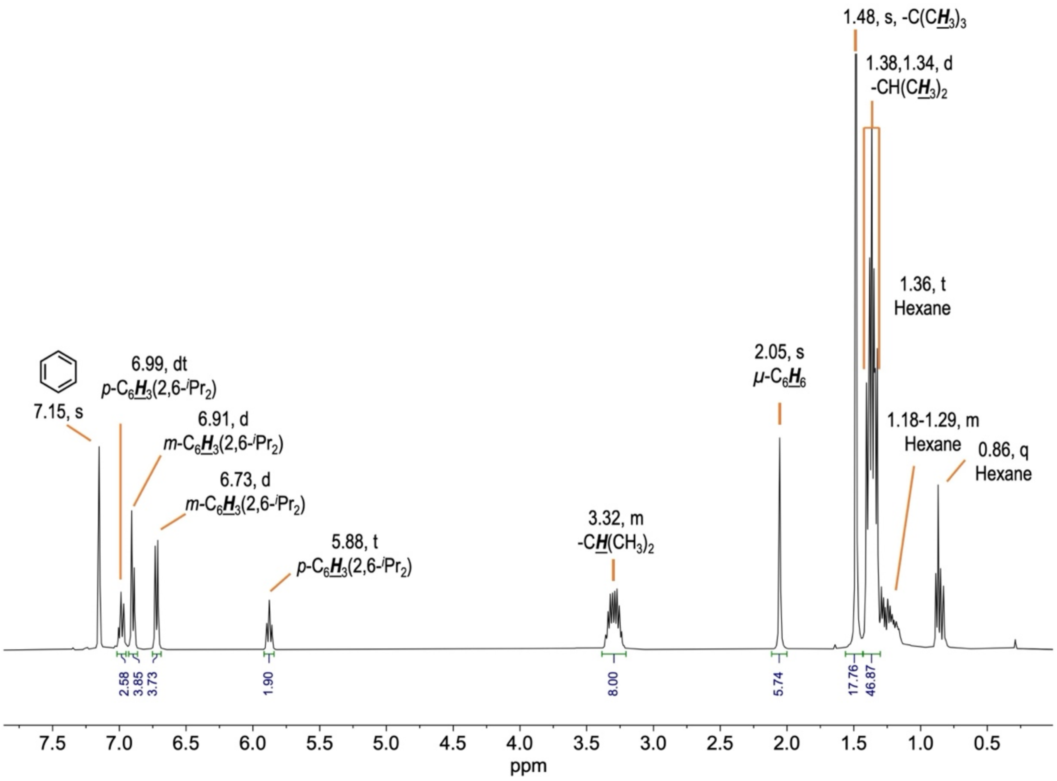

Figure S11 ^{1}H NMR spectrum of **2-Y** in C_6D_6 in the region -4 to 16 ppm, zoomed in 0 to 8 ppm.

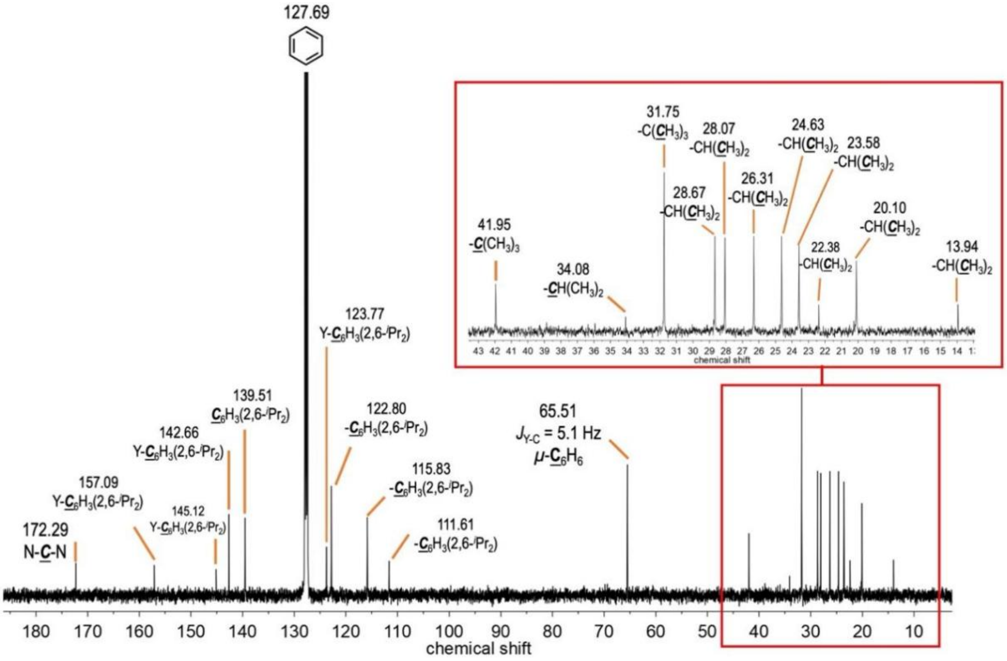

Figure S12 $^{13}C\{^{1}H\}$ NMR spectrum of **2-Y** in C_6D_6 in the region -60 to 260 ppm, zoomed in 0 to 190 ppm.

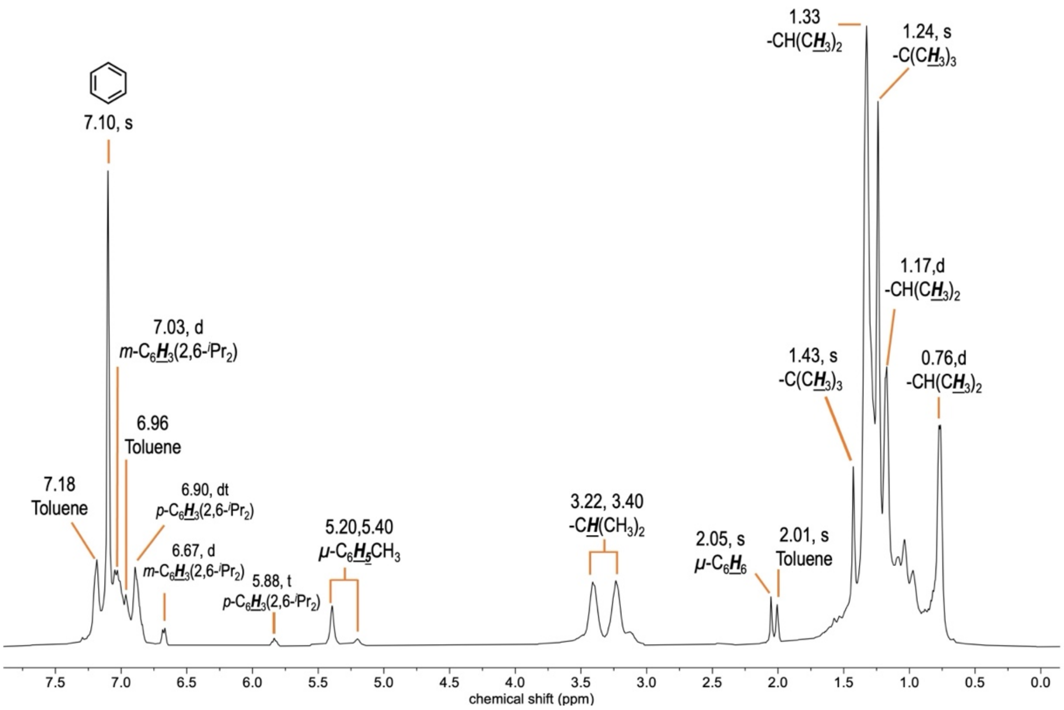

Figure S13 ^{1}H NMR spectrum in C_6D_6 of an arene exchange reaction of **2-Y** treated with 5 mL toluene for 12 hours in the region -4 to 16 ppm, zoomed in 0 to 8 ppm.

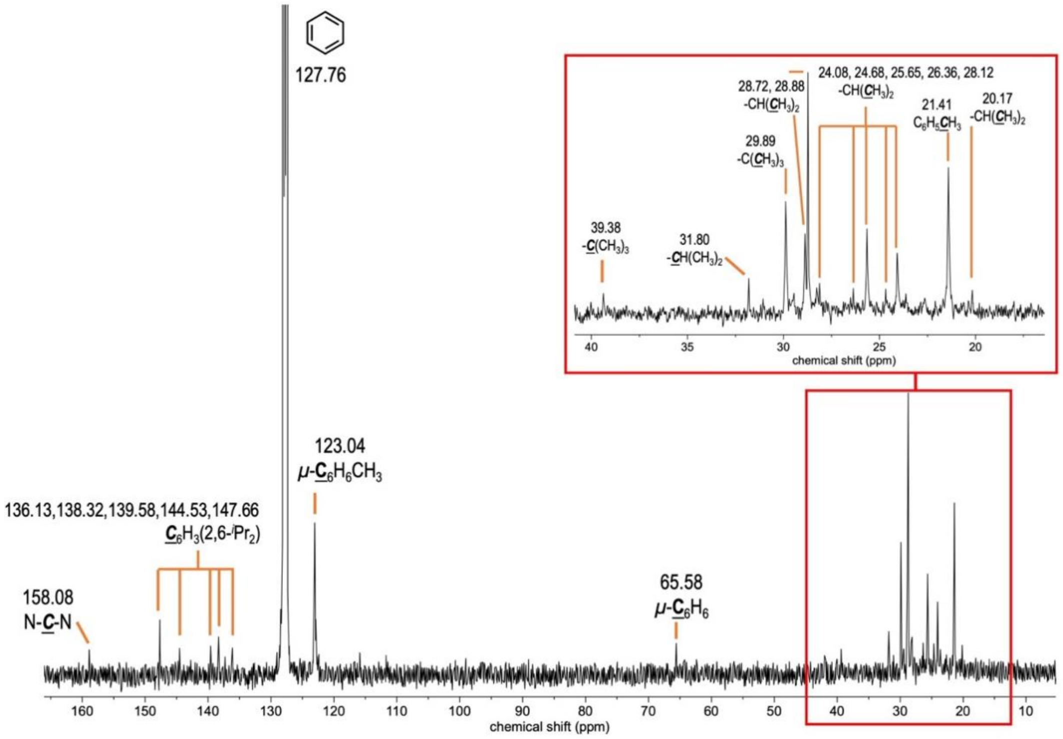

Figure S14 $^{13}C\{^{1}H\}$ NMR spectrum in C_6D_6 of an arene exchange reaction of **2-Y** treated with 5 mL toluene for 12 hours in the region -60 to 260 ppm, zoomed in 0 to 170 ppm.

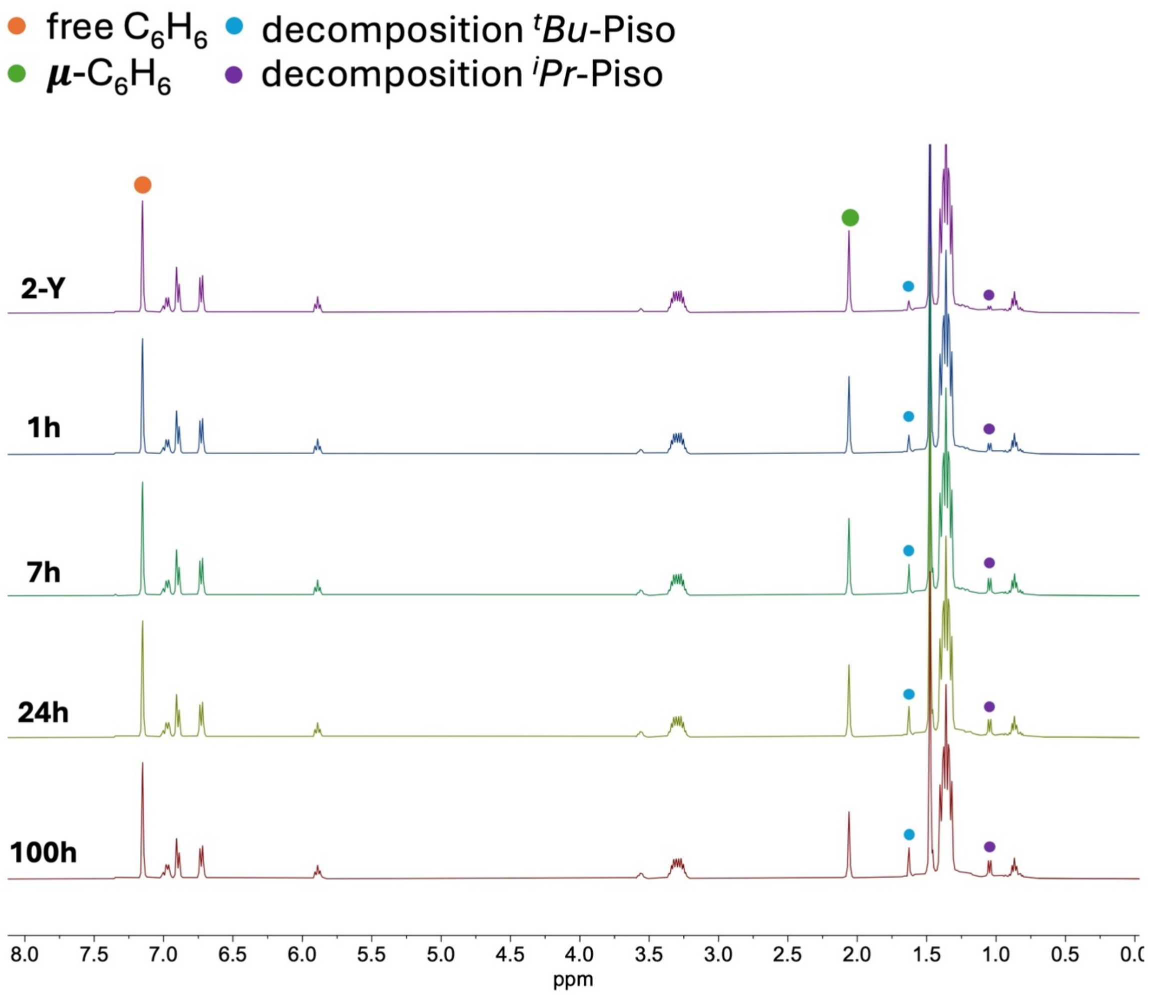

Figure S15 Stacked ^1H NMR spectra showing time evolution of a solution of **2-Y** in C_6D_6 heated at 80 °C for up to 100 h in the region 0 to 8 ppm. No arene exchange was observed between **2-Y** and C_6D_6, just some minor decomposition.

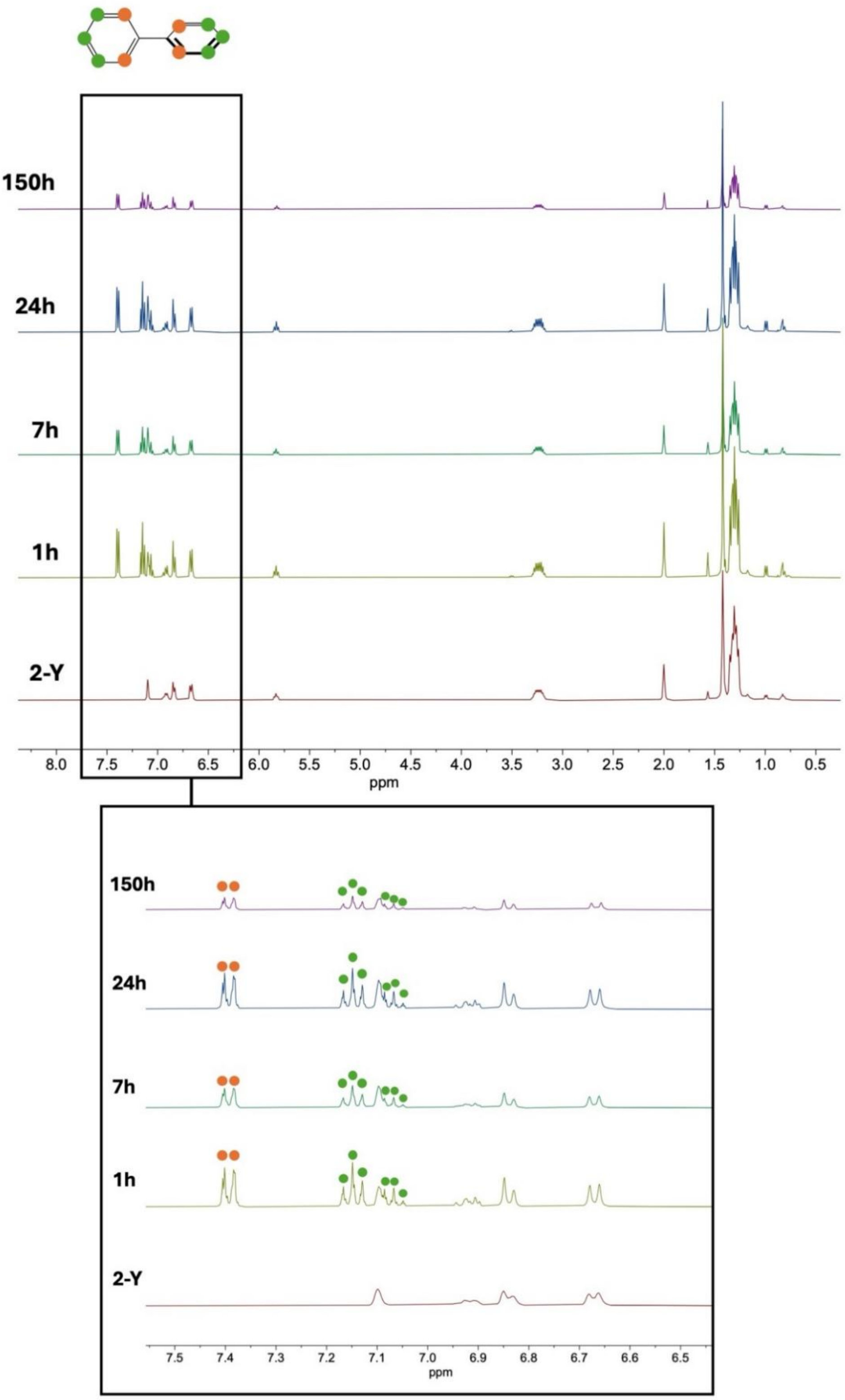

Figure S16 Stacked ^{1}H NMR spectra showing time evolution of a 1 : 1 mixture of **2-Y** and biphenyl in C_6D_6 at 80 °C in the region -3 to 15 ppm, zoomed in 6.5 to 7.5 ppm (bottom).

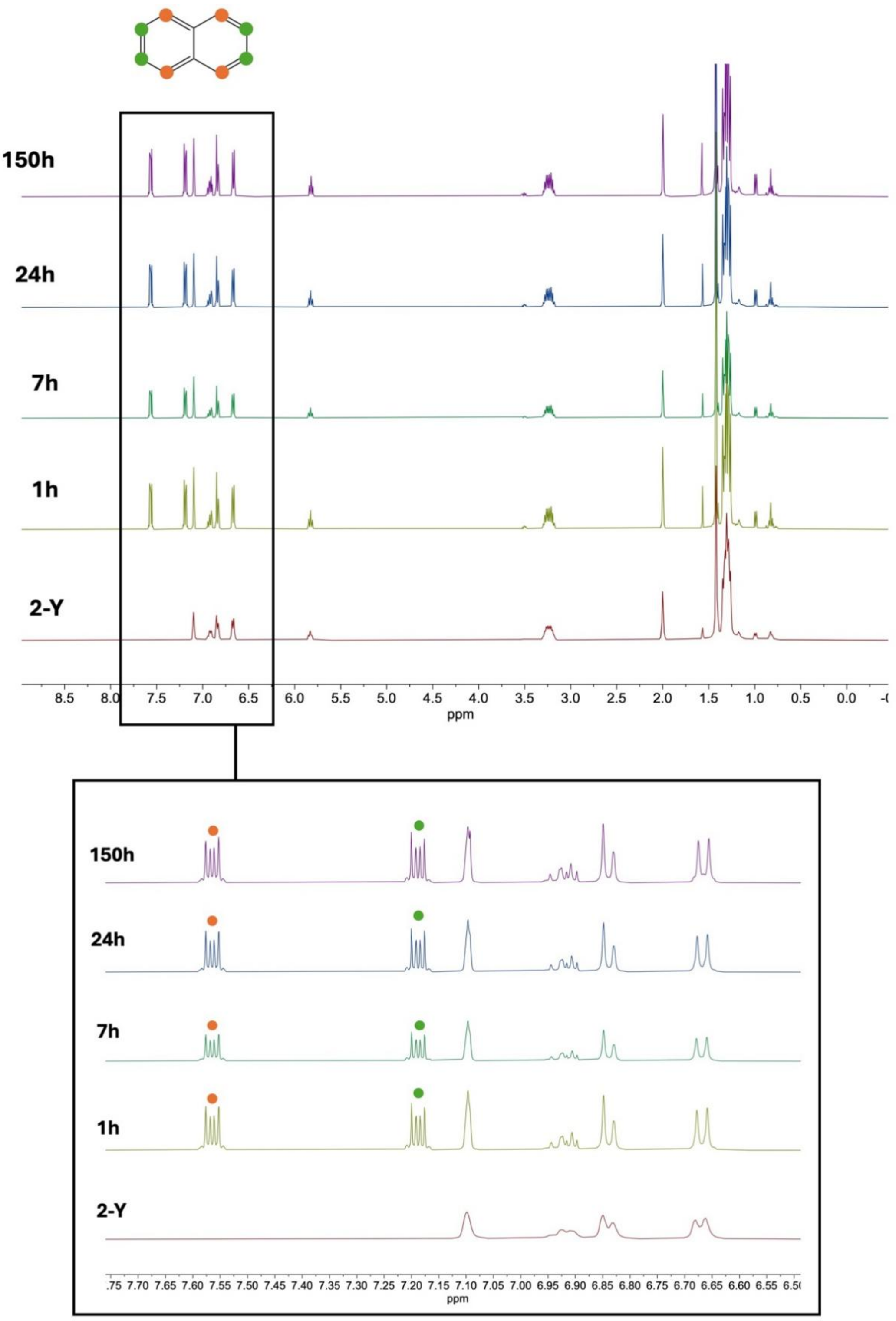

Figure S17 Stacked ^{1}H NMR spectra showing time evolution of a 1 : 1 mixture of **2-Y** and naphthalene in C_6D_6 in the region -3 to 15 ppm, zoomed in 6.5 to 7.8 ppm (bottom).

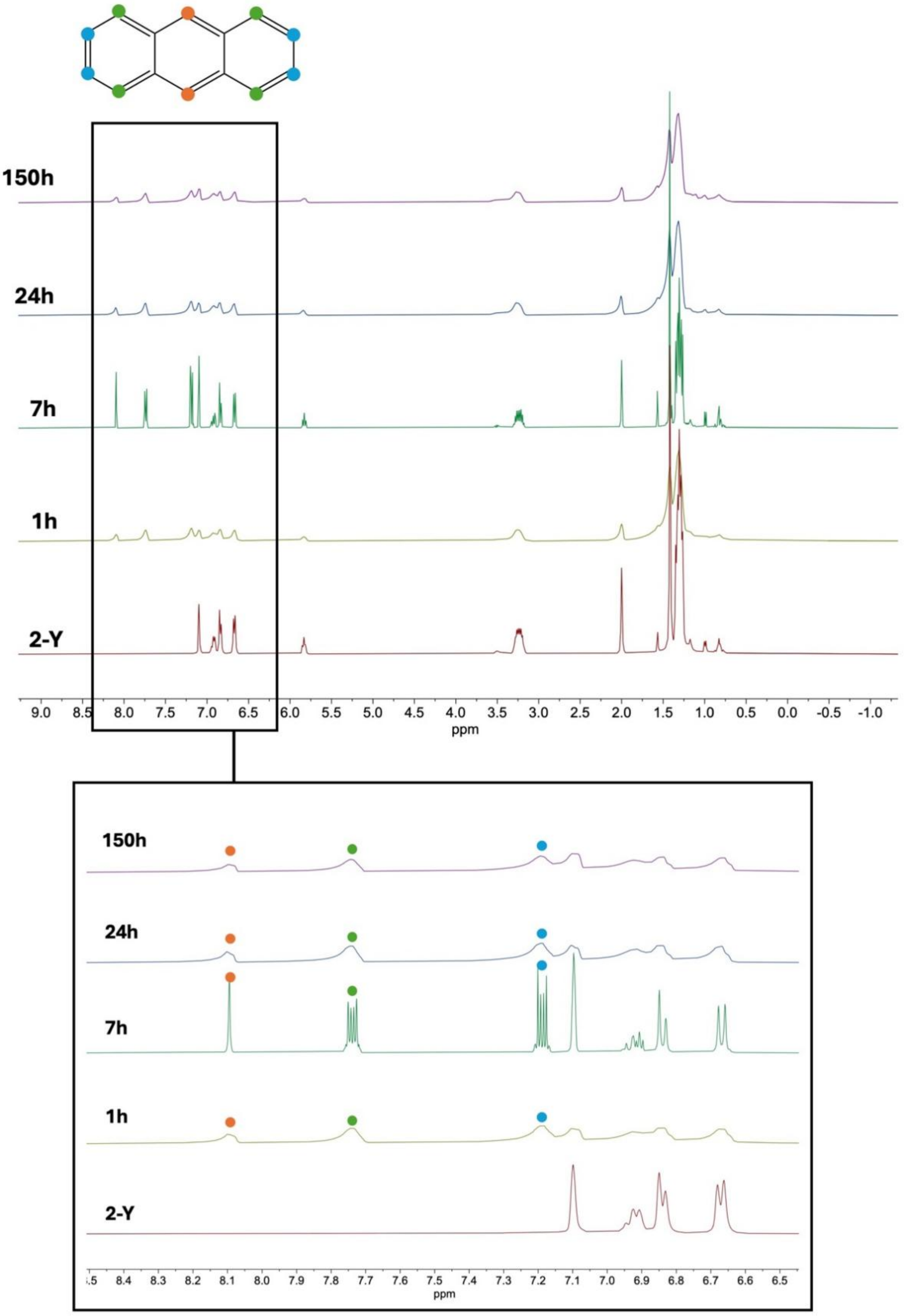

Figure S18 Stacked ^{1}H NMR spectra showing time evolution of a 1 : 1 mixture of **2-Y** and anthracene in C_6D_6 in the region -3 to 15 ppm, zoomed in 6.5 to 8.5 ppm (bottom)..

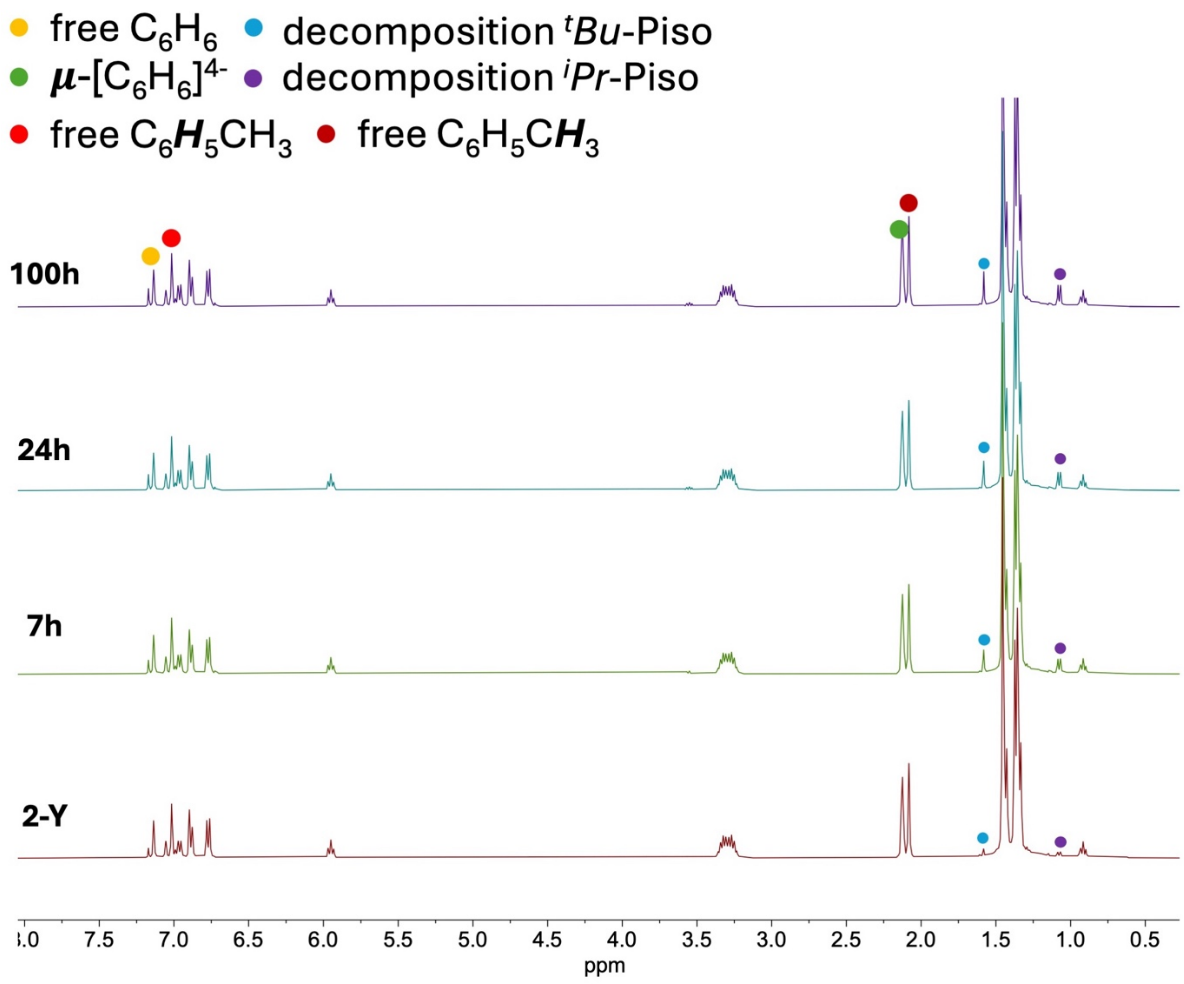

Figure S19 Stacked ^{1}H NMR spectra showing time evolution of a solution of **2-Y** in C_7D_8 heated at 80 °C for up to 100 h in the region 0 to 8 ppm. No arene exchange was observed between **2-Y** and C_7D_8, just some minor decomposition.

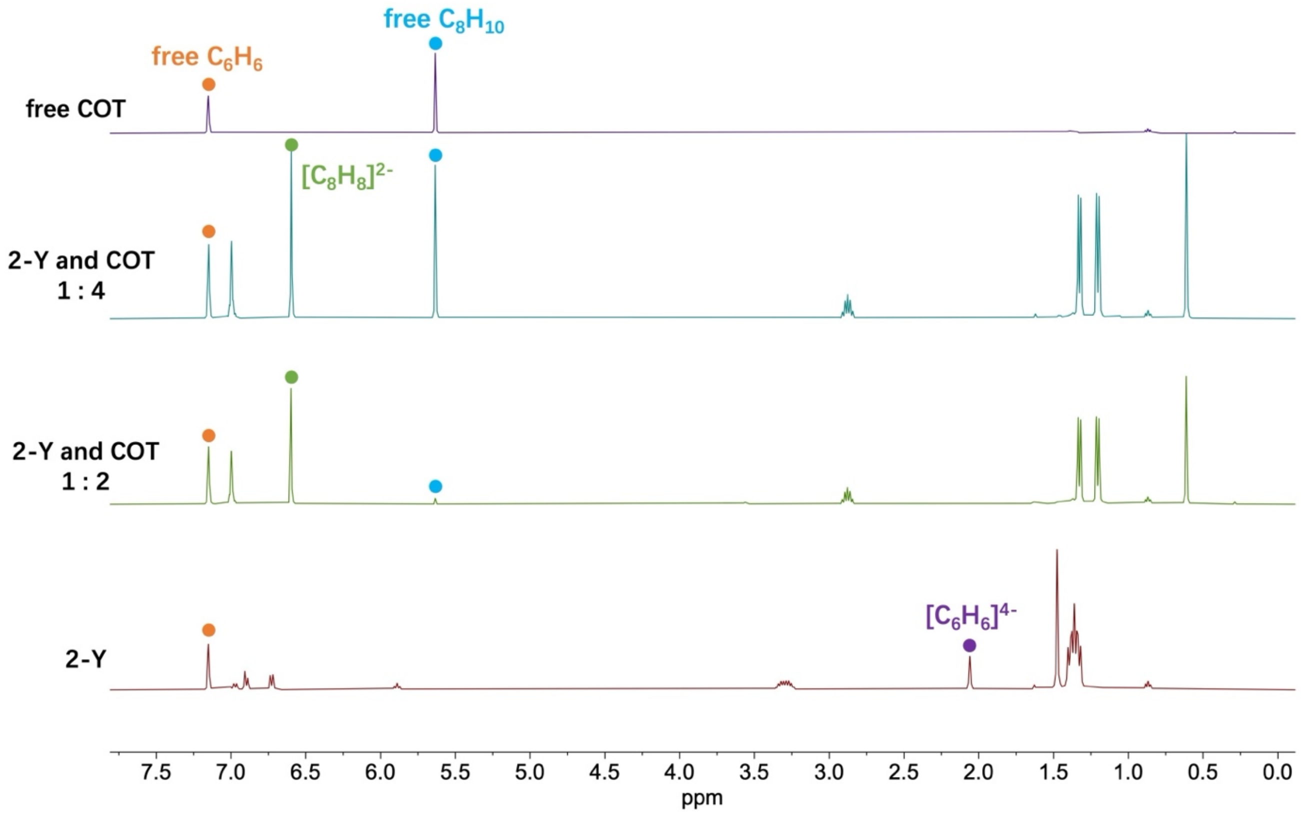

Figure S20 Stacked ^{1}H NMR spectra of the comparison between free COT, 1 : 4 and 1 : 2 equivalent mixtures of **2-Y** and COT, and free **2-Y** in C_6D_6 (from top to bottom) in the region 0 to 7.5 ppm.

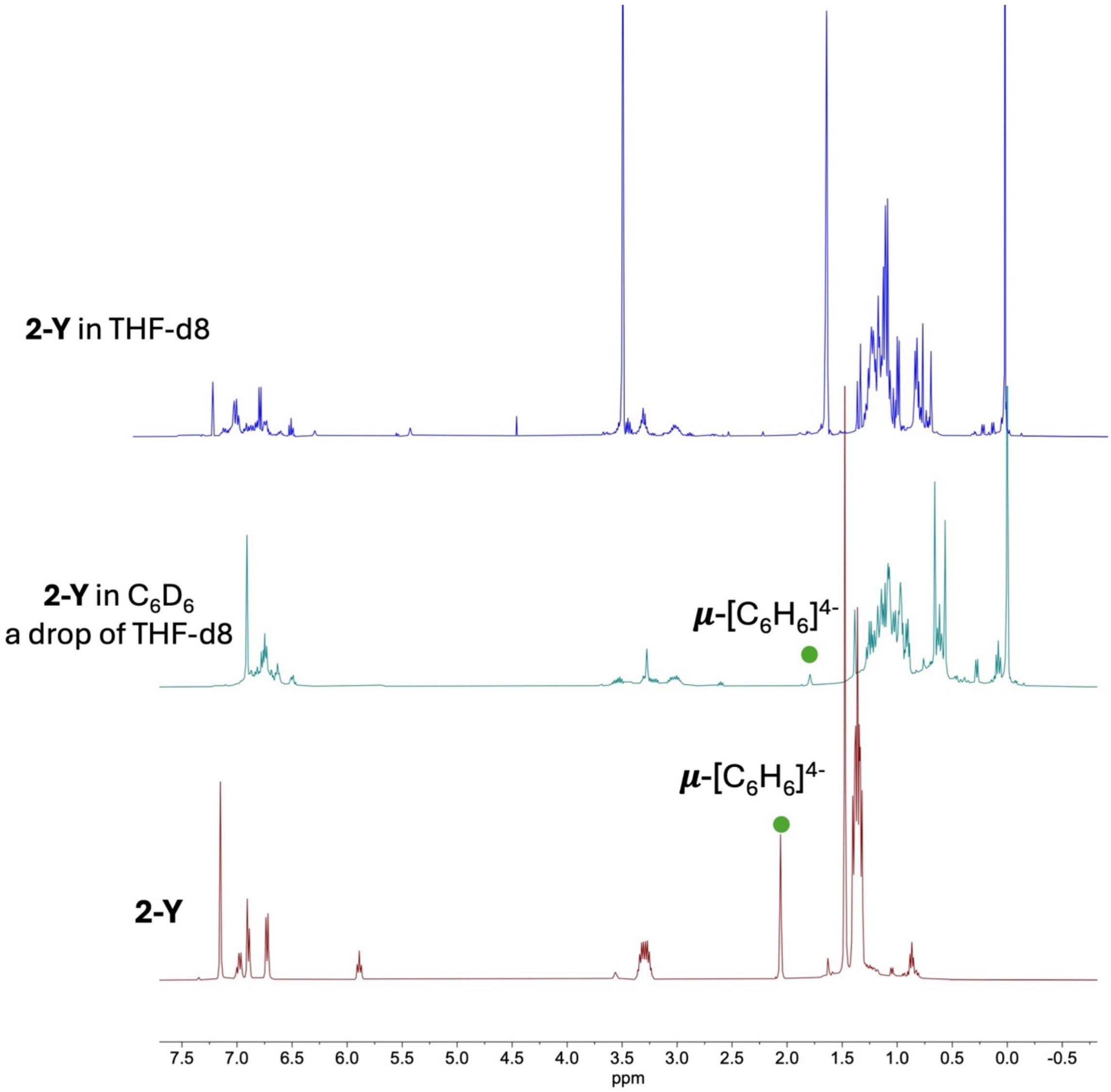

Figure S21 Stacked ^{1}H NMR spectra of the comparison between the reactions of **2-Y** and a drop of d_8-THF in C_6D_6 or neat d_8-THF in the region 0 to 8.0 ppm.

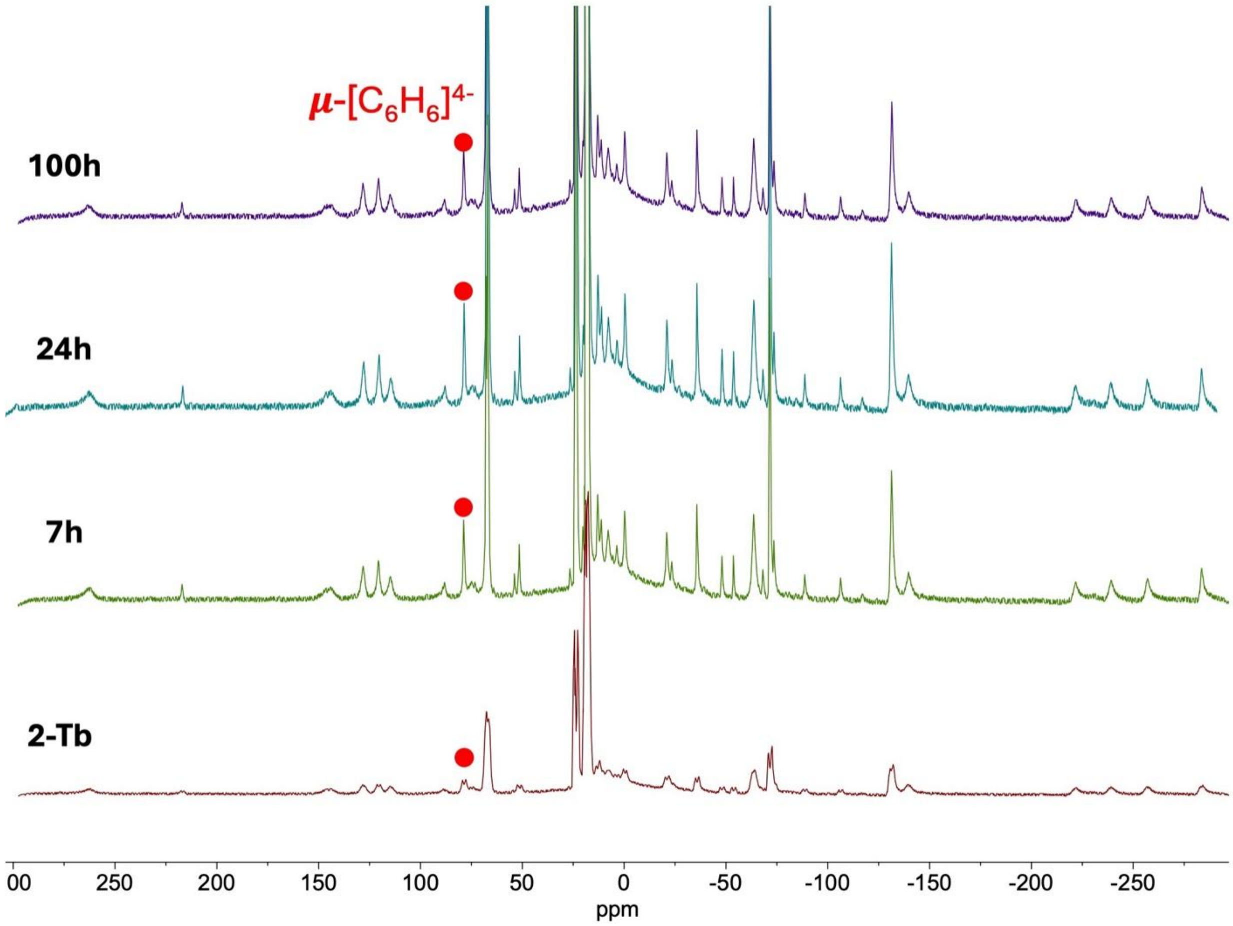

Figure S22 Stacked ^{1}H NMR spectra showing time evolution of a solution of **2-Tb** in C_7D_8 heated at 80 °C in the region -300 to 300 ppm. No arene exchange was observed between **2-Tb** and C_7D_8.

2. Infrared spectra

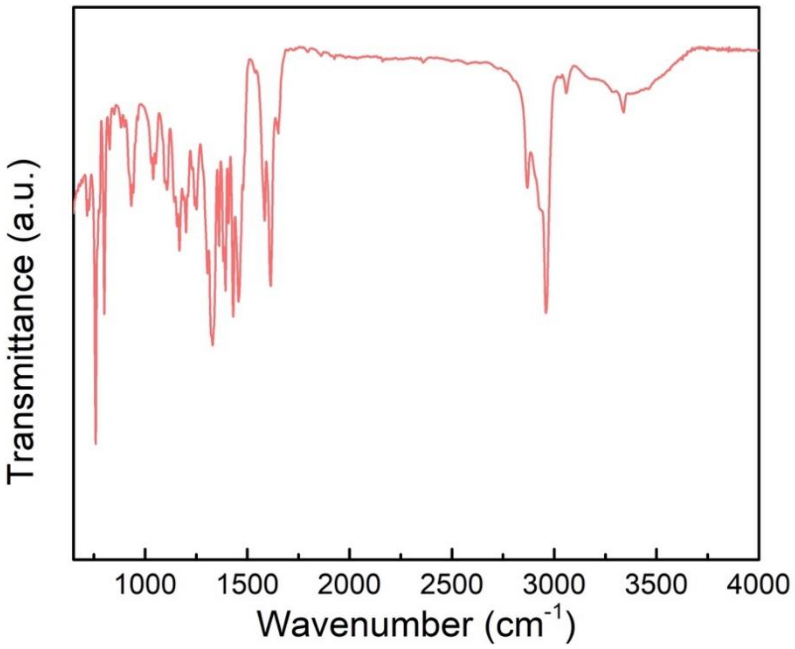

Figure S23 ATR-IR spectrum of **1-Gd-I**.

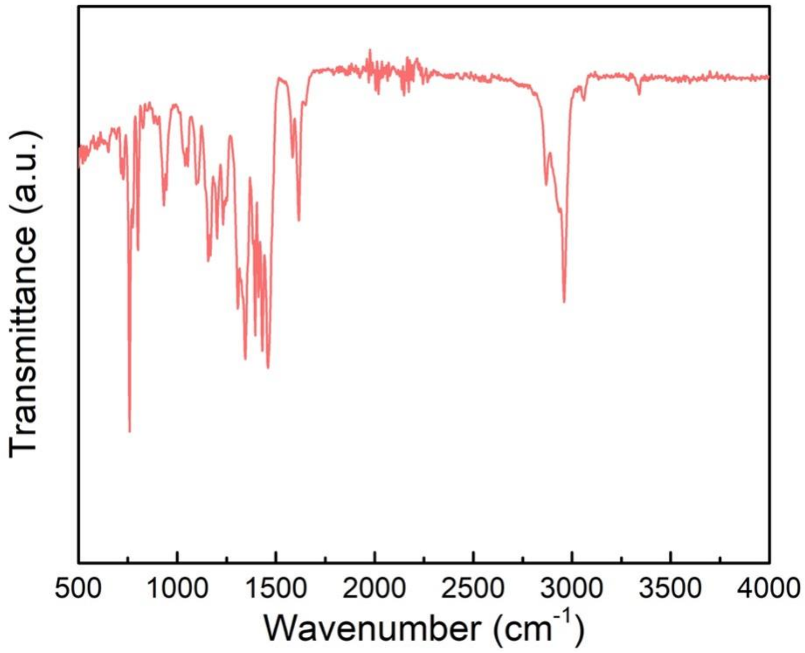

Figure S24 ATR-IR spectrum of **1-Tb-Cl**.

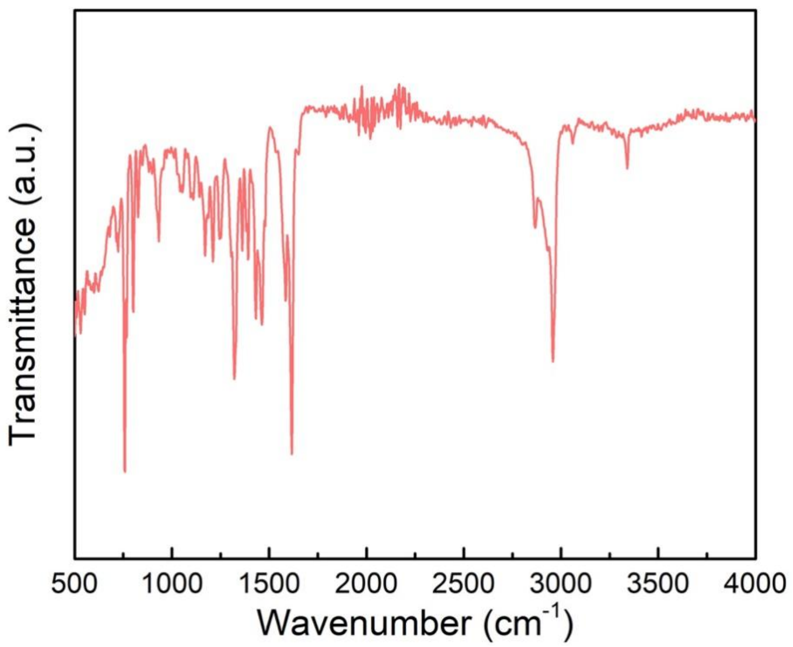

Figure S25 ATR-IR spectrum of **1-Dy-Cl**.

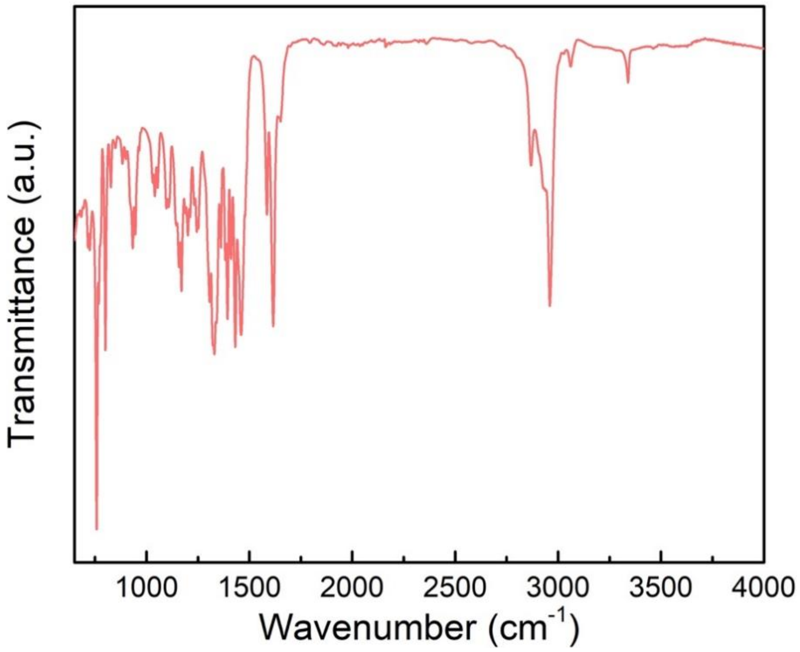

Figure S26 ATR-IR spectrum of **1-Y-I**.

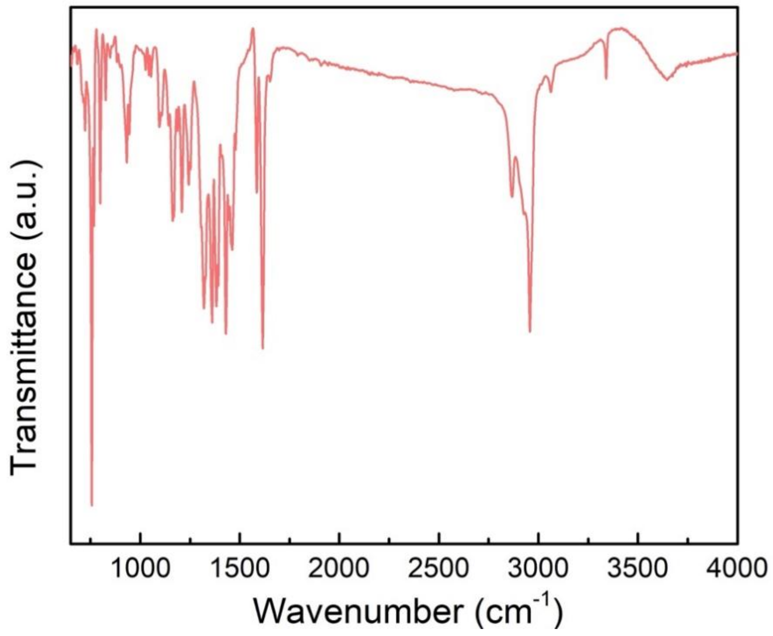

Figure S27 ATR-IR spectrum of **2-Gd**.

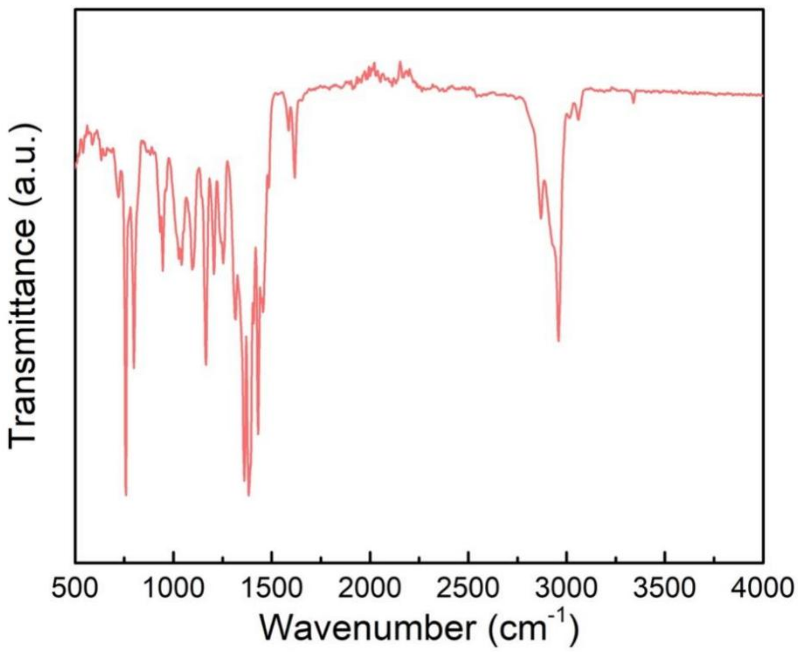

Figure S28 ATR-IR spectrum of **2-Tb**.

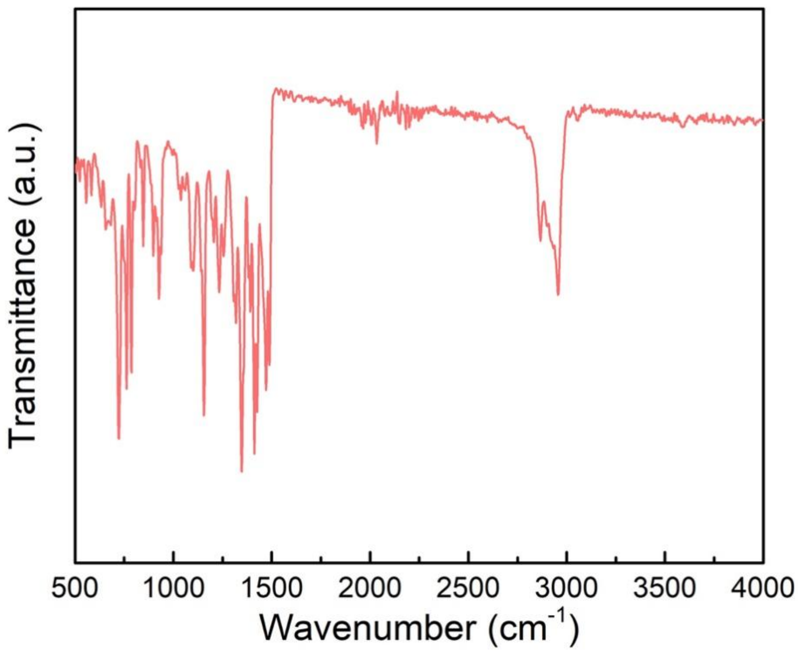

Figure S29 ATR-IR spectrum of **2-Dy**.

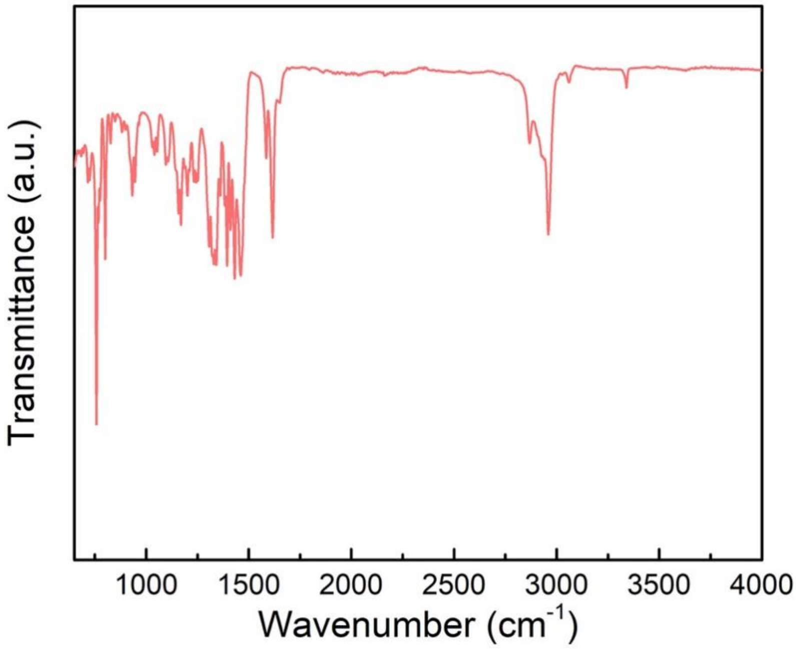

Figure S30 ATR-IR spectrum of **2-Y**.

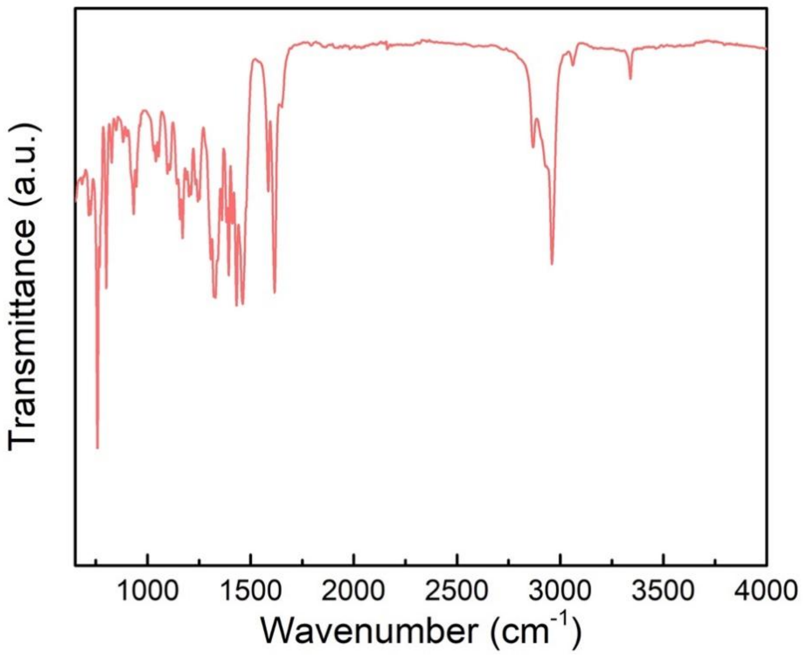

Figure S31 ATR-IR spectrum of **3-Tb**.

3. UV-vis-NIR Spectroscopy

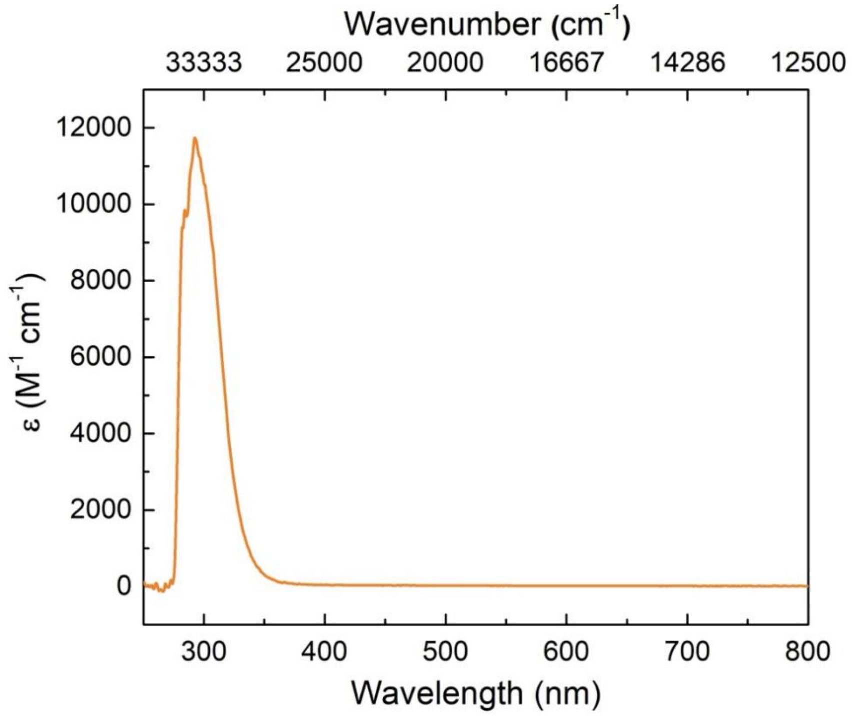

Figure S32 UV-Vis-NIR spectrum of **1-Gd-I** in benzene 0.2 mM.

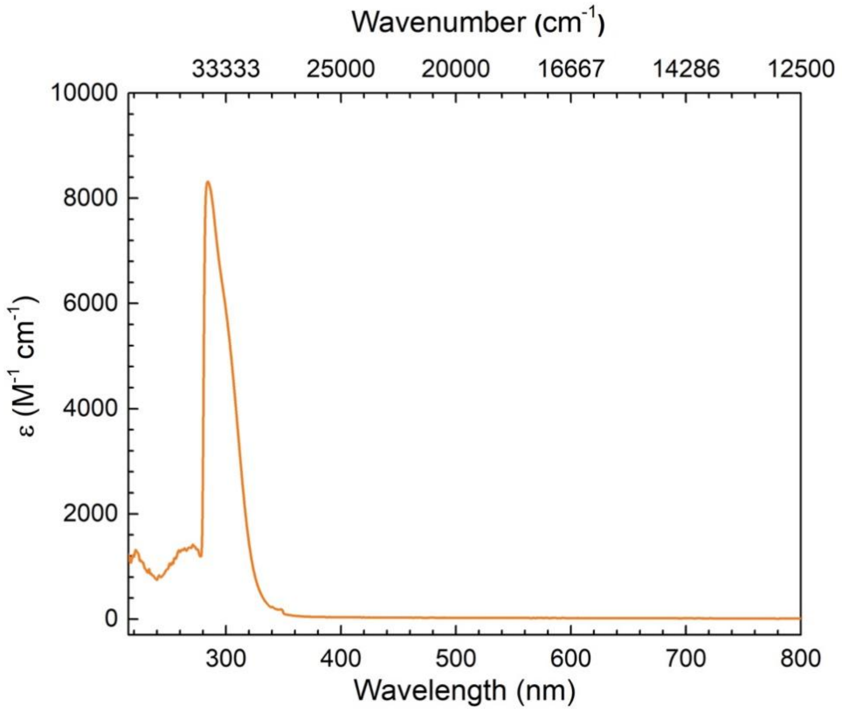

Figure S33 UV-Vis-NIR spectrum of **1-Tb-Cl** in benzene 0.2 mM.

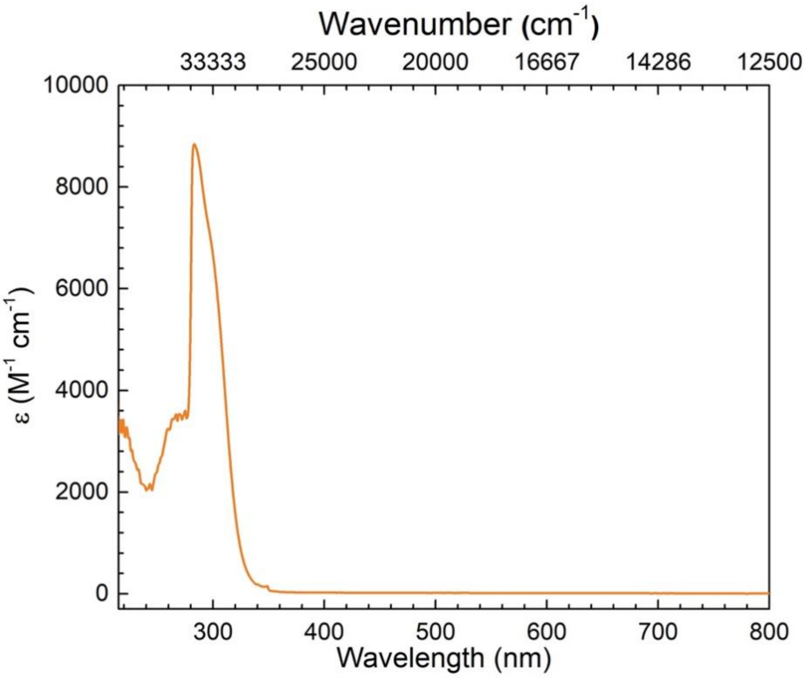

Figure S34 UV-Vis-NIR spectrum of **1-Dy-Cl** in benzene 0.2 mM.

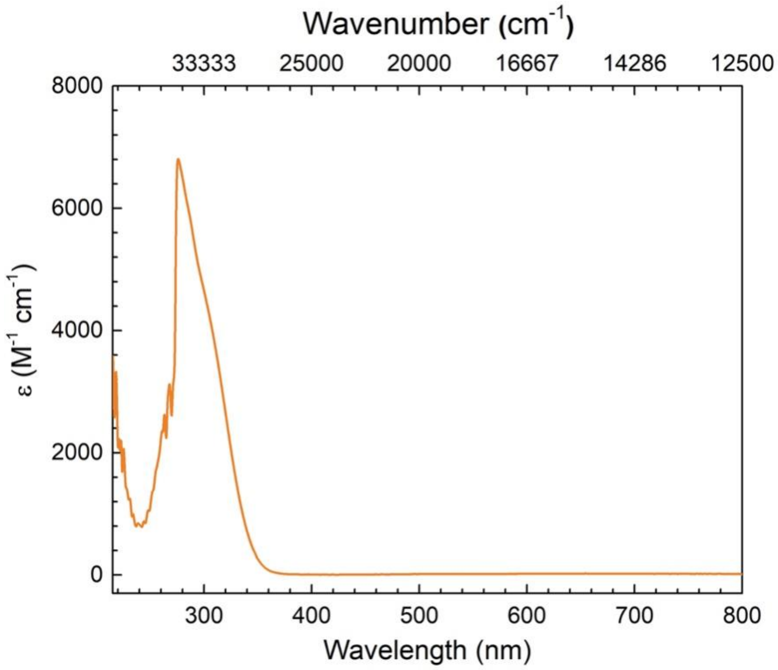

Figure S35 UV-Vis-NIR spectrum of **1-Y-I** in benzene 0.23 mM.

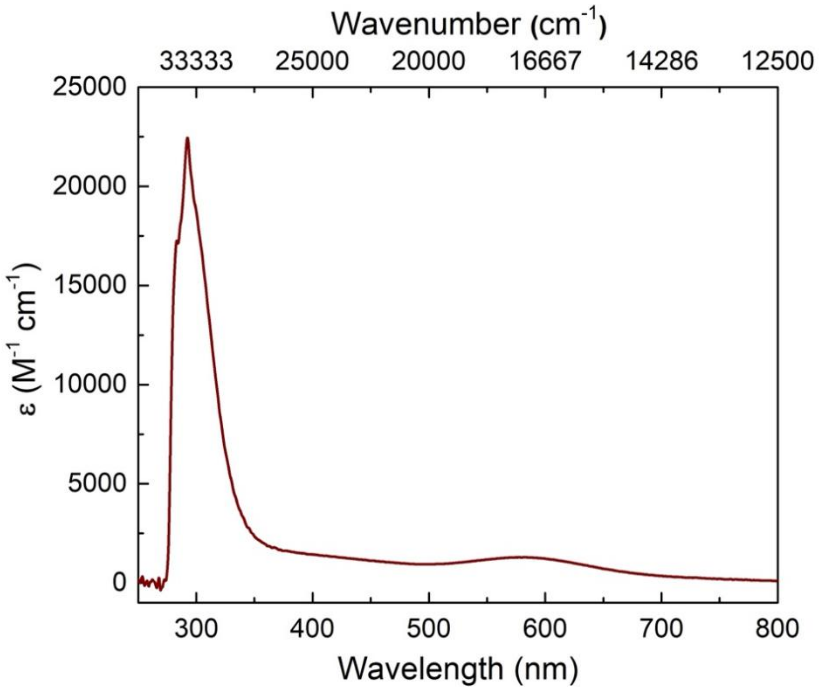

Figure S36 UV-Vis-NIR spectrum of **2-Gd** in benzene 0.08 mM.

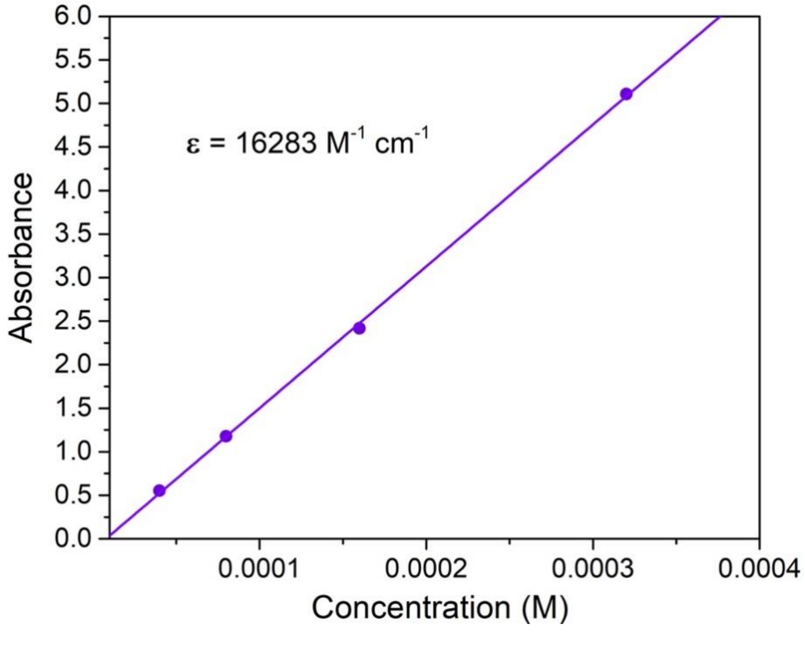

Figure S37 Plot of absorbance versus concentration for the feature at 281 nm in the UV-Vis-NIR spectra of **2-Tb** in benzene. Purple points represent experimental data and the purple line represents the fit to the data used to extract the extinction coefficient.

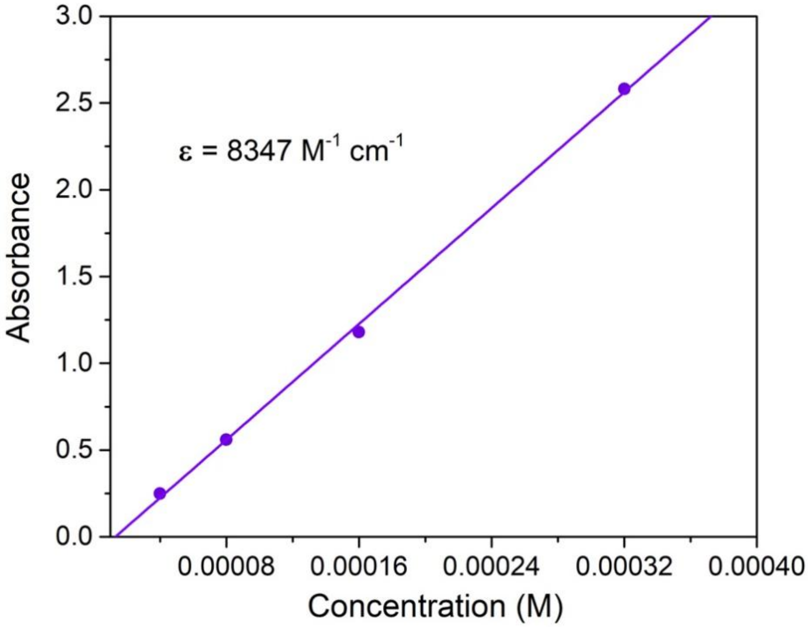

Figure S38 Plot of absorbance versus concentration for the feature at 582 nm in the UV-Vis-NIR spectra of **2-Tb** in benzene. Purple points represent experimental data and the purple line represents the fit to the data used to extract the extinction coefficient.

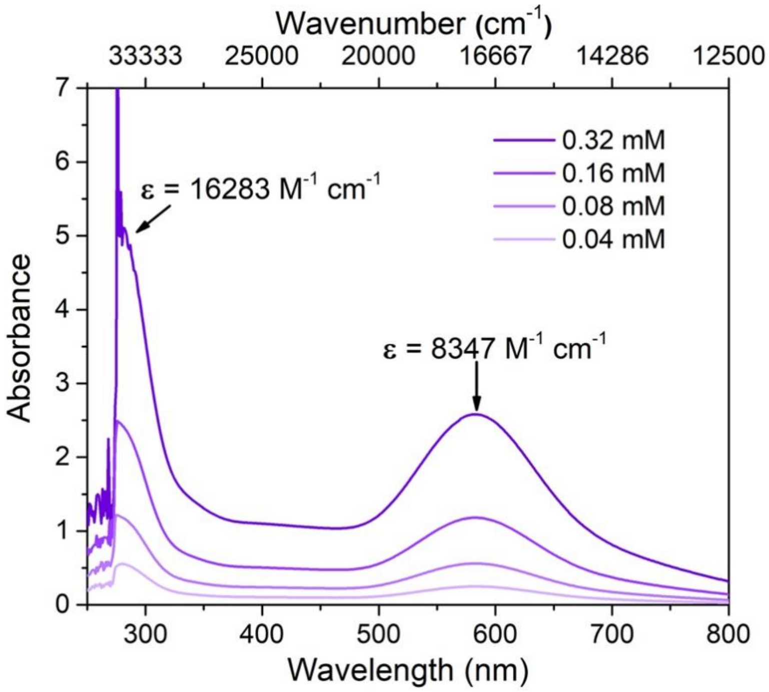

Figure S39 UV-Vis-NIR spectra of **2-Tb** in benzene with varied concentration.

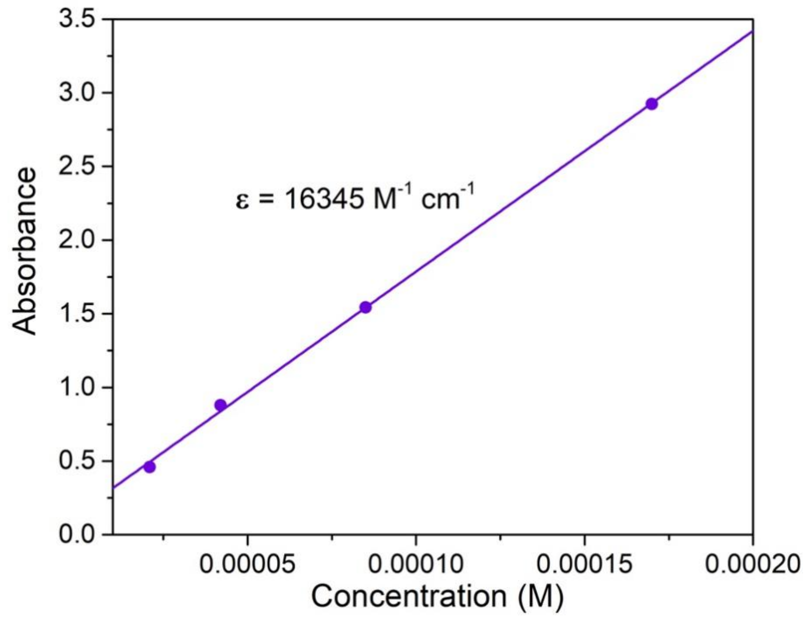

Figure S40 Plot of absorbance versus concentration for the feature at 277 nm in the UV-Vis-NIR spectra of **2-Tb** in hexane. Purple points represent experimental data and the purple line represents the fit to the data used to extract the extinction coefficient.

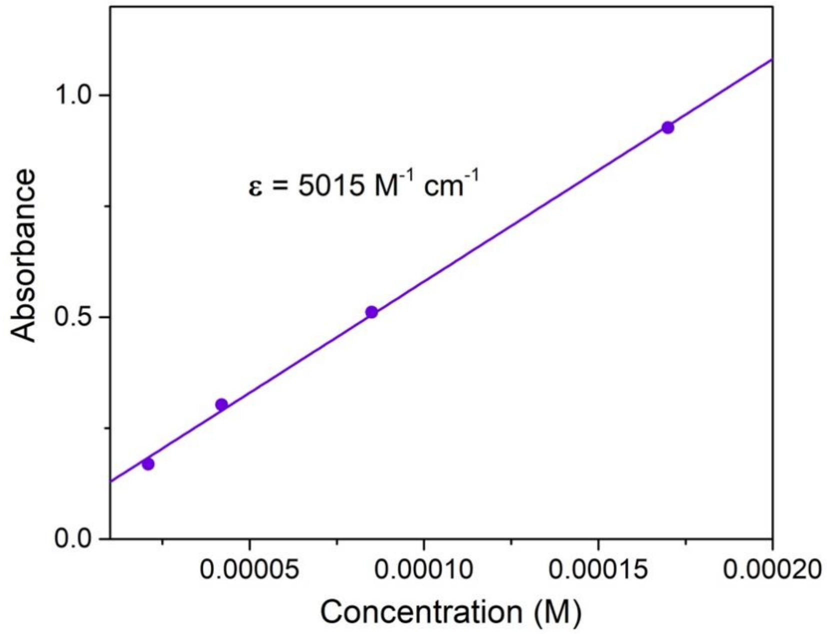

Figure S41 Plot of absorbance versus concentration for the feature at 578 nm in the UV-Vis-NIR spectra of **2-Tb** in hexane. Purple points represent experimental data and the purple line represents the fit to the data used to extract the extinction coefficient.

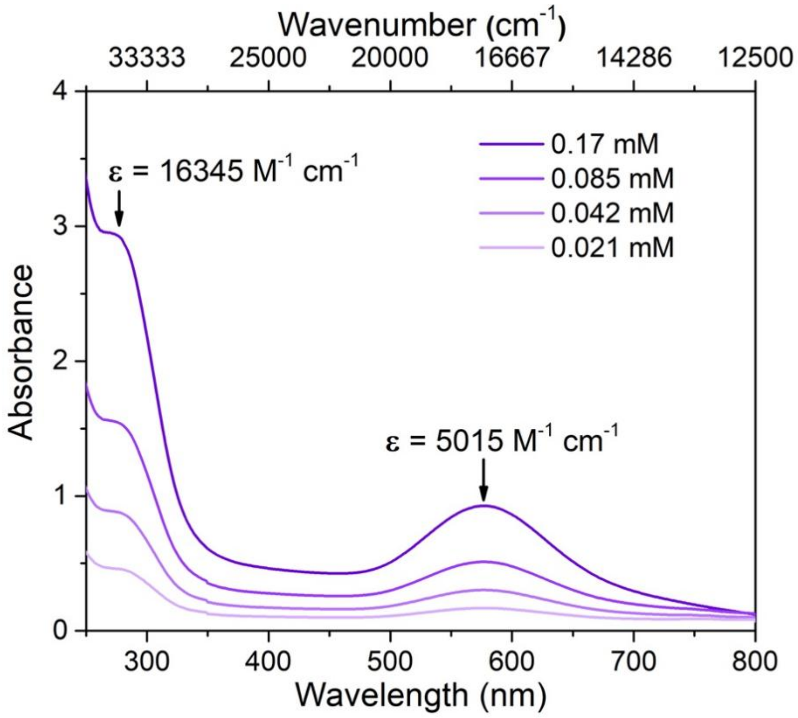

Figure S42 UV-Vis-NIR spectra of **2-Tb** in hexane with varied concentration.

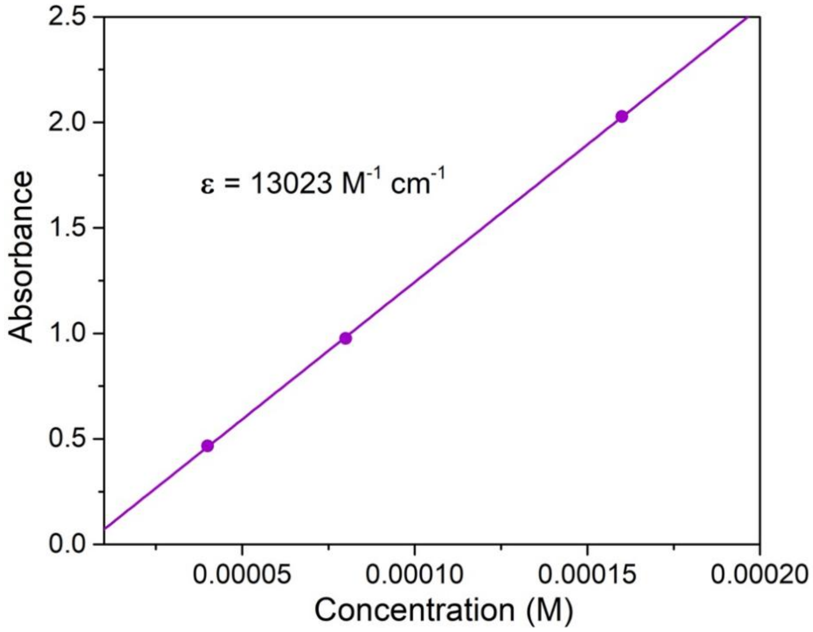

Figure S43 Plot of absorbance versus concentration for the feature at 279 nm in the UV-Vis-NIR spectra of **2-Dy** in benzene. Purple points represent experimental data and the purple line represents the fit to the data used to extract the extinction coefficient.

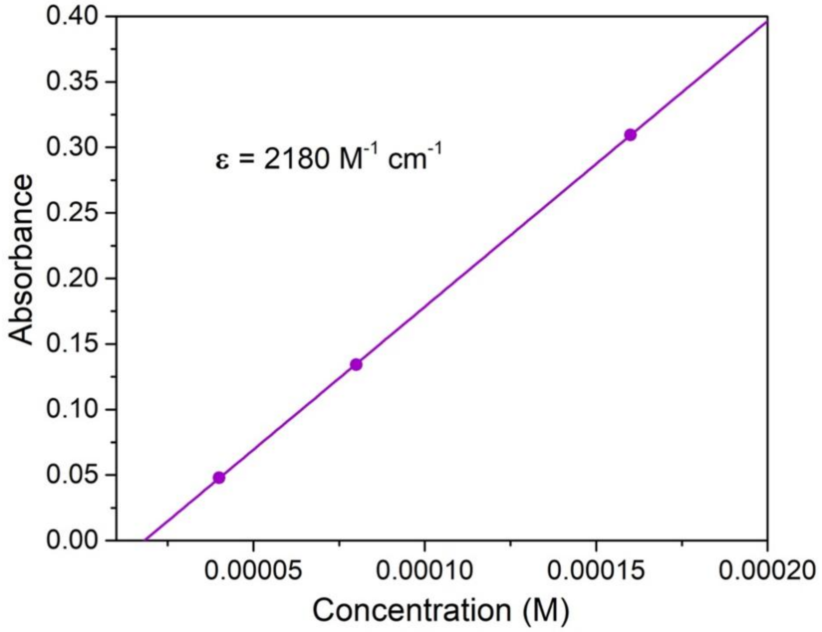

Figure S44 Plot of absorbance versus concentration for the feature at 572 nm in the UV-Vis-NIR spectra of **2-Dy** in benzene. Purple points represent experimental data and the purple line represents the fit to the data used to extract the extinction coefficient.

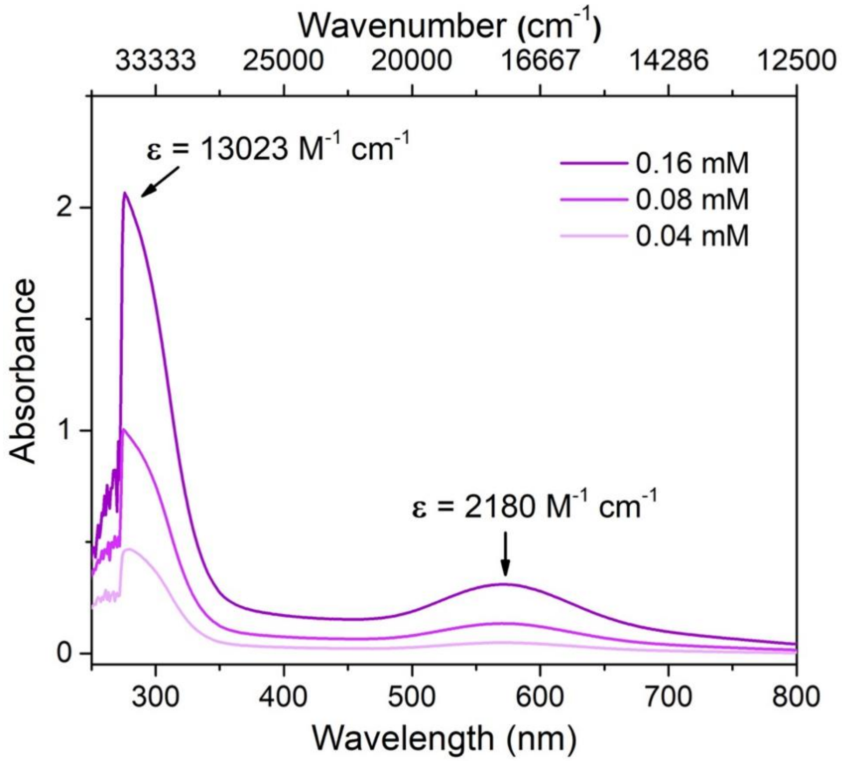

Figure S45 UV-Vis-NIR spectra of **2-Dy** in benzene with varied concentration.

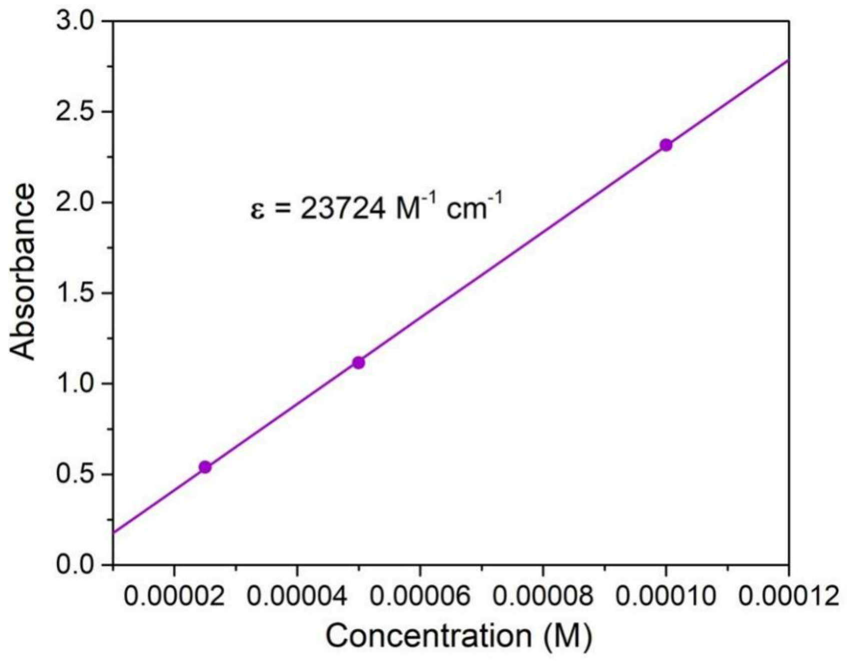

Figure S46 Plot of absorbance versus concentration for the feature at 269 nm in the UV-Vis-NIR spectra of **2-Dy** in hexane. Violet points represent experimental data and the violet line represents the fit to the data used to extract the extinction coefficient.

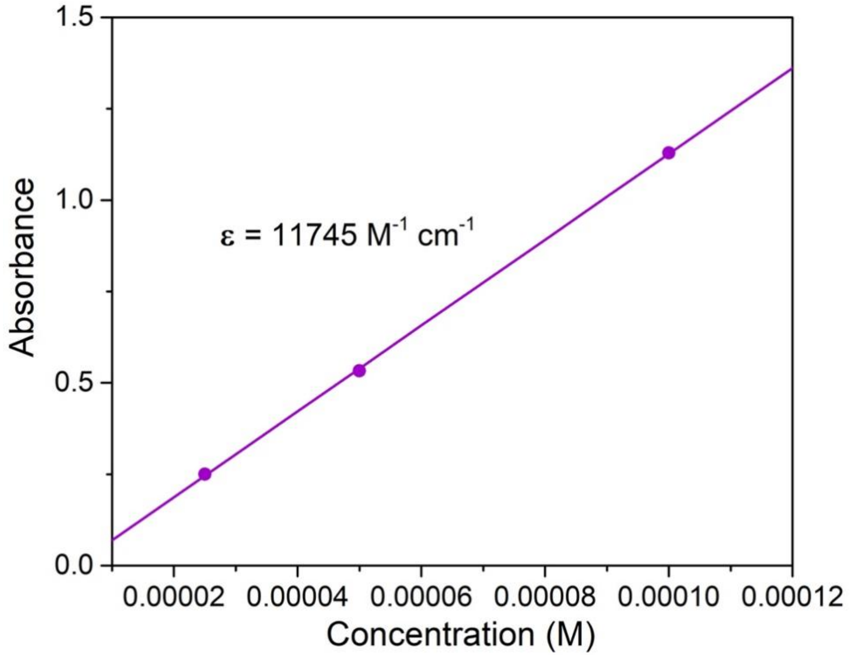

Figure S47 Plot of absorbance versus concentration for the feature at 566 nm in the UV-Vis-NIR spectra of **2-Dy** in hexane. Violet points represent experimental data and the violet line represents the fit to the data used to extract the extinction coefficient.

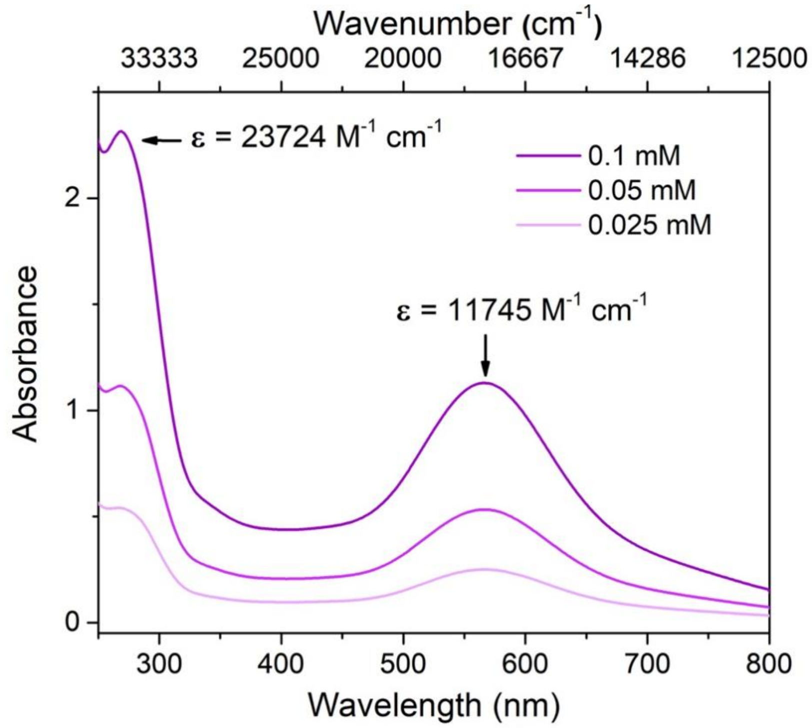

Figure S48 UV-Vis-NIR spectra of **2-Dy** in hexane with varied concentration.

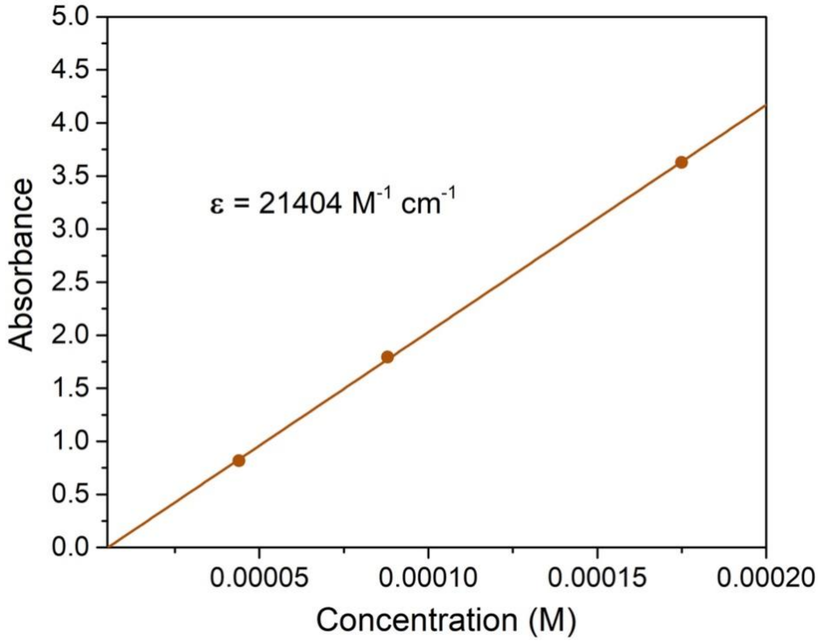

Figure S49 Plot of absorbance versus concentration for the feature at 278 nm in the UV-Vis-NIR spectra of **2-Y** in benzene. brown points represent experimental data and the brown line represents the fit to the data used to extract the extinction coefficient.

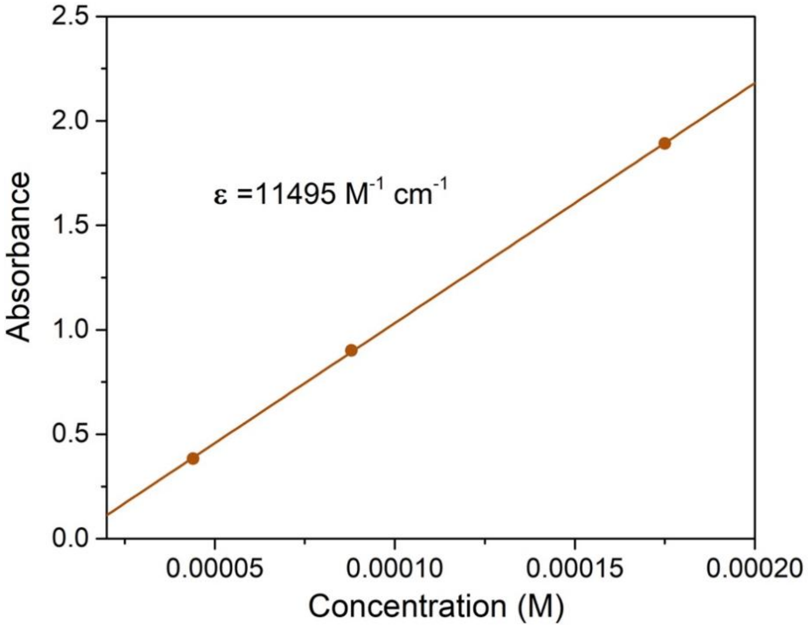

Figure S50 Plot of absorbance versus concentration for the feature at 583 nm in the UV-Vis-NIR spectra of **2-Y** in benzene. brown points represent experimental data and the brown line represents the fit to the data used to extract the extinction coefficient.

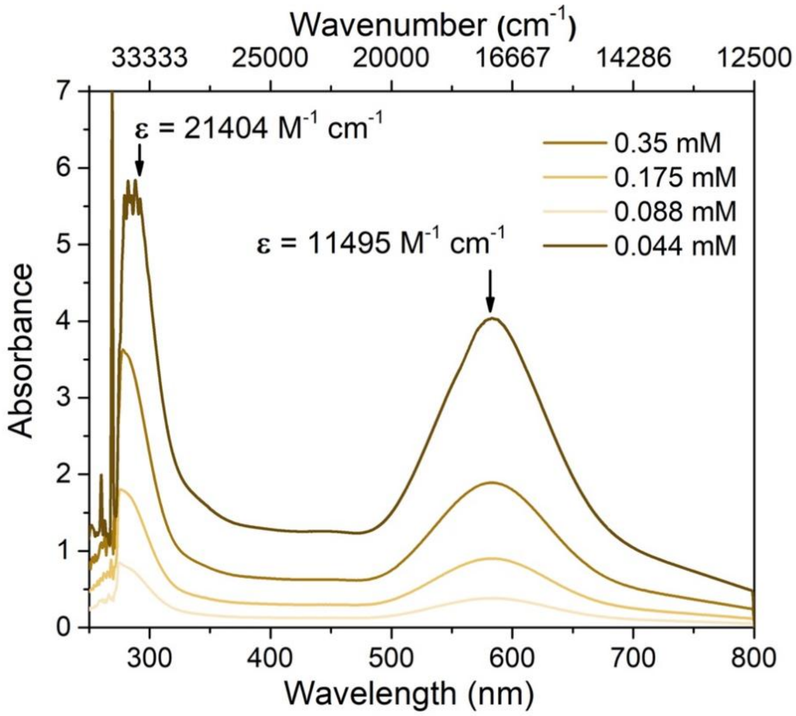

Figure S51 UV-Vis-NIR spectra of **2-Y** in benzene with varied concentration.

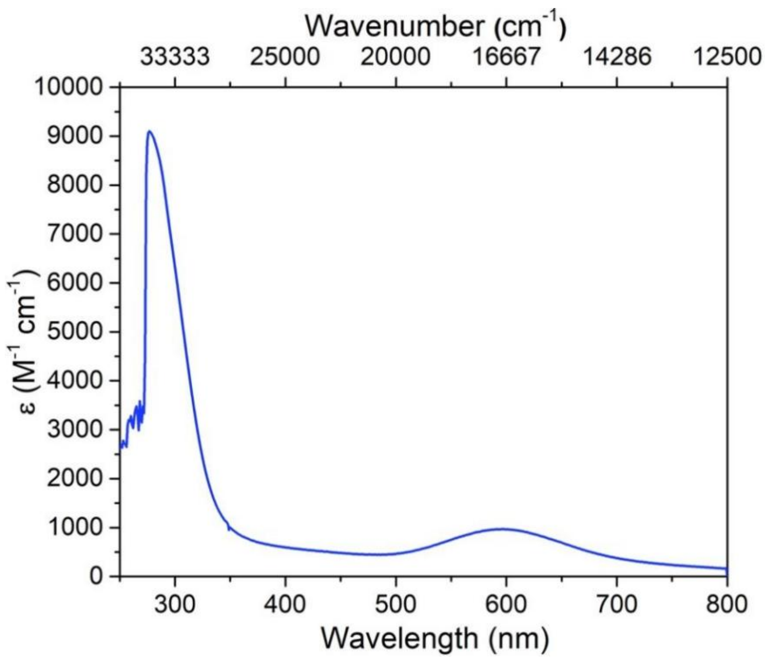

Figure S52 UV-Vis-NIR spectra of **3-Tb** in benzene 0.14 mM.

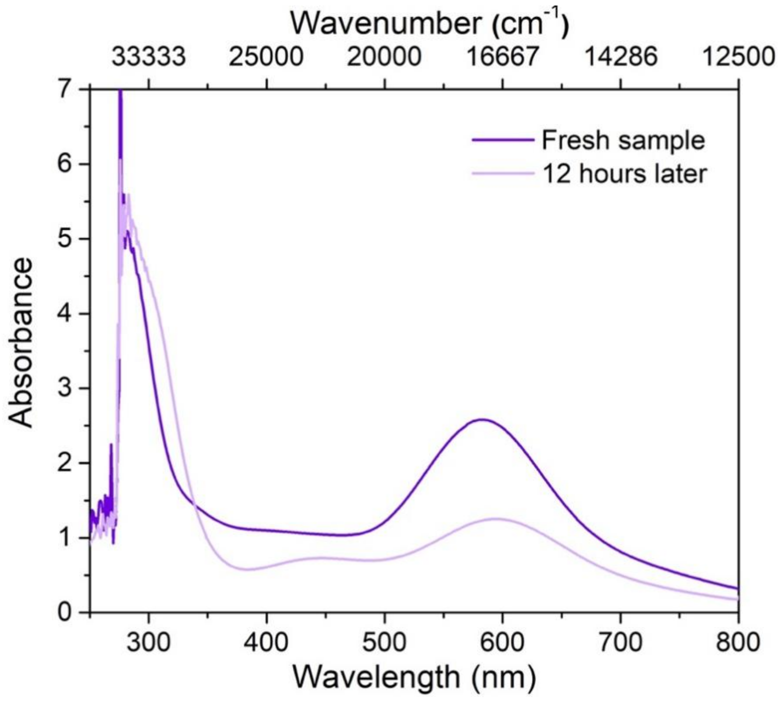

Figure S53 UV-Vis-NIR spectra to compare a fresh solution of **2-Tb** in benzene at room temperature, and aged samples after 12 hour at 80 °C.

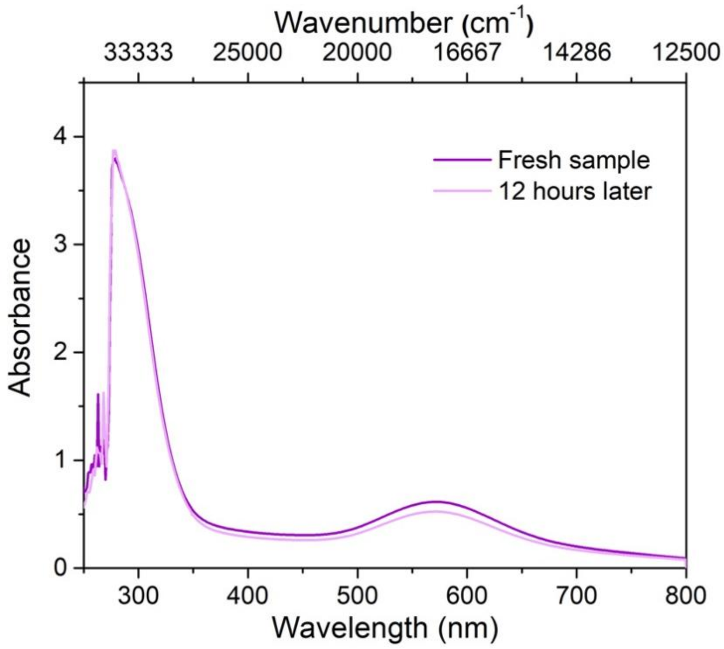

Figure S54 UV-Vis-NIR spectra to compare a fresh solution of **2-Dy** in benzene at room temperature, and aged samples after 12 hour at 80 °C.

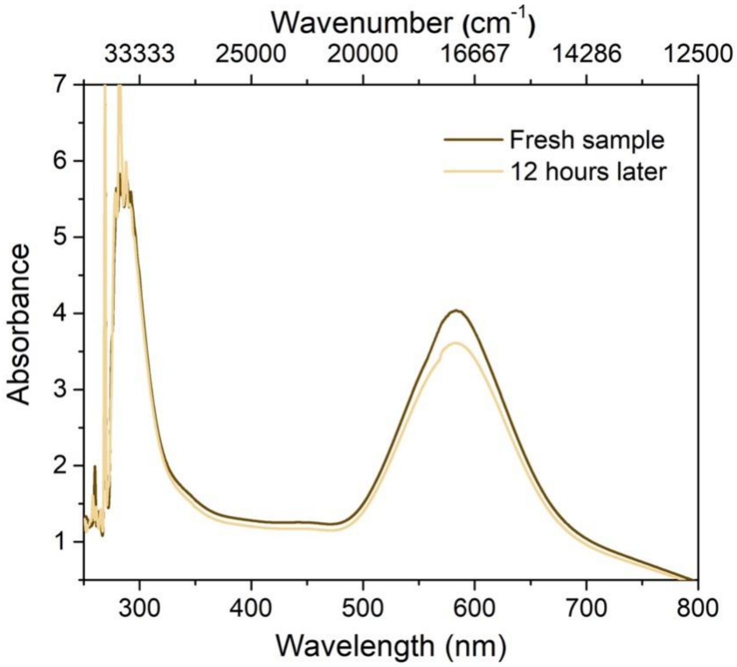

Figure S55 UV-Vis-NIR spectra to compare a fresh solution of **2-Y** in benzene at room temperature, and aged samples after 12 hour at 80 °C.

4. Crystallography

Table S1 Selected crystallographic data for **1-Gd-I**, **1-Tb-Cl** and **1-Dy-Cl**.

**Compounds**	**1-Gd-I**	**1-Tb-Cl**	**1-Dy-Cl**
Formula	$C_{65}H_{94}GdIN_4$	$C_{58}H_{86}ClN_4Tb$	$C_{58}H_{86}ClN_4Dy$
M, g mol^{-1}	1215.59	1033.67	1037.24
Temperature, K	150.00(10)	100.15(10)	100.01(10)
Space group	*C*2/*c*	*C*2/*c*	*C*2/*c*
a, Å	26.171(2)	23.9599(8)	23.9612(13)
b, Å	11.0443(10)	10.5837(2)	10.5893(4)
c, Å	23.940(3)	22.4979(8)	22.5423(13)
α, deg	90	90	90
β, deg	119.762(3)	112.183(4)	112.235(6)
γ, deg	90	90	90
V, Å^3	6007.0(11)	5282.8(3)	5294.4(5)
Z	4	4	4
d_{cal}, g cm^{-3}	1.344	1.300	1.301
Radiation	MoKα (λ = 0.71073)	MoKα (λ = 0.71073)	MoKα (λ = 0.71073)
2θ range, deg	4.1 to 50.706	6.722 to 58.088	6.722 to 58.328
R_{int}	0.0762	0.0389	0.0534
Final indexes [F^2>$2\sigma(F^2)$]	R_1 = 0.0254, wR_2 = 0.0584	R_1 = 0.0400, wR_2 = 0.0735	R_1 = 0.0498, wR_2 = 0.1084
R indexes (all data)	R_1 = 0.0288, wR_2 = 0.0607	R_1 = 0.0480, wR_2 = 0.0788	R_1 = 0.0604, wR_2 = 0.1165
S^a	1.038	1.045	1.015
Residual map, e Å^{-3}	0.36/-0.30	1.28/-1.01	2.08/-1.55

^{a} Conventional $R = \Sigma||F_o| - |F_c||/\Sigma|F_o|$; $R_w = [\Sigma w(F_o^2 - F_c^2)^2/\Sigma w(F_o^2)^2]^{1/2}$; $S = [\Sigma w(F_o^2 - F_c^2)^2/\text{no. data} - \text{no. params})]^{1/2}$ for all data.

Table S2 Selected crystallographic data for **1-Y-I**, **2-Gd** and **2-Tb**.

**Compounds**	**1-Y-I**	**2-Gd**	**2-Tb**
Formula	$C_{65}H_{94}IN_4Y$	$C_{64}H_{92}Gd_2N_4$	$C_{64}H_{92}N_4Tb_2$
M, g mol^{-1}	1147.28	1231.91	1235.29
Temperature, K	100.0(10)	150.0(7)	99.9(7)
Space group	$I2/a$	$Pbca$	$Pna2_1$
a, Å	25.2282(11)	18.1193(11)	30.89030(15)
b, Å	10.9625(3)	35.666(2)	10.27991(6)
c, Å	23.8393(9)	36.498(3)	18.11204(9)
α, deg	90	90	90
β, deg	116.041(5)	90	90
γ, deg	90	90	90
V, Å^3	5923.8(4)	23587(3)	5751.47(5)
Z	4	16	4
d_{cal}, g cm^{-3}	1.286	1.388	1.429
Radiation	MoKα (λ = 0.71073)	MoKα (λ = 0.71073)	CuKα (λ = 1.54184)
2θ range, deg	6.81 to 58.298	4.052 to 50.702	5.722 to 155.214
R_{int}	0.0274	0.0795	0.0475
Final indexes [F^2>$2\sigma(F^2)$]	R_1 = 0.0343, wR_2 = 0.0772	R_1 = 0.0261, wR_2 = 0.0554	R_1 = 0.0329, wR_2 = 0.0895
R indexes (all data)	R_1 = 0.0477, wR_2 = 0.0852	R_1 = 0.0361, wR_2 = 0.0604	R_1 = 0.0347, wR_2 = 0.0903
S^a	0.923	1.039	1.034
Residual map, e Å^{-3}	0.57/-0.71	1.93/-0.46	1.22/-0.97

^{a} Conventional $R = \Sigma||F_o| - |F_c||/\Sigma|F_o|$; $R_w = [\Sigma w(F_o^2 - F_c^2)^2/\Sigma w(F_o^2)^2]^{1/2}$; $S = [\Sigma w(F_o^2 - F_c^2)^2/\text{no. data} - \text{no. params})]^{1/2}$ for all data.

Table S3 Selected crystallographic data for **2-Dy**, **2-Y** and **3-Tb**.

Compounds	2-Dy	2-Y	3-Tb
Formula	$C_{67}H_{99}N_4Dy_2$	$C_{67}H_{99}N_4Y_2$	$C_{74}H_{118}N_4Tb_2$
M, g mol^{-1}	1285.53	1138.34	1381.60
Temperature, K	100.0(7)	100.0(4)	104(6)
Space group	$P2_1/c$	$P2_1/c$	P-1
a, Å	10.83820(10)	10.83980(10)	14.4004(3)
b, Å	38.9439(5)	38.9679(2)	15.4059(3)
c, Å	15.0078(3)	15.00510(10)	18.0500(4)
α, deg	90	90	97.598(2)
β, deg	105.063(2)	105.0290(10)	103.094(2)
γ, deg	90	90	112.008(2)
V, Å^3	6116.87(17)	6121.42(8)	3510.01(14)
Z	4	4	2
d_{cal}, g cm^{-3}	1.396	1.234	1.320
Radiation	CuKα (λ = 1.54184)	CuKα (λ = 1.54184)	CuKα (λ = 1.54184)
2θ range, deg	6.508 to 133.192	4.536 to 160.27	6.812 to 58.376
R_{int}	0.0875	0.0466	0.0493
Final indexes [F^2>2σ(F^2)]	R_1 = 0.0823, wR_2 = 0.2226	R_1 = 0.0394, wR_2 = 0.1025	R_1 = 0.0411, wR_2 = 0.1069
R indexes (all data)	R_1 = 0.0855, wR_2 = 0.2244	R_1 = 0.0420, wR_2 = 0.1058	R_1 = 0.0439, wR_2 = 0.1089
S^a	1.134	1.056	1.028
Residual map, e Å^{-3}	4.36/-3.08	2.45/-0.83	2.36/-1.67

^{a} Conventional $R = \Sigma||F_o| - |F_c||/\Sigma|F_o|$; $R_w = [\Sigma w(F_o^2 - F_c^2)^2/\Sigma w(F_o^2)^2]^{1/2}$; $S = [\Sigma w(F_o^2 - F_c^2)^2$/no. data – no. params)]$^{1/2}$ for all data.

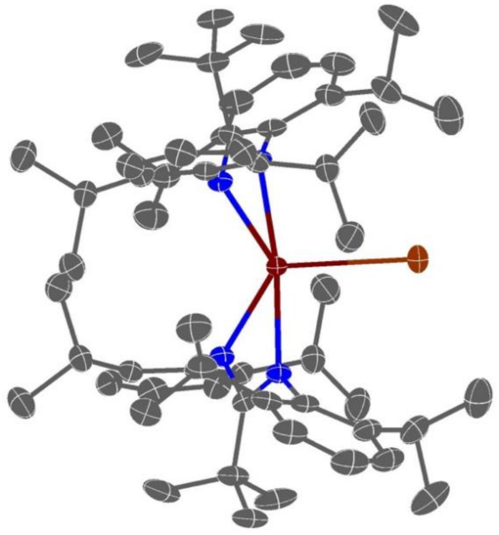

Figure S56 Molecular structure of **1-Gd-I** with selective atom labelling (C = gray, N = blue, I = brown, Gd = wine). Thermal ellipsoids set at 50 % probability level and hydrogen atoms omitted for clarity.

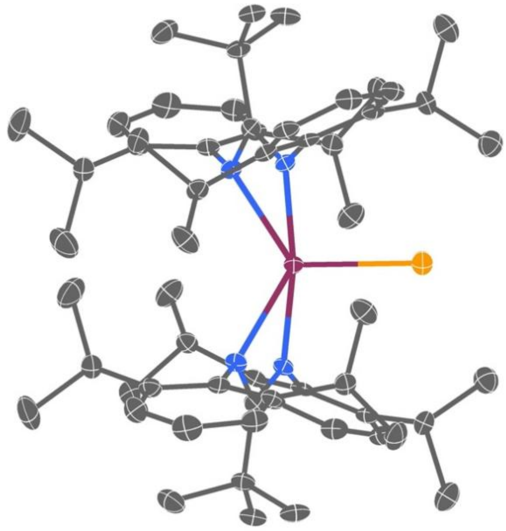

Figure S57 Molecular structure of **1-Dy-Cl** with selective atom labelling (C = gray, N = blue, Cl = yellow, Dy = magenta). Thermal ellipsoids set at 50 % probability level and hydrogen atoms omitted for clarity.

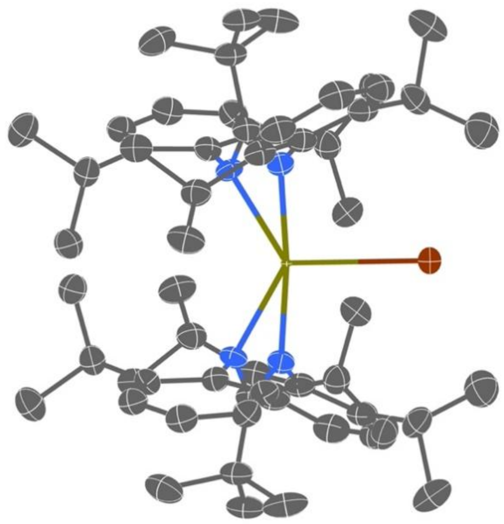

Figure S58 Molecular structure of **1-Y-I** with selective atom labelling (C = gray, N = blue, I = brown, Y = olive). Thermal ellipsoids set at 50 % probability level and hydrogen atoms omitted for clarity.

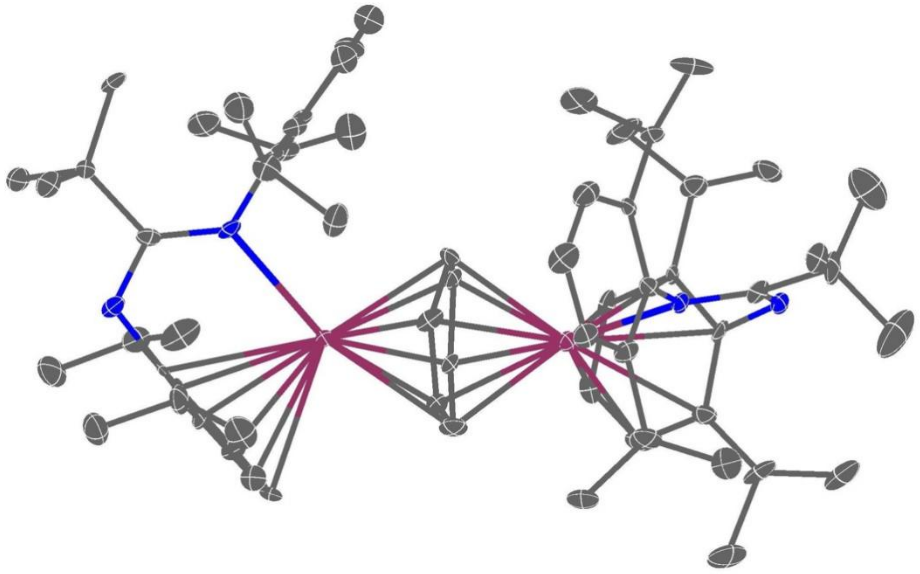

Figure S59 Molecular structure of **2-Dy** with selective atom labelling (C = gray, N = blue, Dy = magenta). Thermal ellipsoids set at 50 % probability level and hydrogen atoms omitted for clarity.

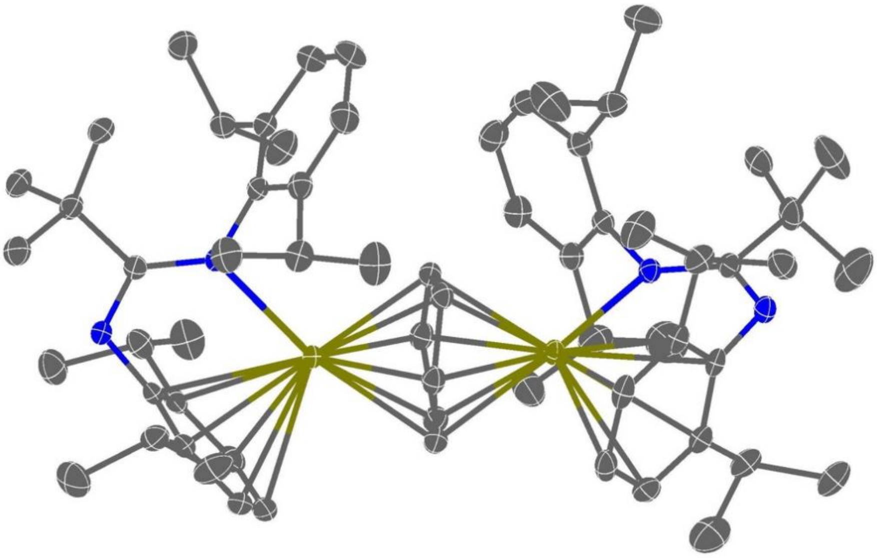

Figure S60 Molecular structure of **2-Y** with selective atom labelling (C = gray, N = blue, Y = olive). Thermal ellipsoids set at 50 % probability level and hydrogen atoms omitted for clarity.

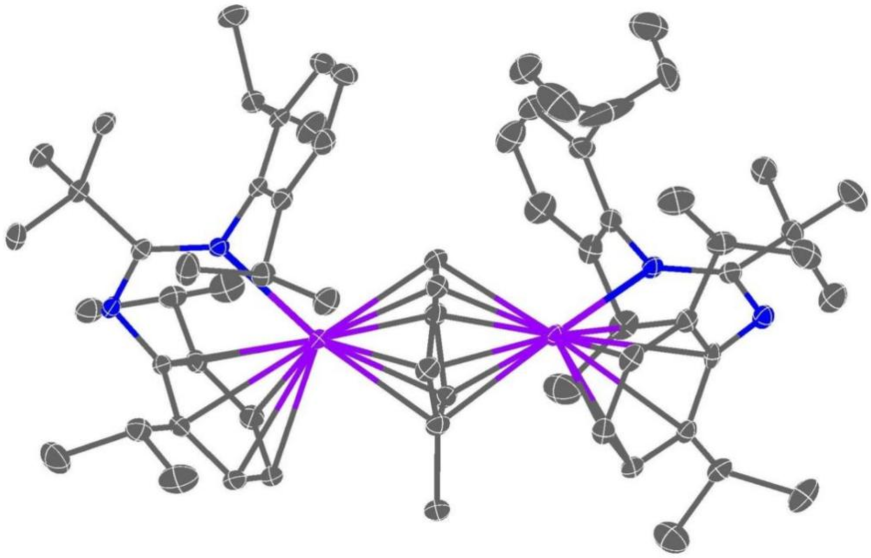

Figure S61 Molecular structure of **3-Tb** with selective atom labelling (C = gray, N = blue, Tb = violet). Thermal ellipsoids set at 50 % probability level and hydrogen atoms omitted for clarity.

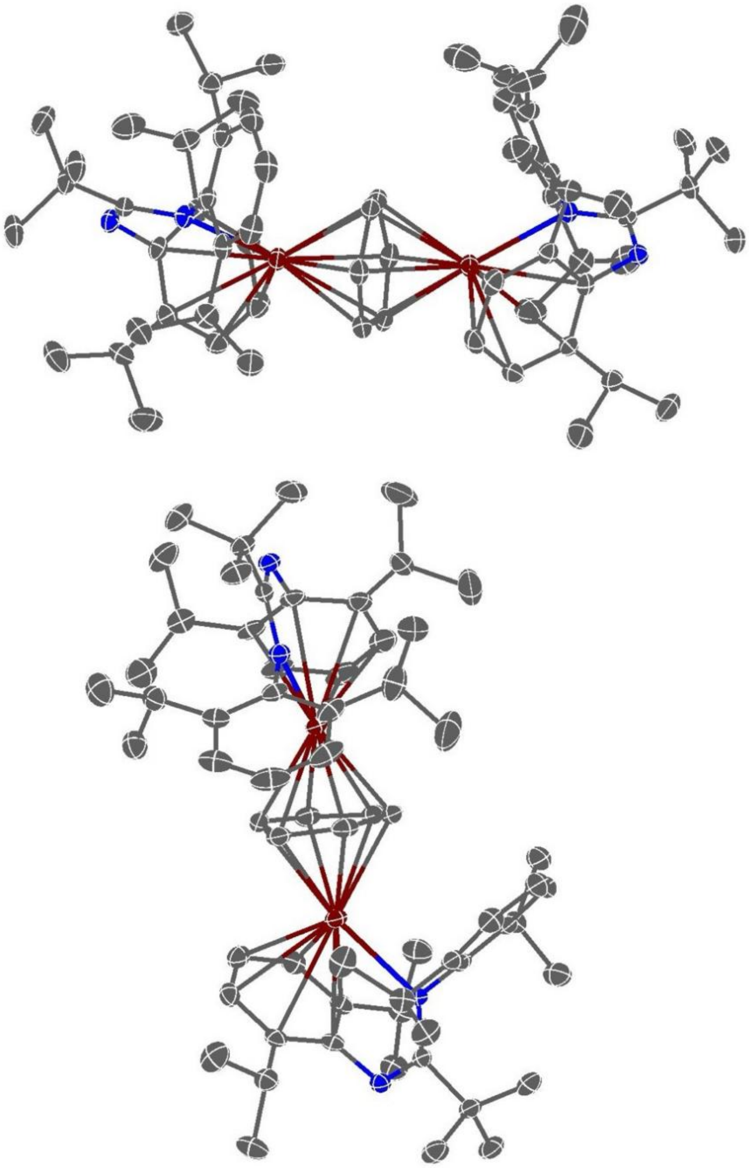

Figure S62 Molecular structure of **2-Gd** with selective atom labelling (C = gray, N = blue, Gd = wine). Thermal ellipsoids set at 50 % probability level and hydrogen atoms omitted for clarity.

5. Magnetism

5.1 Temperature-swept magnetic measurements

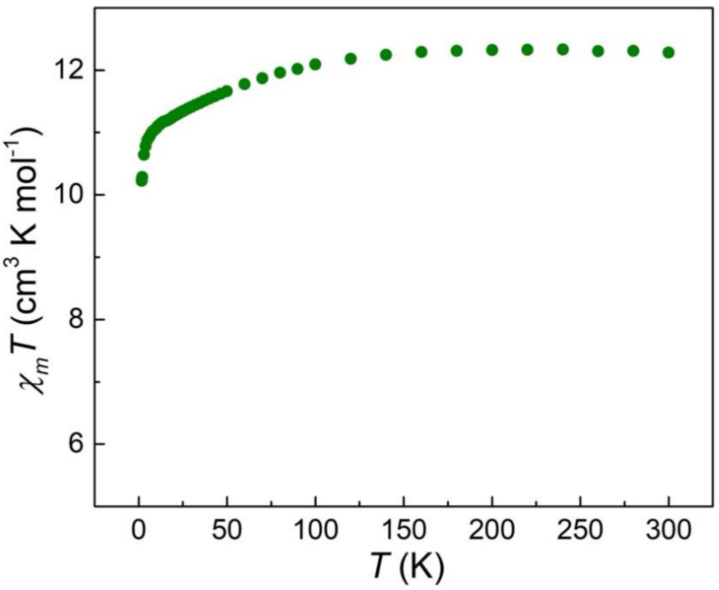

Figure S63 Temperature dependent susceptibility of $\chi_m T$ in an applied dc magnetic field of 1 kOe for **1-Tb-Cl**.

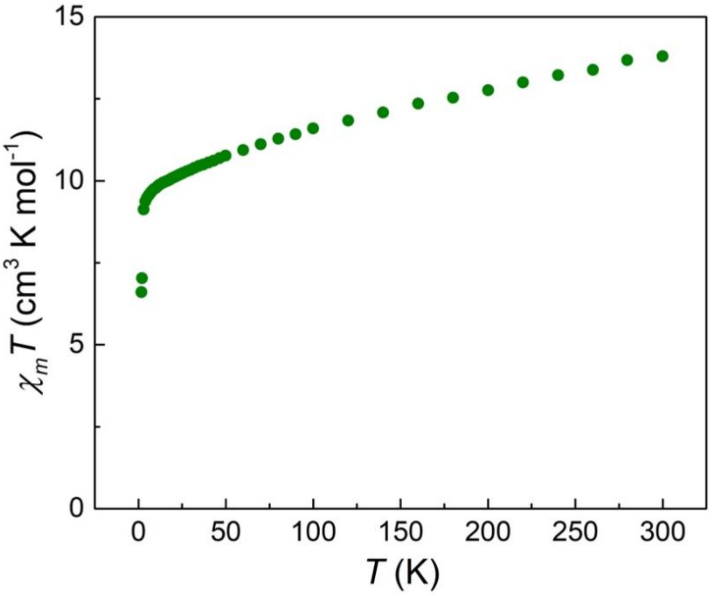

Figure S64 Temperature dependent susceptibility of $\chi_m T$ in an applied dc magnetic field of 1 kOe for **1-Dy-Cl**.

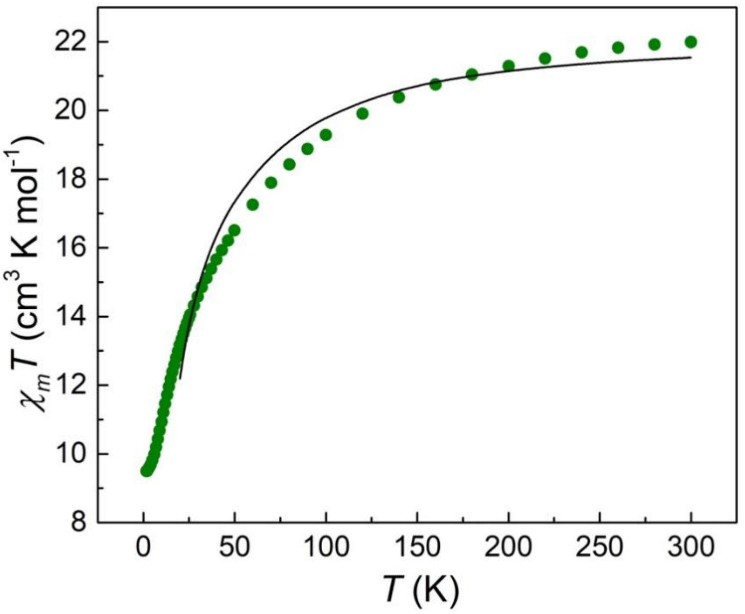

Figure S65 Temperature dependent susceptibility of $\chi_m T$ in an applied dc magnetic field of 1 kOe for **2-Tb**. The solid line represents the best fit through POLY_ANISO program.

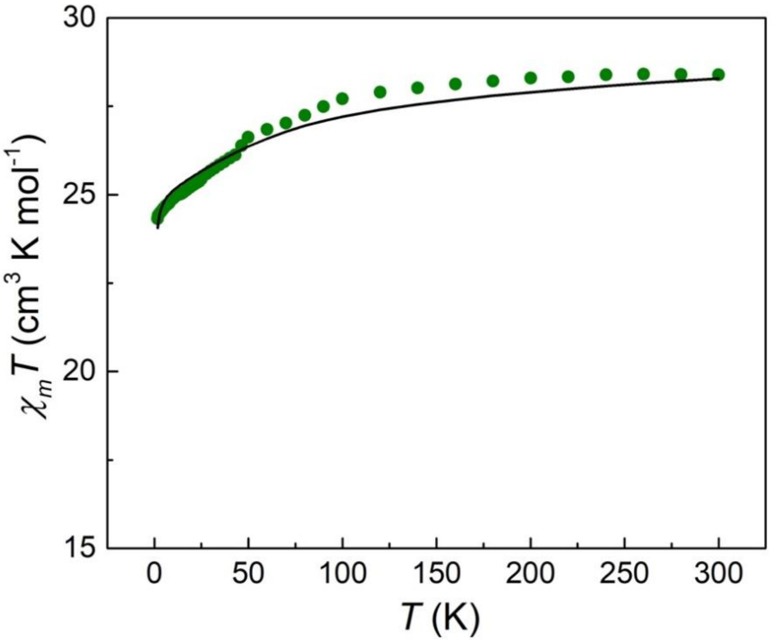

Figure S66 Temperature dependent susceptibility of $\chi_m T$ in an applied dc magnetic field of 1 kOe for **2-Dy**. The solid line represents the best fit through POLY_ANISO program.

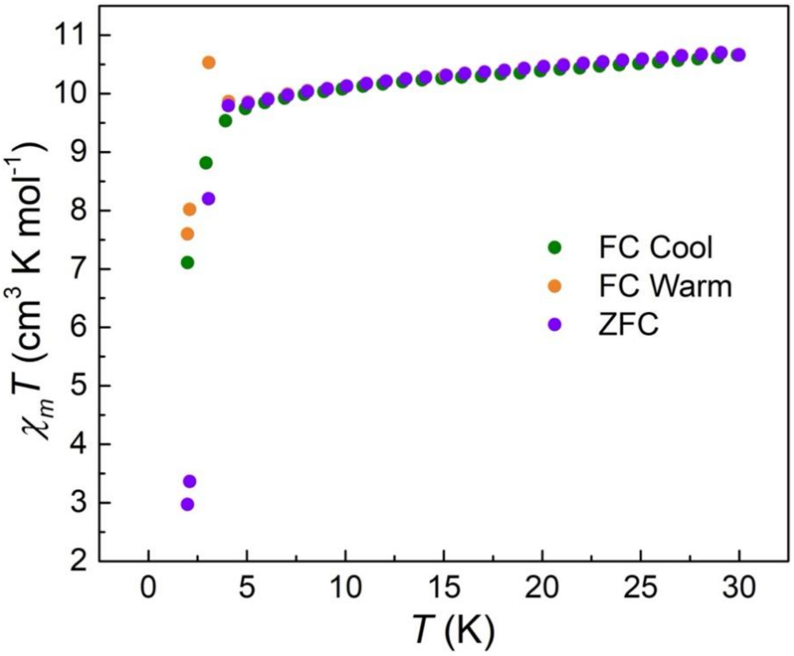

Figure S67 The χT (top) and χ (bottom) products as a function of temperature field-cooled (FC, green points) and zero-field-cooled (ZFC, blue points) variable-temperature magnetic susceptibility for **1-Dy-Cl** with 1 kOe dc field in warm mode from 2 to 30 K. The orange points represent FC variable-temperature magnetic susceptibility in cool mode from 30 to 2 K.

5.2 Ac magnetic measurements

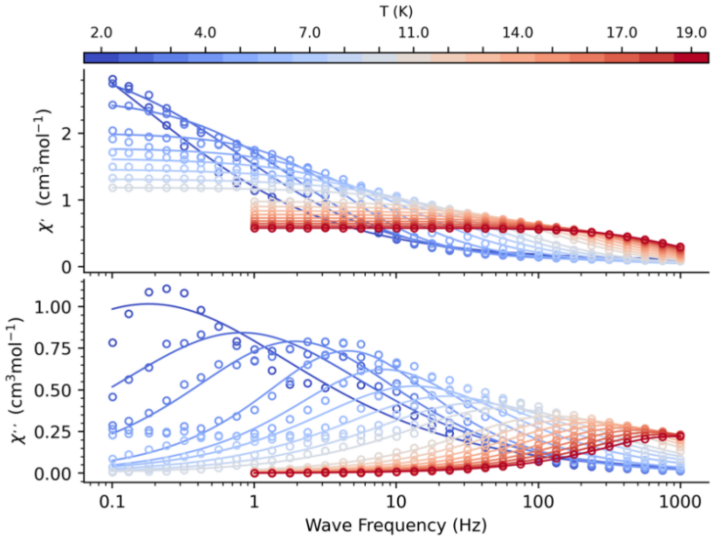

Figure S68 In-phase (χ′, top) and out-of-phase (χ″, bottom) components of the ac magnetic susceptibility for **1-Tb-Cl** under 2000 applied dc field at frequencies ranging from 0.1-1000 Hz and temperatures from 28-2 K. The colored lines are guides for the eye.

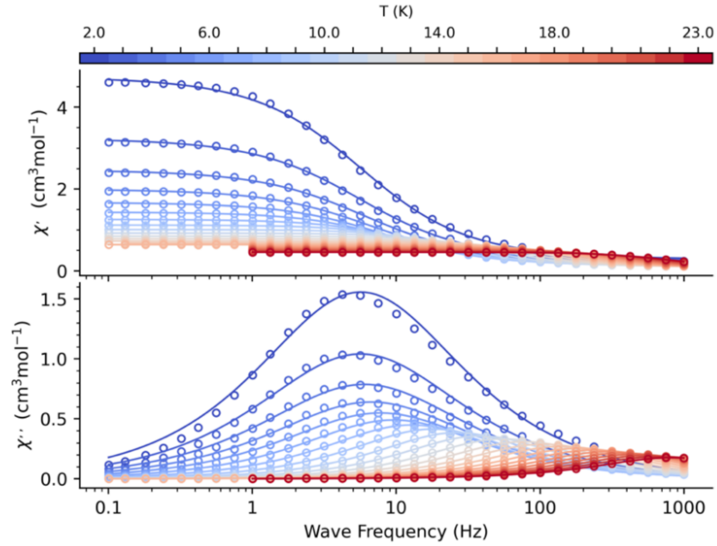

Figure S69 In-phase (χ′, top) and out-of-phase (χ″, bottom) components of the ac magnetic susceptibility for **1-Dy-Cl** under zero applied dc field at frequencies ranging from 0.1-1000 Hz and temperatures from 130-65 K. The colored lines are guides for the eye.

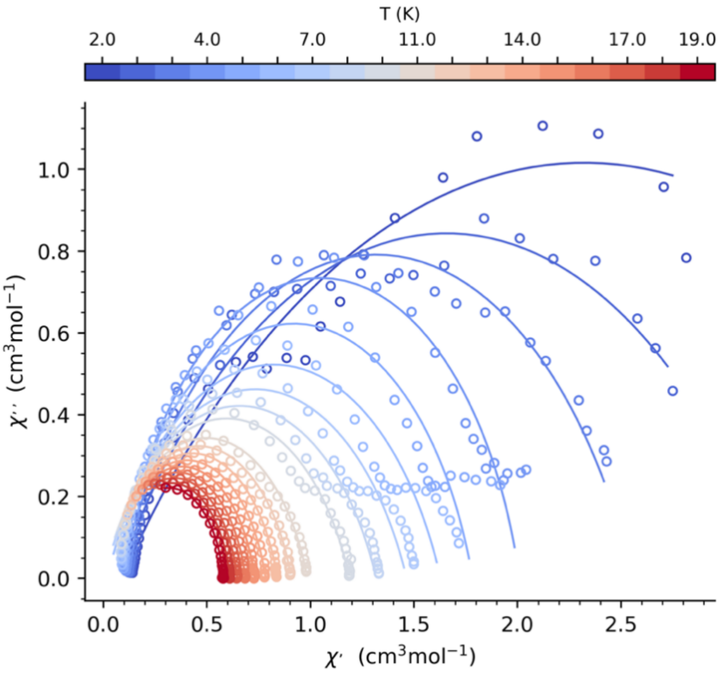

Figure S70 Cole-Cole plots for **1-Tb-Cl** from 42-26 K under 1500 applied dc field. The colored lines are fits to generalized Debye model.

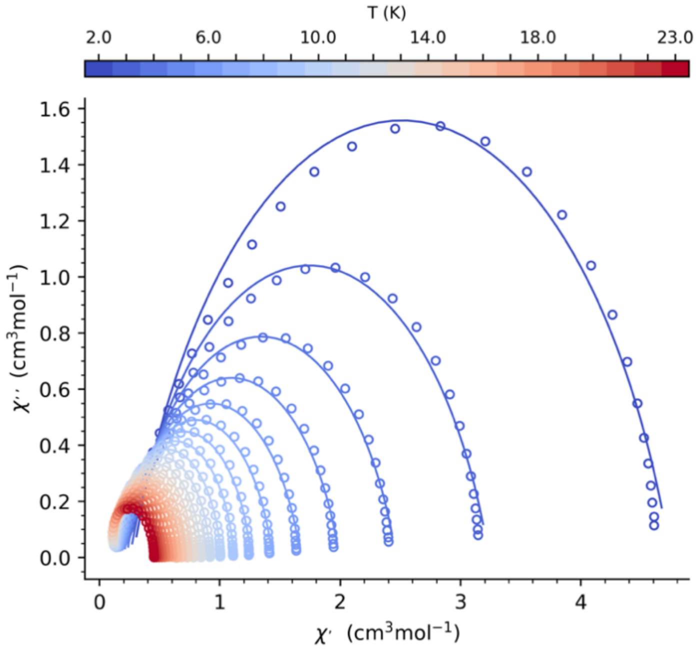

Figure S71 Cole-Cole plots for **1-Dy-Cl** from 64-10 K under zero Oe applied dc field. The colored lines are fits to generalized Debye model.

Table S4 Parameters used to fit ac magnetic relaxation data for **1-Tb-Cl** at 2000 Oe applied field and magnetic relaxation times extracted from these fits.

T	χ_S	χ_s^{err}	χ_T	χ_T^{err}	τ_{debye}	τ_{debye}^{err}	α	α^{err}
(K)	(cm^3 mol^{-1})		(cm^3 mol^{-1})			(s)		
2	0.08858	0.02348	4.55617	0.23313	0.88171	0.14452	0.45651	0.01969
2.5	0.06898	0.01537	3.24541	0.05387	0.19506	0.00966	0.37844	0.01241
3	0.07876	0.01131	2.55862	0.02296	0.0823	0.00221	0.27668	0.01019
4	0.08685	0.01456	2.0098	0.01941	0.03506	0.0011	0.1695	0.01507
5	0.05748	0.02305	1.78324	0.02494	0.01963	0.00102	0.2042	0.02354
6	0.01969	0.02479	1.62878	0.02196	0.01179	6.78484E-4	0.26654	0.02334
7	0.00372	0.02189	1.4649	0.01555	0.00701	3.58157E-4	0.28161	0.01995
8	2.1939E-8	0.0172	1.31792	0.00995	0.00435	1.75588E-4	0.27511	0.01569
9	0.00157	0.01337	1.18938	0.00638	0.00279	8.80962E-5	0.25888	0.01237
11	0.00941	0.00898	0.985	0.00412	0.00127	2.66372E-5	0.21945	0.00914
12	0.01277	0.00726	0.90755	0.00271	9.0873E-4	1.54781E-5	0.20319	0.00731
13	0.01803	0.00587	0.84	0.00179	6.6979E-4	9.36112E-6	0.18573	0.00586
14	0.02212	0.0057	0.78329	0.00146	5.0740E-4	7.03614E-6	0.17336	0.00559
15	0.0268	0.00439	0.73067	8.9411E-4	3.9177E-4	4.32507E-6	0.1595	0.00419
16	0.03418	0.00548	0.68578	9.1681E-4	3.1243E-4	4.45748E-6	0.14475	0.00513
17	0.03955	0.00535	0.64666	7.2843E-4	2.5314E-4	3.6737E-6	0.13458	0.00485
18	0.04716	0.00604	0.6111	6.7661E-4	2.1021E-4	3.59065E-6	0.12064	0.00536
19	0.05334	0.00646	0.57968	5.9037E-4	1.7650E-4	3.37189E-6	0.11013	0.00555

Table S5 Parameters used to fit ac magnetic relaxation data for **1-Dy-Cl** at zero applied field and magnetic relaxation times extracted from these fits.

T	χ_S	χ_S^{err}	χ_T	χ_T^{err}	τ_{debye}	τ_{debye}^{err}	α	α^{err}
(K)	(cm^3 mol^{-1})		(cm^3 mol^{-1})		(s)			
2	0.27799	0.01727	4.74477	0.02153	0.02811	4.62218E-4	0.22456	0.00719
3	0.25288	0.01102	3.2364	0.01375	0.02815	4.4242E-4	0.22443	0.00687
4	0.22347	0.00789	2.46454	0.0096	0.02648	3.90097E-4	0.22045	0.00649
5	0.19968	0.0065	1.99309	0.00757	0.02383	3.4976E-4	0.21063	0.00659
6	0.18185	0.00568	1.67096	0.00624	0.02053	3.01578E-4	0.19129	0.00683
7	0.16695	0.00485	1.43565	0.00492	0.0166	2.27839E-4	0.16259	0.00669
8	0.15244	0.00379	1.25717	0.00349	0.01246	1.40898E-4	0.13197	0.0058
9	0.13831	0.00272	1.11829	0.00223	0.0088	7.35967E-5	0.10582	0.00447
10	0.12542	0.00186	1.00757	0.00135	0.00603	3.49289E-5	0.08724	0.00319
11	0.11466	0.00124	0.91956	7.91028E-4	0.00416	1.61547E-5	0.07591	0.00218
12	0.10492	8.66883E-4	0.84482	4.85608E-4	0.00289	7.86497E-6	0.06896	0.00154
13	0.09744	5.39187E-4	0.78168	2.64103E-4	0.00206	3.47777E-6	0.0646	9.62458E-4
14	0.09104	4.51625E-4	0.72768	1.9262E-4	0.0015	2.11657E-6	0.06234	8.01658E-4
15	0.08513	5.91598E-4	0.68038	2.24181E-4	0.00112	2.05509E-6	0.06106	0.00104
16	0.08105	4.56865E-4	0.63915	1.45291E-4	8.54715E-4	1.21053E-6	0.06021	7.85192E-4
17	0.07687	3.99749E-4	0.60327	1.37394E-4	6.65136E-4	8.20565E-7	0.06099	6.91405E-4
18	0.07395	4.94375E-4	0.57121	1.43011E-4	5.27053E-4	8.12787E-7	0.06118	8.26086E-4
19	0.0717	5.73557E-4	0.54275	1.38787E-4	4.24088E-4	7.73354E-7	0.06116	9.20872E-4
20	0.07014	0.00101	0.51663	2.01845E-4	3.4476E-4	1.13444E-6	0.06051	0.00155
21	0.06713	8.26405E-4	0.49333	1.34791E-4	2.81413E-4	7.86725E-7	0.0623	0.00119
22	0.06568	0.00116	0.47198	1.52428E-4	2.31986E-4	9.46628E-7	0.06288	0.00157
23	0.06353	0.00151	0.4527	1.57399E-4	1.91081E-4	1.06297E-6	0.06431	0.0019

5.3 Magnetic relaxation profiles

The representative time and width of the relaxation time distributions are defined using the expectation value ($\langle \ln\tau \rangle$) and variance ($\sigma^2_{\ln\tau}$) of the distribution of logarithmic relaxation times.[1,2] These values are calculated for relaxation time distributions characterized by different empirical formulae using equations given by Zorn.[3]

For a generalized Debye model:

$$\boldsymbol{\langle \ln\tau \rangle = \ln \tau_{\text{debye}}}$$

$$\boldsymbol{\sigma^2_{\ln\tau} = \frac{\pi^2}{3}\left(\frac{1}{(1-\alpha)^2} - 1\right)}$$

Equation S2

The representative (central) value of τ is given by $e^{\langle \ln\tau \rangle}$ and the one estimated standard deviation (ESD) upper and lower values are given by:[1,2]

$$\tau_{\pm} = e^{\langle \ln\tau \rangle \pm \sqrt{\sigma^2_{\ln\tau}}}$$

Equation S4

Values of $e^{\langle \ln\tau \rangle}$, $\tau_{\pm}$, $\sigma^2_{\ln\tau}$and the uncertainty $e^{\langle \ln\tau \rangle\,\mathrm{err}}$ are compared across temperatures and measurement methods in Table S5 and Table S6, respectively. We use the one ESD values represent the distributions in relaxation times and are distinct from the uncertainty in the central relaxation time.

Table S6 Relaxation times and distributions for **1-Tb-Cl** at 2000 Oe applied field.

T (K)	τ_- (s)	$e^{\langle \ln\tau \rangle}$ (s)	τ_+ (s)	$e^{\langle \ln\tau \rangle \text{ err}}$ (s)	$\sigma^2_{\ln\tau}$
**Ac Measurements**					
2	5.3618E-02	0.88171	14.49908	0.14452	7.83987
2.5	1.9856E-02	0.19506	1.91624	0.00966	5.22038
3	1.4581E-02	0.0823	0.46453	0.00221	2.99517
4	1.0393E-02	0.03506	0.11827	0.0011	1.47842
5	4.9410E-03	0.01963	0.07799	0.00102	1.90302
6	2.1972E-03	0.01179	0.06327	0.00067848	2.82268
7	1.2115E-03	0.00701	0.04056	0.00035816	3.08168
8	7.7677E-04	0.00435	0.02436	0.00017559	2.96798
9	5.3998E-04	0.00279	0.01442	8.8096E-05	2.69704
11	2.9736E-04	0.00127	0.00542	2.6637E-05	2.10779
12	2.2983E-04	0.00090873	0.00359	1.5478E-05	1.88987
13	1.8393E-04	0.00066979	0.00244	9.3611E-06	1.67026
14	0.00015	0.0005074	0.00174	7.0361E-06	1.52303
15	0.00012	0.00039177	0.00126	4.3251E-06	1.36571
16	0.00010	0.00031244	0.00094	4.4575E-06	1.20663
17	8.86201E-05	0.00025314	0.0007231	3.6737E-06	1.10164417
18	7.87632E-05	0.00021021	0.000561	3.5907E-06	0.96362029
19	6.96781E-05	0.0001765	0.00044707	3.3719E-06	0.86381846

Table S7 Relaxation times and distributions for **1-Dy-Cl** at zero applied field.

T (K)	$\boldsymbol{\tau_{-}}$ (s)	$\boldsymbol{e^{\langle \ln\tau \rangle}}$ (s)	$\boldsymbol{\tau_{+}}$ (s)	$\boldsymbol{e^{\langle \ln\tau \rangle \mathrm{err}}}$ (s)	$\boldsymbol{\sigma^2_{\ln\tau}}$
**Ac Measurements**					
2	6.4234E-03	0.02811	0.12302	4.62218E-4	2.17911
3	6.4365E-03	0.02815	0.12311	4.4242E-4	2.17728
4	6.1707E-03	0.02648	0.11363	3.90097E-4	2.12163
5	5.8183E-03	0.02383	0.09760	3.4976E-4	1.98791
6	5.4921E-03	0.02053	0.07674	3.01578E-4	1.73866
7	5.0842E-03	1.66E-02	0.05420	2.27839E-4	1.40011
8	4.4174E-03	1.25E-02	0.03515	1.40898E-4	1.07530
9	3.5504E-03	8.80E-03	0.02181	7.35967E-5	0.82391
10	2.6789E-03	6.03E-03	0.01357	3.49289E-5	0.65827
11	1.9655E-03	4.16E-03	0.00880	1.61547E-5	0.56213
12	1.4201E-03	2.89E-03	0.00588	7.86497E-6	0.50488
13	1.0381E-03	2.06E-03	0.00409	3.47777E-6	0.46962
14	0.00077	1.50E-03	0.00294	2.11657E-6	0.45154
15	0.00058	1.12E-03	0.00218	2.05509E-6	0.44135
16	0.00044	8.55E-04	0.00165	1.21053E-6	0.43461
17	0.00034243	6.65E-04	0.00129196	8.20565E-7	0.44079462
18	0.000271033	5.27E-04	0.00102491	8.12787E-7	0.44230346
19	0.00021811	4.24E-04	0.00082459	7.73354E-7	0.44214459
20	0.000178003	3.45E-04	0.00066774	1.13444E-6	0.4369869
21	0.000143753	2.81E-04	0.0005509	7.86725E-7	0.45121632
22	0.000118098	2.32E-04	0.0004557	9.46628E-7	0.45584447
23	9.64582E-05	1.91E-04	0.00037853	1.06297E-6	0.46729204

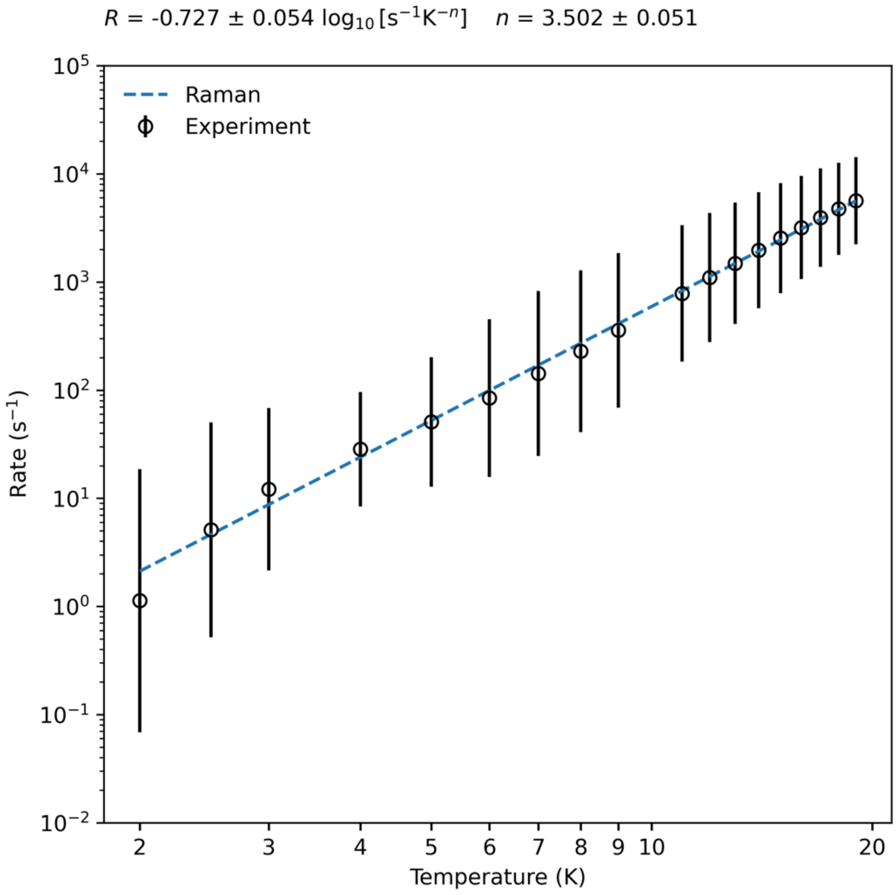

Figure S72 Plot of natural log of the inverse relaxation time *vs*. temperature for crystalline **1-Tb-Cl** under 1500 Oe dc magnetic field. The dashed blue line shows $\tau^{-1} = CT^n$, where $C = 10^{-0.727 \pm 0.054}$ (1.87×10^{-1}) s^{-1} K^{-n} and $n = 3.502 \pm 0.051$.

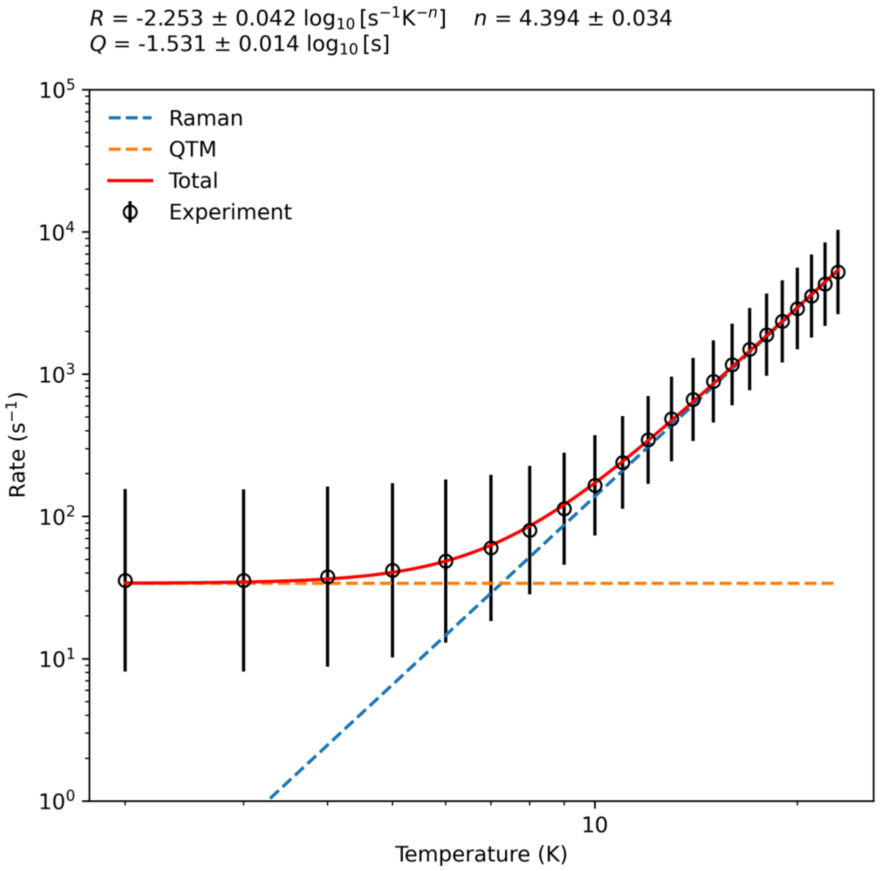

Figure S73 Plot of natural log of the inverse relaxation time *vs*. temperature for crystalline **1-Dy-Cl** under zero Oe dc magnetic field. The dashed blue line shows $\tau^{-1} = CT^n$, where *C* = $10^{-2.253 \pm 0.042}$ (5.58×10^{-3}) s^{-1} K^{-n} and *n* = 4.394 ± 0.034. The dashed orange line shows $\tau^{-1} = \tau^{-1}{}_{QTM}$, where τ_{QTM} = $10^{-1.531 \pm 0.014}$ (2.94×10^{-2}) s. Red solid line shows their sum with $\tau^{-1} = CT^n + \tau^{-1}{}_{QTM}$.

5.4 Field-swept magnetic measurements

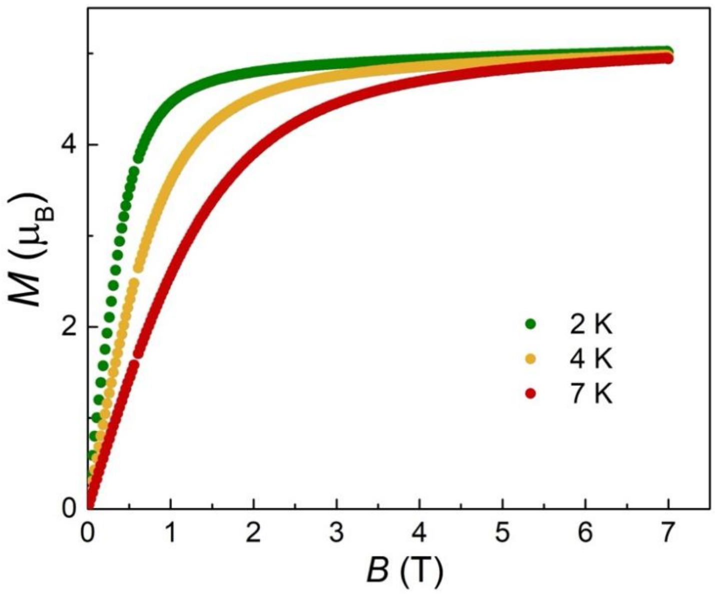

Figure S74 Field dependent magnetization at 2.0 K up to 7 Tesla for **1-Tb-Cl**.

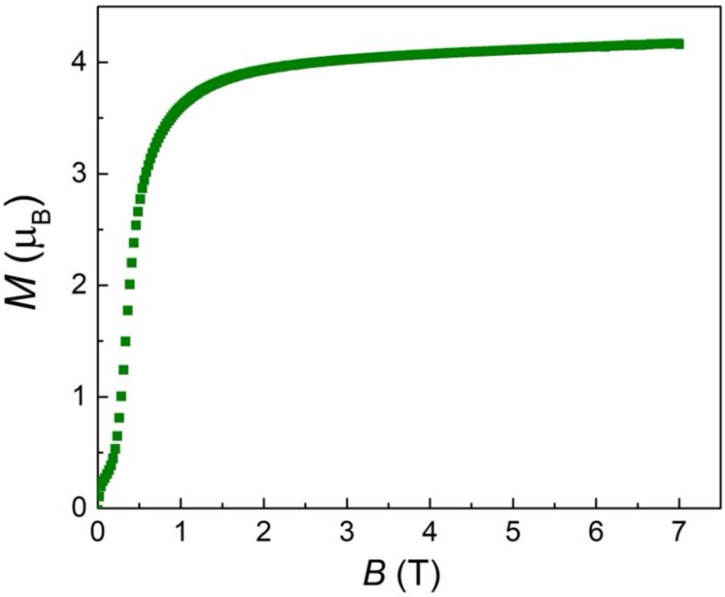

Figure S75 Field dependent magnetization at 2.0 K up to 7 Tesla for **1-Dy-Cl**.

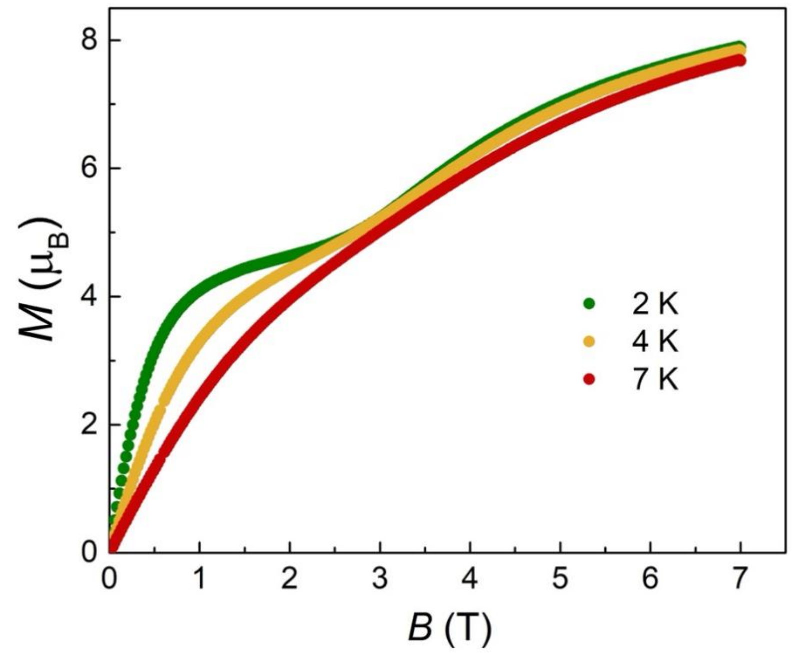

Figure S76 Field dependent magnetization at 2.0 K up to 7 Tesla for **2-Tb**.

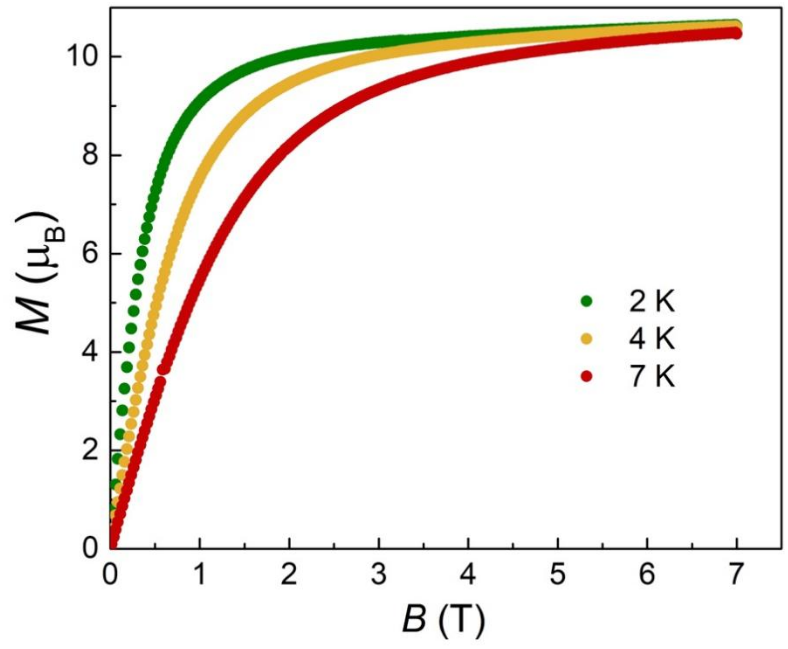

Figure S77 Field dependent magnetization at 2.0 K up to 7 Tesla for **2-Dy.**

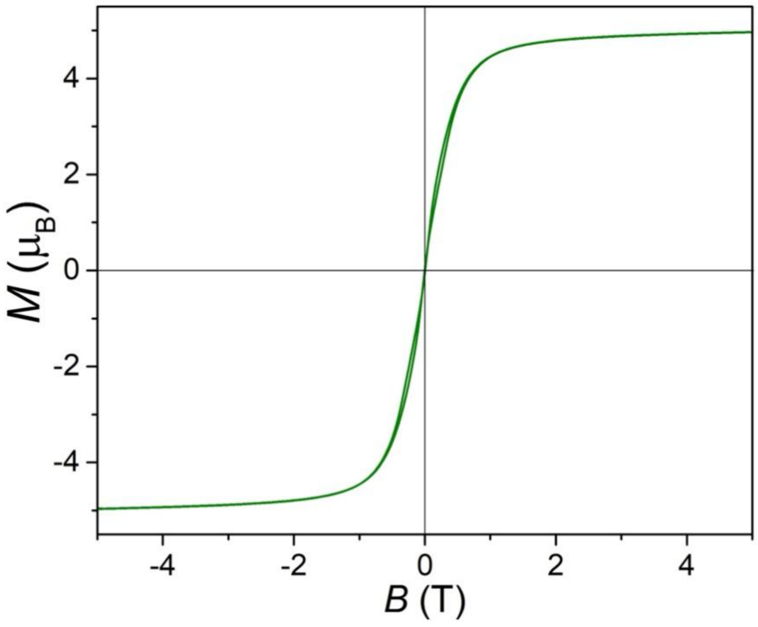

Figure S78 Magnetic hysteresis loop measurements for **1-Tb-Cl** at an average sweep rate of 22 Oe/s at 2 K.

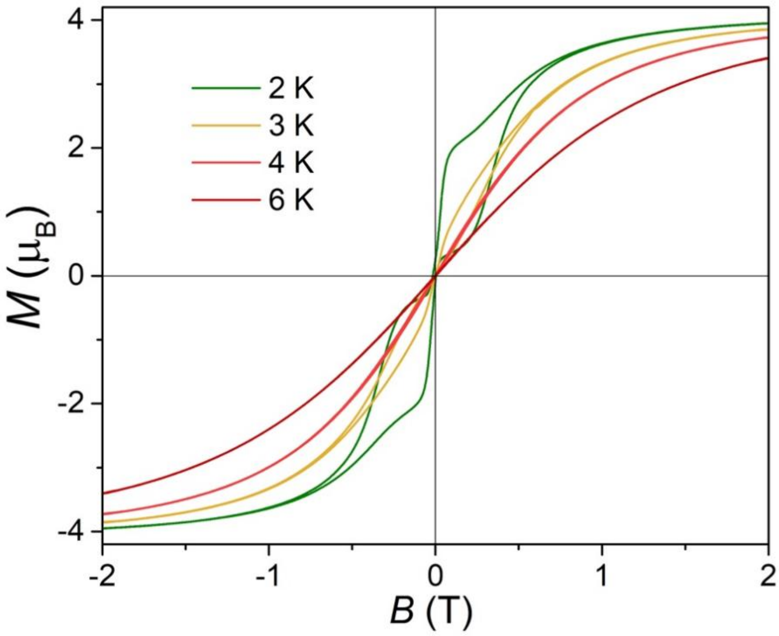

Figure S79 Magnetic hysteresis loop measurements for **1-Dy-Cl** at an average sweep rate of 22 Oe/s in a temperature range of 2 K to 10 K.

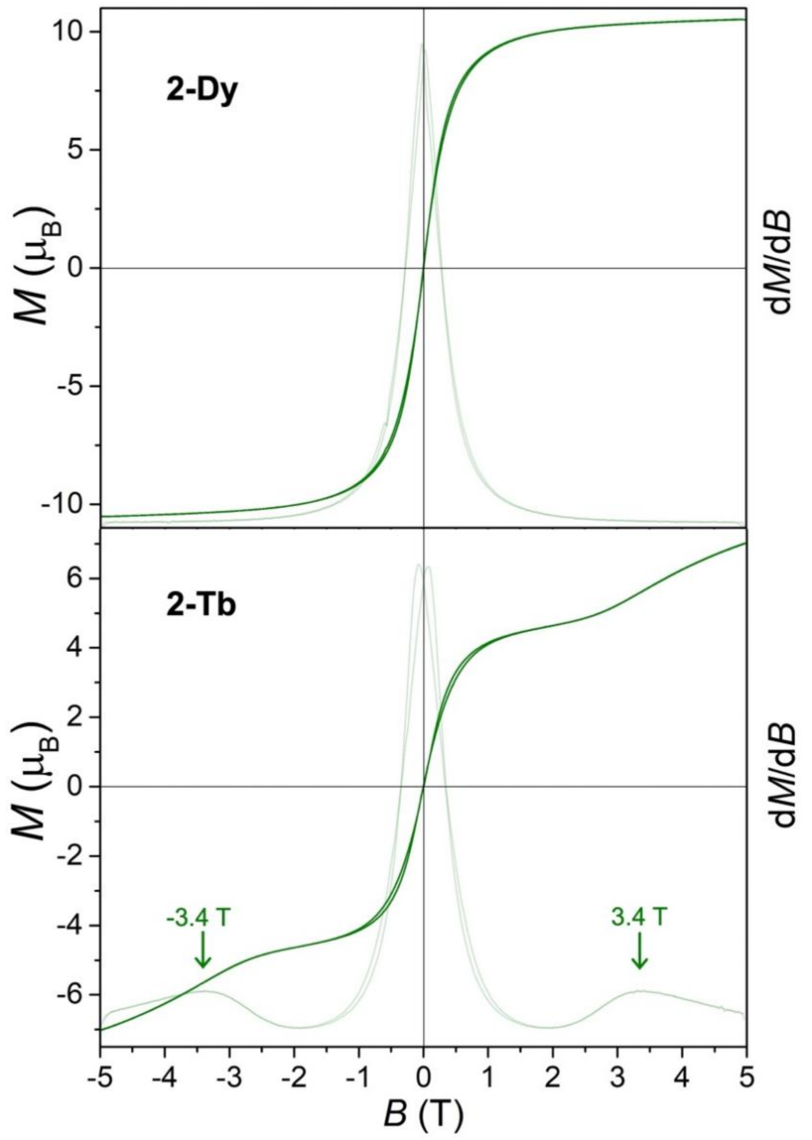

Figure S80 Magnetization M (μ_B) *vs*. applied dc field B (T) plots and their first derivatives for **2-Dy** (above) and **2-Tb** (bottom) at 2 K under an average sweep rate of 22 Oe/s.

6. EPR spectroscopy

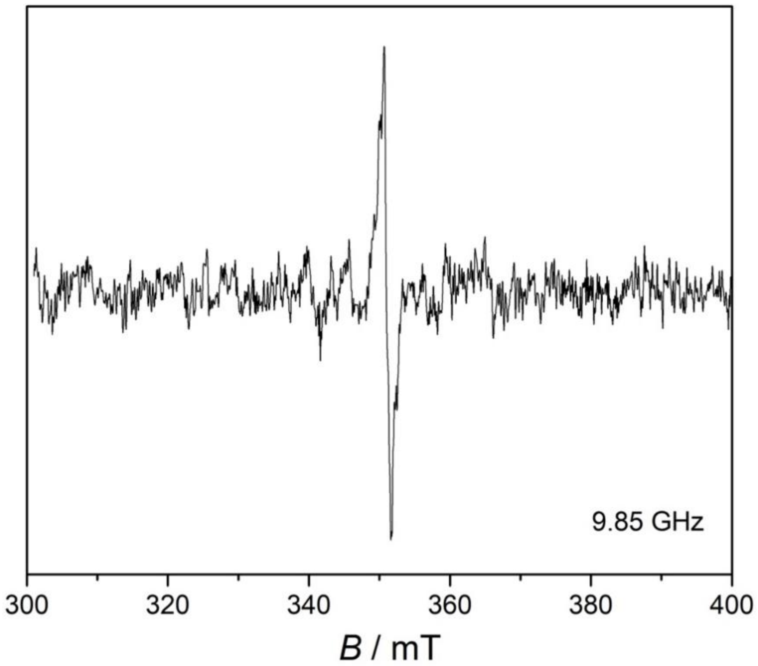

Figure S81 X–band (9.85 GHz) cw EPR spectrum of solution sample [Y(Piso)$_2$] intermediate at 300 K.

7. Calculations

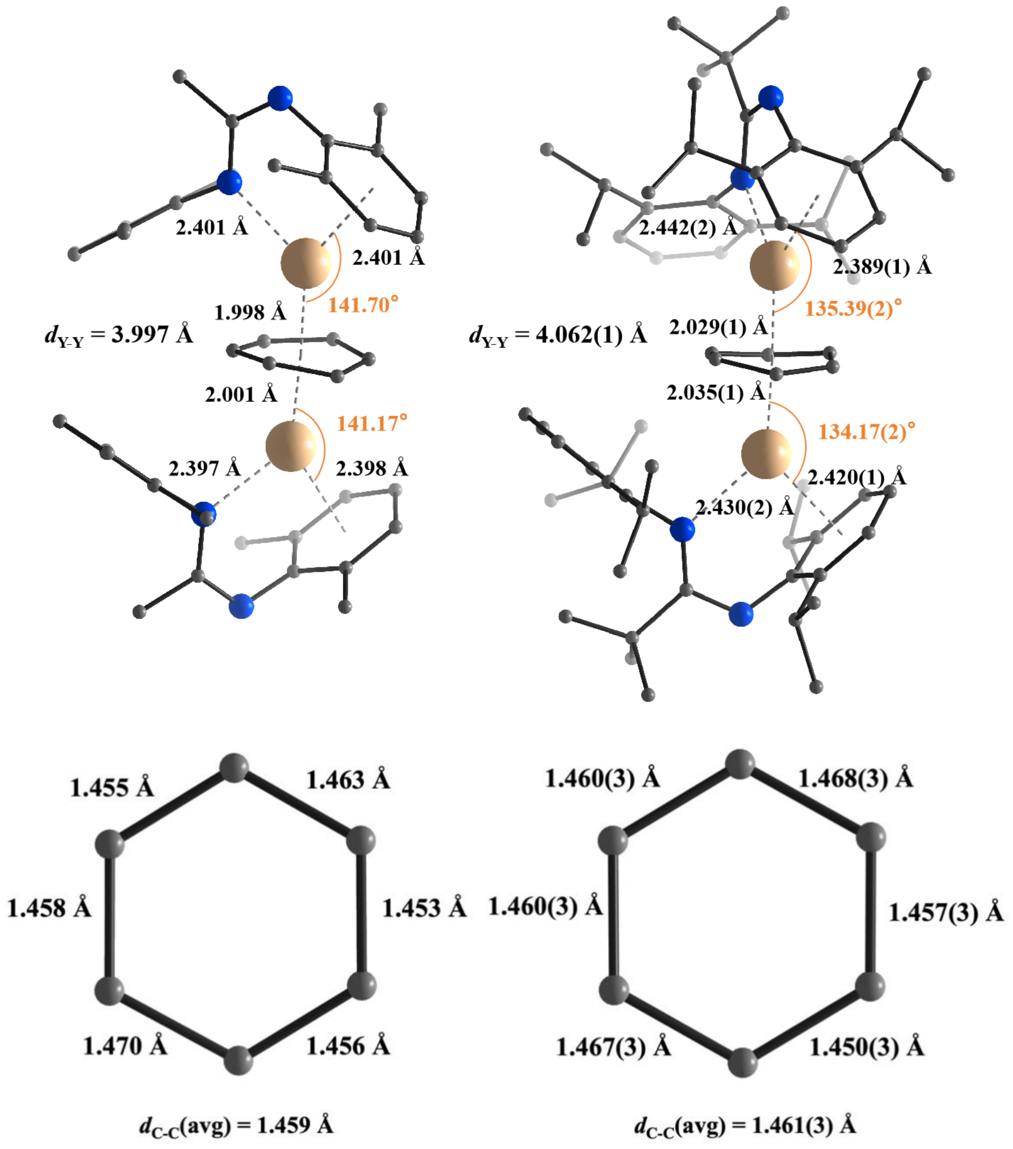

Figure S82 The comparison of key structural parameters between the single-crystal structure of **2-Y** (right) and its optimized model complex **2-Y^{opt}** (left). Color codes: Y, tan; N, blue; C, gray. For clarity, all hydrogen atoms and surrounding solvent molecules are omitted.

Table S8 Optimized geometric coordinates for the model complex **2-Y^{opt}** by DFT calculations.

Y	4.12560669796898	12.48335813703601	6.37021192157980
Y	4.08250805886668	14.05392611067283	2.69491853632911
N	4.22621375305471	13.70333926602815	8.43532026596866
C	2.67632652064667	13.28192365111423	4.64633681551100
C	4.96053960691836	14.36912354829744	4.91858398454392
C	5.21637157385810	9.97905774399392	6.99551971994115
C	3.31453454593670	12.15166560750040	3.95704294382530
C	3.51023690439250	14.41631487561713	5.02647444497982
C	5.59411668122828	13.10730479858010	4.53483823565939
C	4.00984931958249	9.74329738508227	6.33174990294896
C	5.24659901880872	10.65511794504817	8.21139571202458
C	4.76430857366861	12.03530145598985	4.01156084849837
C	4.03022878809763	11.11843999265151	8.77502029841779
C	2.80766915443292	10.12455691938527	6.93129481965945
C	2.80180190834410	10.80190354473499	8.15237518963611
N	2.57738296691179	15.74181223070776	1.90076625939387
C	4.29912835792003	14.94245741289287	0.09422675635433
C	5.54241819010070	15.28419113943253	0.67668922859341
C	6.37109444490490	14.27190317618071	1.16188234553984
C	3.95463451412784	13.57899929908171	-0.08335969252801
C	4.80296612768661	12.59421924751836	0.41518111057457
C	5.99069466894249	12.93137039861137	1.06979285450256
C	4.15359692156980	13.18509318885365	9.67795014298872
C	4.33430447348512	15.10185522183103	8.28026517508490
H	1.60779005142708	13.42736524963983	4.56171426670149
H	5.54664993275218	15.14106668649757	5.39881673221568
H	6.14780011407071	9.65088020846603	6.54680494760304
H	2.72386941709854	11.27556065018586	3.72355309851817
H	3.03877324726119	15.31187800774065	5.40079297666736
H	6.66572507441238	13.07117122016510	4.39066697480315
H	4.00546639349362	9.23760835635582	5.37502753039968
C	6.53171676056664	10.93565760057262	8.91615150296413
H	5.23687702078516	11.14354890484484	3.62478213227170
N	4.04981163848688	11.90364441619147	9.90348967278515
H	1.86664339283832	9.90695967255018	6.43859798550574
C	1.52625573177686	11.25060401514189	8.78450704964546
C	2.58081434576479	16.29436812718717	0.67000880507596
C	1.63890856424970	16.20254581066329	2.84911684505105
N	3.41402973615899	15.93070696875309	-0.26618336269480
C	5.91796985518449	16.72331323359009	0.80482696810275
H	7.31294529332128	14.53429230373107	1.63094859162181
C	2.66996829240360	13.23433271238128	-0.76036642127564
H	4.52606382202018	11.55097234085850	0.30961133507188
H	6.62932411510416	12.15614240525654	1.47210168869950
C	4.18467358934441	14.08459755960448	10.87597917880644
C	3.17924581726627	15.88073891955658	8.11336452810501
C	5.60044326942538	15.69789101070098	8.18412008111241
H	6.71318392735919	12.01017613494763	8.99148052089025
H	1.47906550219376	12.34102372797893	8.84168866617983
C	1.60991839442249	17.38293183076560	0.32589578694846

C	0.39281109355762	15.56945080296406	2.96088220963910
C	1.98706948653024	17.22819228277517	3.74033115948946
H	5.22628434209348	17.25246983481716	1.46490337372100
H	1.81567838506135	13.61427439461666	-0.19507317044686
C	3.30992503164205	17.24137028827553	7.86370123754936
C	1.82286946946831	15.25375562961468	8.17535236374350
C	5.69365233558177	17.06084920365919	7.93005472272052
C	6.83773651347078	14.86887440398645	8.32634293941483
C	0.02888905597089	14.45345638836511	2.03338135344411
C	-0.49222185817083	15.98045713149598	3.95039153091294
C	3.33234205640734	17.87700885047725	3.66490341386977
C	1.07555145532284	17.61659195238735	4.71445753558243
H	2.41375071513018	17.83923978680239	7.73372608482785
C	4.55700526886713	17.83453516772439	7.76945088167536
H	1.62513538768556	14.80540802241345	9.15373818630161
H	6.67555369339909	17.51769583118072	7.85358706436132
H	6.86248566580126	14.06942052707424	7.57719194057223
H	0.76733911355409	13.64580654013071	2.08338738738096
C	-0.15832346659092	16.99932945030345	4.82583974547551
H	-1.45628307650646	15.48823591925728	4.03385184584987
H	3.51562290420174	18.31915856970414	2.68160668241654
H	1.34743703816484	18.41292422907534	5.39974392851164
H	4.64332222294633	18.89725442210932	7.57152225894263
H	-0.85885130752618	17.31187717065397	5.59241204265041
H	-0.94941757318743	14.04288478847984	2.28882397539572
H	-0.00363158421989	14.78565402401493	0.99123166741864
H	3.42402784010907	18.66158436698803	4.41766582373945
H	4.13058409719548	17.14740342289738	3.84432263026424
H	2.62629888383229	13.70705781874206	-1.74487019236117
H	2.56545348122303	12.15414955368278	-0.87158982225656
H	6.92812474268826	16.82804886308845	1.20324371784658
H	5.85655906738573	17.21477629900600	-0.16927221575946
H	1.75633231306734	17.68009056579013	-0.71054856013834
H	0.57929192068705	17.05082109749859	0.47400056135836
H	1.75574860095756	18.24746856380044	0.97921619884169
H	6.48808984219323	10.55495452841591	9.93984621380626
H	7.37062569143220	10.47426452999671	8.39299012461244
H	0.66479617521286	10.89241179705892	8.21912954389846
H	1.46684334444447	10.88262126885194	9.81185006414549
H	5.11752417861055	14.65386626328913	10.90895139731950
H	3.37391461714446	14.81635594929459	10.83174400677909
H	4.09095502273307	13.48423069316182	11.77863159741349
H	1.04753663032650	15.99412838800477	7.97319348281373
H	1.72597820610560	14.45482312568438	7.43224274599258
H	7.73089281129738	15.48354675575701	8.20211846562007
H	6.89103035405489	14.38394054546007	9.30545781283925

Table S9 Calculated Mayer bond orders (MBOs) of the coordinated bonds and C-C bonds on the bound arene in **2-Y^{opt}**.

Y1-N3	Y2-N16	Y1-C4	Y1-C5	Y1-C7	Y1-C8	Y1-C9	Y1-C12
0.1450	0.1471	0.1890	0.1447	0.1335	0.1093	0.2209	0.1578
Y2-C4	Y2-C5	Y2-C7	Y2-C8	Y2-C9	Y2-C12	Y1-C6	Y1-C10
0.1176	0.1656	0.2263	0.1414	0.1436	0.1632	0.0791	0.1631
Y1-C11	Y1-C13	Y1-C14	Y1-C15	Y2-C17	Y2-C18	Y2-C19	Y2-C20
0.0746	0.1994	0.1000	0.0450	0.1988	0.0538	0.0915	0.0686
Y2-C21	Y2-C22	C4-C7	C4-C8	C5-C8	C5-C9	C9-C12	C7-C12
0.0866	0.1669	1.1211	1.0570	1.1027	1.0992	1.0419	1.0516
**Avg. Y-N**	**Avg. Y-C**	**Avg. C-C**					
**0.1461**	**0.1350**	**1.0789**					

Table S10 TDDFT calculated electronic excitation in **2-Y^{opt}**. Only transitions with oscillator strengths (*f*) larger than 0.035 are summarized.

Wavelength (nm)	Oscillator strength *f*	Dominant Contributions (>20%)
583.06	0.42125	HOMO-1→LUMO (34.3%); HOMO→LUMO+3 (31.9%); HOMO→LUMO+1 (21.9%)
555.90	0.15928	HOMO→LUMO+3 (53.4%)
426.98	0.07862	HOMO→LUMO+4 (78.8%)
341.17	0.03925	HOMO→LUMO+12 (59.7%)
303.03	0.04805	HOMO-1→LUMO+13 (51.6%)
285.88	0.06052	HOMO-1→LUMO+15 (64.7%)
281.60	0.05284	HOMO-5→LUMO (69.2%)
280.49	0.09942	HOMO-5→LUMO+1 (47.6%); HOMO→LUMO+17 (24.1%)
279.95	0.06339	HOMO→LUMO+17 (41.6%); HOMO-5→LUMO+1 (21.2%)
271.78	0.07514	HOMO-1→LUMO+17 (74.0%)
264.59	0.08912	HOMO-3→LUMO+2 (28.8%); HOMO→LUMO+19 (23.4%)
261.05	0.07993	HOMO→LUMO+20 (53.1%)
249.10	0.08984	HOMO-5→LUMO+3 (33.0%); HOMO-8→LUMO (23.0%)
248.86	0.05816	HOMO-5→LUMO+2 (38.6%); HOMO-8→LUMO+1 (20.6%)
241.71	0.08605	HOMO-1→LUMO+21 (23.2%)

Table S11 CASSCF calculated electronic states for **1-Dy-Cl**.

Ab initio Energy (cm $^{-1}$)	*Ab initio* Energy (K)	g_x	g_y	g_z	g_z Angle (°)	Crystal field Wavefunction
0	0	0.00	0.00	19.92	-	98.9%|±15/2>
204	293	0.02	0.02	17.33	12.36	88.9%|±13/2>+10.0%|±11/2>
407	585	0.71	1.17	13.54	0.77	70.5%|±11/2>+12.4%|±9/2>
507	729	2.70	6.16	11.77	89.76	26.3%|±9/2>+23.0%|±5/2>
570	819	1.40	3.40	11.43	70.50	31.9%|±9/2>+14.6%|±7/2>+15.1%|∓7/2>
665	956	0.58	0.81	13.13	89.56	28.7%|±7/2>+25.0%|∓3/2>+14.2%|∓5/2>+19.5%|∓9/2>
851	1223	0.30	0.48	15.79	80.09	14.0%|±5/2>+11.0%|±1/2>+14.0%|∓1/2>+17.0%|∓5/2>+25.8%|∓7/2>
1188	1708	0.02	0.03	19.21	81.88	21.2%|±1/2>+18.6%|∓1/2>+35.4%|∓3/2>+15.7%|∓5/2>

Only components with > 10% contribution are given, rounded to the nearest percent.

Table S12 CASSCF calculated electronic states for fragment 1 of **2-Dy**.

Ab initio Energy (cm $^{-1}$)	*Ab initio* Energy (K)	g_x	g_y	g_z	g_z Angle (°)	Crystal field Wavefunction
0	0	0.00	0.00	19.91	-	99.1%|±15/2>
177	254	0.04	0.05	17.25	13.10	89.7%|±13/2>
342	492	0.89	3.17	12.29	26.32	48.5%|±11/2>+16.7%|±9/2>
358	515	0.91	2.40	15.20	86.02	10.8%|±11/2>+37.3%|±1/2>+13.4%|∓3/2>
479	689	2.27	3.88	8.23	38.65	25.2%|±9/2>+16.2%|±7/2>+13.3%|∓7/2>
545	783	0.76	1.39	17.54	69.84	20.8%|±7/2>+35.2%|±5/2>+21.4%|±3/2>
588	845	2.38	3.87	12.95	62.04	11.7%|±5/2>+15.5%|∓7/2>+35.6%|∓9/2>
697	1002	0.52	1.77	18.49	84.81	16.3%|±5/2>+23.5%|±1/2>+25.2%|∓3/2>+16.6%|∓7/2>

Only components with > 10% contribution are given, rounded to the nearest percent.

Table S13 CASSCF calculated electronic states for fragment 2 of **2-Dy**.

Ab initio Energy (cm^{-1})	*Ab initio* Energy (K)	g_x	g_y	g_z	g_z Angle (°)	Crystal field Wavefunction
0	0	0.00	0.00	19.89	-	98.8%|±15/2>
163	234	0.02	0.03	17.28	11.83	92.1%|±13/2>
344	495	1.09	2.75	12.81	28.60	53.8%|±11/2>+12.9%|±9/2>+12.2%|±1/2>
368	529	1.05	2.52	14.67	89.31	14.5%|±11/2>+36.1%|±1/2>+23.6%|∓3/2>
488	702	3.74	5.47	8.13	58.62	21.1%|±9/2>+16.6%|±3/2>+13.8%|∓1/2>+13.3%|∓7/2>
542	779	0.01	0.77	17.01	67.96	19.8%|±7/2>+16.3%|±5/2>+22.1%|±3/2>
616	886	0.95	3.10	10.53	88.24	31.0%|±5/2>+16.3%|∓7/2>+13.7%|∓9/2>
683	982	1.08	5.68	15.18	85.90	13.1%|±5/2>+11.0%|±1/2>+17.7%|∓3/2>+26.7%|∓7/2>+13.0%|∓9/2>

Only components with > 10% contribution are given, rounded to the nearest percent.

Table S14 CASSCF calculated electronic states for **1-Tb-Cl**.

Levels	*Ab initio* Energy (cm^{-1})	*Ab initio* Energy (K)	g_z	g_z Angle (°)	Δ_{tun} (cm^{-1})	Crystal field Wavefunction
1	0.00	0.00	17.82	-	0.01	49.1%|±6>+49.1%|∓6>
2	0.01	0.01				
3	232.38	334.07	14.18	2.94	0.41	46.1%|+5>+46.1%|−5>
4	232.79	334.66				46.2%|−5>+46.2%|+5>
5	453.14	651.43	10.60	8.96	6.50	38.9%|+4>+38.9%|−4>
6	459.64	660.78				40.9%|−4>+40.9%|+4>
7	633.90	911.29	7.33	18.04	45.62	25.8%|+3>+13.9%|0>+25.8%|−3>
8	679.52	976.88				36.2%|−3>+36.2%|+3>
9	772.15	1110.04	5.50	26.27	128.13	14.7%|+2>+16.9%|+1>+19.5%|0>+16.9%|−1>+14.7%|−2>
10	900.28	1294.24				34.9%|−2>+34.9%|+2>
11	927.18	1332.91	4.32	29.23	167.22	19.0%|+3>+15.6%|+1>+28.1%|0>+15.6%|−1>+19.0%|−3>
12	1094.40	1573.31				35.6%|−1>+35.6%|+1>
13	1102.07	1584.34	-	-	-	22.9%|+2>+38.0%|0>+22.9%|−2>

Only components with > 10% contribution are given, rounded to the nearest percent.

Table S15 CASSCF calculated electronic states for **2-Tb**'s fragment 1.

Levels	*Ab initio* Energy (cm $^{-1}$)	*Ab initio* Energy (K)	g_z	g_z Angle (°)	Δ_{tun} (cm^{-1})	Crystal field Wavefunction
1	0.00	0.00	17.94	-	0.02	49.7%|±6>+49.7%|∓6>
2	0.02	0.02				
3	149.58	215.04	15.90	42.66	1.42	28.0%|+5>+28.0%|−5>
4	151.00	217.08				28.4%|−5>+28.4%|+5>
5	203.74	292.89	12.21	42.66	7.32	17.0%|+5>+10.1%|+2>+16.0%|+1>+16.0%|−1>+10.1%|−2>+17.0%|−5>
6	211.05	303.41				18.2%|−5>+12.0%|−2>+13.2%|0>+12.0%|+2>+18.2%|+5>
7	304.05	437.10	9.05	41.83	21.04	23.4%|+4>+37.1%|0>+23.4%|−4>
8	325.09	467.35				20.8%|−4>+22.1%|−1>+22.1%|+1>+20.8%|+4>
9	425.96	612.35	5.44	37.13	9.52	15.2%|+4>+12.1%|+3>+14.2%|+2>+14.2%|−2>+12.1%|−3>+15.2%|−4>
10	435.47	626.04				15.7%|−4>+10.6%|−1>+22.7%|0>+10.6%|+1>+15.7%|+4>
11	501.88	721.50	3.01	36.96	102.34	26.0%|+3>+15.0%|+2>+15.0%|−2>+26.0%|−3>
12	604.22	868.63				13.4%|−3>+27.1%|−1>+27.1%|+1>+13.4%|+3>
13	618.06	888.52	-	-	-	27.1%|+2>+21.9%|0>+27.1%|−2>

Only components with > 10% contribution are given, rounded to the nearest percent.

Table S16 CASSCF calculated electronic states for **2-Tb**’s fragment 2.

Levels	*Ab initio* Energy (cm $^{-1}$)	*Ab initio* Energy (K)	g_z	g_z Angle (°)	Δ_{tun} (cm^{-1})	Crystal field Wavefunction
1	0.00	0.00	17.93	-	0.02	49.6%|±6>+49.6%|∓6>
2	0.02	0.03				
3	155.02	222.85	15.42	31.68	1.00	36.8%|+5>+36.8%|−5>
4	156.02	224.29				37.1%|−5>+37.1%|+5>
5	243.80	350.48	12.24	37.63	4.81	11.5%|+5>+10.0%|+3>+11.2%|+2>+11.2%|−2>+10.0%|−3>+11.5%|−5>
6	248.60	357.39				12.2%|−5>+11.2%|−3>+11.7%|−2>+11.7%|+2>+11.2%|+3>+12.2%|+5>
7	362.69	521.41	9.09	36.07	16.38	26.0%|+4>+23.3%|0>+26.0%|−4>
8	379.07	544.95				25.7%|−4>+18.3%|−1>+18.3%|+1>+25.7%|+4>
9	487.18	700.37	5.73	35.79	22.35	16.3%|+3>+10.1%|+1>+25.3%|0>+10.1%|−1>+16.3%|−3>
10	509.53	732.51				20.8%|−3>+21.2%|0>+20.8%|+3>
11	586.11	842.59	2.85	37.27	50.89	15.8%|+3>+20.5%|+2>+10.9%|+1>+10.9%|−1>+20.5%|−2>+15.8%|−3>
12	637.00	915.75				12.1%|−3>+21.4%|−1>+12.1%|0>+21.4%|+1>+12.1%|+3>
13	661.78	951.37	-	-	-	28.9%|+2>+11.9%|+1>+11.9%|−1>+28.9%|−2>

Only components with > 10% contribution are given, rounded to the nearest percent.

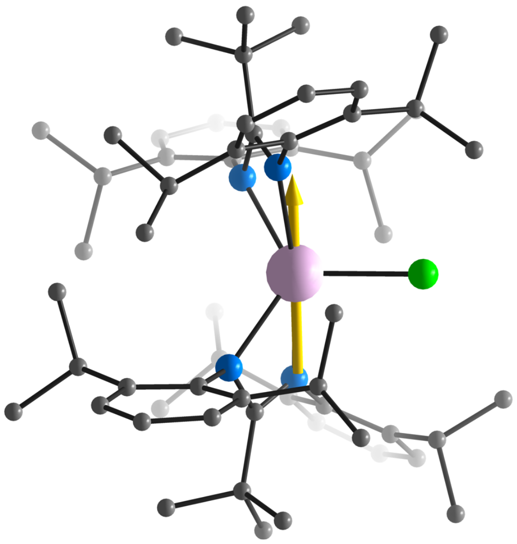

Figure S83 The principal magnetic axis (yellow arrows) of the ground doublets of Dy(III) ion in **1-Dy-Cl**. Color codes: Dy, lavender; N, blue; Cl, green; C, gray. For clarity, all hydrogen atoms are omitted.

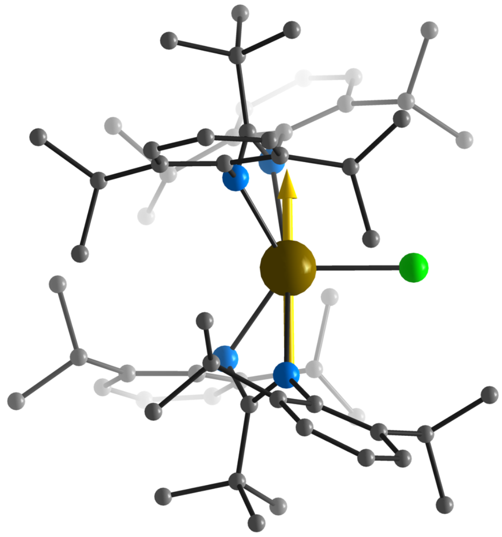

Figure S84 The principal magnetic axis (yellow arrows) of the ground doublets of Tb(III) ion in **1-Tb-Cl**. Color codes: Tb, brown; N, blue; Cl, green; C, gray. For clarity, all hydrogen atoms are omitted.

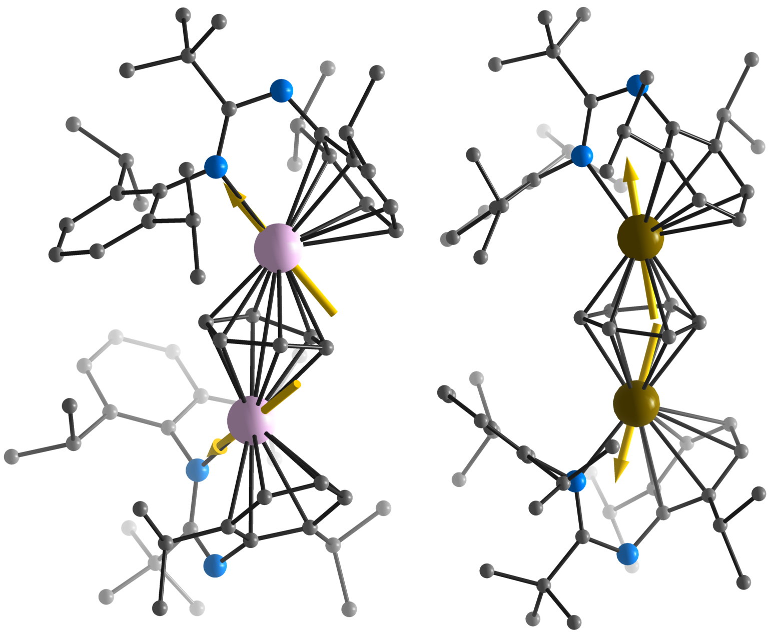

Figure S85 The principal magnetic axes (yellow arrows) of the ground doublets of Dy(III) ions in **2-Dy** (left) and Tb(III) ions in **2-Tb** (right). Color codes: Dy, lavender; Tb, brown; N, blue; C, gray. For clarity, all hydrogen atoms are omitted.

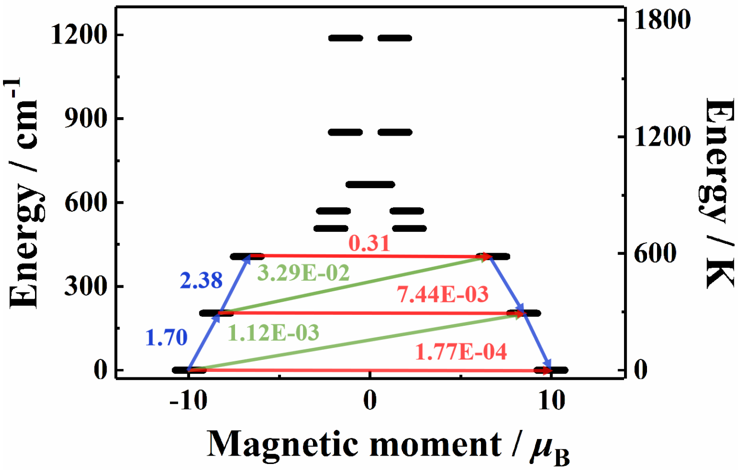

Figure S86 *Ab initio* calculated possible magnetic relaxation path diagram for **1-Dy-Cl**. Eight Kramers' doublets (KDs) are described as the black bars. The horizontal red arrows show the QTM/TA-QTM process and the non-horizontal arrows represent the spin-phonon transition paths. The numbers beside the arrows are averaged transition moments $((|\mu_X|+|\mu_Y|+|\mu_Z|)/3)$ in μ_B between the connecting pairs.

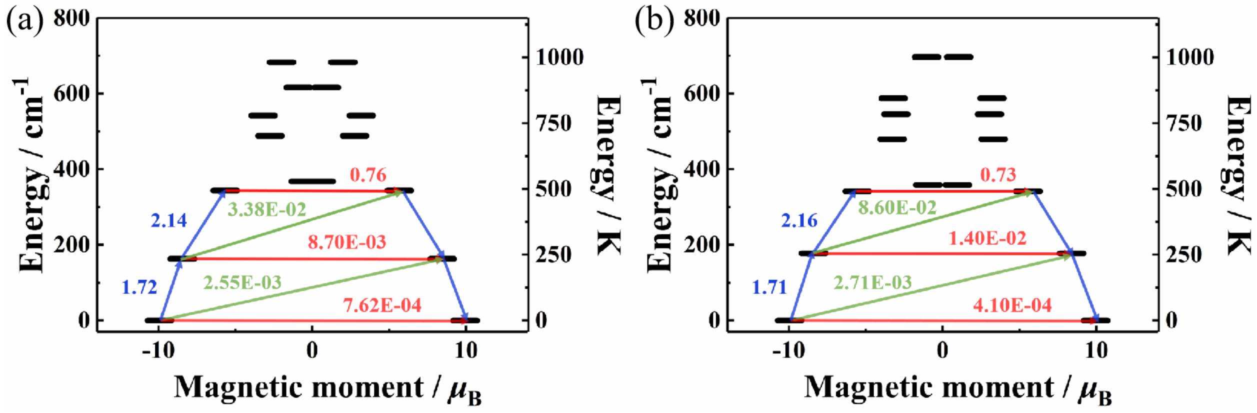

Figure S87 *Ab initio* calculated possible magnetic relaxation path diagrams for **2-Dy**'s fragment 1(a) and 2(b). Eight Kramers' doublets (KDs) are described as the black bars. The horizontal red arrows show the QTM/TA-QTM process and the non-horizontal arrows represent the spin-phonon transition paths. The numbers beside the arrows are averaged transition moments $((|\mu_X|+|\mu_Y|+|\mu_Z|)/3)$ in μ_B between the connecting pairs.

Table S17 *Ab initio* calculated crystal field parameters for Dy(III) ion in **1-Dy-Cl**.

**Crystal Field Parameter B_k^q**		**Value / cm^{-1}**
k	*q*	
2	-2	4.8062519287153E-03
2	-1	1.0770973598370E-03
2	0	-5.1815616481850E+00
2	1	-1.0850722355739E+00
2	2	4.0718366549000E+00
4	-4	-9.8828553622290E-06
4	-3	-1.4657697470966E-05
4	-2	1.9224393286969E-05
4	-1	-1.5605589604050E-05
4	0	3.7142537817825E-05
4	1	4.2108258086722E-02
4	2	-2.1962234608458E-02
4	3	4.8966583501305E-02
4	4	-4.5126456029967E-03
6	-6	-1.1551147425670E-07
6	-5	-4.0664013340428E-08
6	-4	-1.3734824589477E-07
6	-3	-3.8743205636333E-07
6	-2	-3.5746630508274E-07
6	-1	1.1588107982147E-07
6	0	-3.6632757284029E-06
6	1	-6.6129788214455E-04
6	2	2.4739303166539E-04
6	3	-1.8652723523686E-04
6	4	-2.0071418112954E-04
6	5	4.0281081713599E-05
6	6	-3.2876231998927E-05

Table S18 *Ab initio* calculated crystal field parameters for Tb(III) ion in **1-Tb-Cl**.

Crystal Field Parameter B_k^q		Value / cm^{-1}
k	*q*	
2	-2	4.1176619676447E+00
2	-1	-9.2493023810515E-01
2	0	-8.7193831194257E+00
2	1	-2.7937375751905E+00
2	2	5.4725836023040E+00
4	-4	2.2514596725174E-02
4	-3	1.1713575837699E-01
4	-2	2.1890942968479E-02
4	-1	1.9811029785163E-02
4	0	-4.3479897033290E-05
4	1	5.9612865257264E-02
4	2	2.9138308616457E-02
4	3	8.0519499535113E-02
4	4	6.2115314438533E-03
6	-6	1.0530071312060E-04
6	-5	7.5412289964897E-04
6	-4	1.5201667810707E-04
6	-3	-7.4683048494334E-04
6	-2	-3.7378518399250E-04
6	-1	-3.1053811185988E-04
6	0	7.2438155146542E-05
6	1	-9.3620505068346E-04
6	2	-5.7467259758488E-04
6	3	-5.1262452051438E-04
6	4	4.8251515230317E-05
6	5	-4.3560203550689E-05
6	6	-4.2305731399812E-05

Table S19 *Ab initio* calculated crystal field parameters for Dy(III) ion for fragment 1 of **2-Dy**.

**Crystal Field Parameter B_k^q**		**Value / cm^{-1}**
k	*q*	
2	-2	-1.0129846971269E+00
2	-1	1.0103608202064E+00
2	0	-3.0408722767305E+00
2	1	2.1145796051273E+00
2	2	-4.5019989312793E-01
4	-4	3.2728757180396E-03
4	-3	-9.5889429917983E-02
4	-2	1.7412090231467E-02
4	-1	-6.1111035935500E-04
4	0	-6.8940908735961E-03
4	1	1.5538748558562E-03
4	2	1.0274807858115E-02
4	3	-8.5152541723204E-03
4	4	-2.0168276842555E-03
6	-6	1.8910734463029E-04
6	-5	6.8767830209622E-05
6	-4	-1.0125984003195E-04
6	-3	2.6443222255011E-04
6	-2	-1.1084746117011E-04
6	-1	-1.2168923822842E-04
6	0	4.0126886562807E-05
6	1	-2.6311317897230E-04
6	2	4.0108343350265E-05
6	3	-1.4721399570115E-04
6	4	-8.2689056405641E-05
6	5	-8.3360085918372E-05
6	6	-6.5736561256619E-06

Table S20 *Ab initio* calculated crystal field parameters for Dy(III) ion in fragment 2 of **2-Dy**.

**Crystal Field Parameter** B_k^q *k*	*q*	**Value / cm**$^{-1}$
2	-2	-5.7176313043384E-01
2	-1	-1.8630710639546E+00
2	0	-3.0022273905238E+00
2	1	-8.3120039620588E-01
2	2	1.1323556607792E+00
4	-4	-1.1211875416156E-02
4	-3	-5.3114098131481E-02
4	-2	1.8309698879165E-02
4	-1	-2.8925148597594E-03
4	0	-6.3436903880737E-03
4	1	-7.0305261980547E-03
4	2	-1.7001335138726E-02
4	3	-6.3405470446651E-02
4	4	-2.9312508856753E-03
6	-6	-1.9560885656143E-04
6	-5	3.9678094131367E-05
6	-4	-3.5701419425345E-05
6	-3	2.1147443184475E-04
6	-2	-5.2166406587441E-05
6	-1	2.5344371611225E-04
6	0	2.6405498711325E-05
6	1	2.2038913357972E-04
6	2	8.6294901627854E-05
6	3	3.0557903016131E-04
6	4	1.3342690481019E-05
6	5	-1.5676101619977E-04
6	6	-1.0841395993717E-04

Table S21 *Ab initio* calculated crystal field parameters for Tb(III) ion in fragment 1 of **2-Tb**.

**Crystal Field Parameter B_k^q**		**Value / cm^{-1}**
k	*q*	
2	-2	-3.3765390509573E+00
2	-1	-1.0517486969703E+00
2	0	-3.8457392817585E+00
2	1	-6.4617353638220E+00
2	2	-2.0041683317405E+00
4	-4	3.1921614725201E-02
4	-3	7.5010676301514E-04
4	-2	1.1893808762830E-02
4	-1	1.2805923507791E-02
4	0	-9.6099545043993E-03
4	1	8.7198422374446E-02
4	2	2.1223507337922E-03
4	3	9.9832544596242E-03
4	4	-1.1543509972576E-02
6	-6	-3.6709104143094E-05
6	-5	-5.1724021629657E-04
6	-4	3.6863400069862E-05
6	-3	-2.8187015215294E-05
6	-2	-1.2030966579246E-04
6	-1	5.4834789789215E-05
6	0	7.3254850848098E-05
6	1	-4.2258321016795E-04
6	2	-7.4689412612793E-05
6	3	-2.4820359510699E-05
6	4	7.2745677357332E-05
6	5	7.6217129840000E-05
6	6	2.3075844923612E-05

Table S22 *Ab initio* calculated crystal field parameters for Tb(III) ion in fragment 2 of **2-Tb**.

Crystal Field Parameter B_k^q		Value / cm^{-1}
k	*q*	
2	-2	-9.0204134786892E-01
2	-1	7.0762483494893E+00
2	0	-4.7595593947147E+00
2	1	-6.5555170669578E-02
2	2	2.9317706081132E+00
4	-4	-1.9439642877546E-02
4	-3	7.1267237834252E-03
4	-2	9.7525171099289E-03
4	-1	-9.9362707399427E-02
4	0	-5.4520585925333E-03
4	1	1.7551612051869E-03
4	2	2.8663925139866E-03
4	3	1.2980858989694E-03
4	4	7.1791929336751E-03
6	-6	-3.8123367700232E-05
6	-5	8.4254471112075E-04
6	-4	3.7589990292817E-05
6	-3	1.3277379293134E-04
6	-2	-1.2981363208156E-04
6	-1	6.2924585762199E-04
6	0	6.2524343682868E-05
6	1	-5.1989687522581E-05
6	2	4.2751998240666E-05
6	3	6.2192941672031E-05
6	4	1.1114705753784E-04
6	5	1.4621269486685E-04
6	6	-5.4594227415317E-05

Table S23 Average transition magnetic moment elements between the states in **1-Dy-Cl**, given in μ_B.

<table>
<tr><th>Matrix Element
< Mult. i | Mult. j ></th><th>Average Value</th></tr>
<tr><td>< 1.1+ | 1.1- ></td><td>1.77E-04</td></tr>
<tr><td>< 2.1+ | 2.1- ></td><td>7.44E-03</td></tr>
<tr><td>< 3.1+ | 3.1- ></td><td>3.13E-01</td></tr>
<tr><td>< 4.1+ | 4.1- ></td><td>2.60E+00</td></tr>
<tr><td>< 5.1+ | 5.1- ></td><td>1.23E+00</td></tr>
<tr><td>< 6.1+ | 6.1- ></td><td>2.27E+00</td></tr>
<tr><td>< 7.1+ | 7.1- ></td><td>3.68E-01</td></tr>
<tr><td>< 8.1+ | 8.1- ></td><td>2.35E-02</td></tr>
<tr><td>< 1.1+ | 2.1+ ></td><td>1.70E+00</td></tr>
<tr><td>< 1.1+ | 2.1- ></td><td>1.12E-03</td></tr>
<tr><td>< 2.1+ | 3.1+ ></td><td>2.38E+00</td></tr>
<tr><td>< 2.1+ | 3.1- ></td><td>3.29E-02</td></tr>
<tr><td>< 3.1+ | 4.1+ ></td><td>2.36E+00</td></tr>
<tr><td>< 3.1+ | 4.1- ></td><td>8.51E-01</td></tr>
<tr><td>< 4.1+ | 5.1+ ></td><td>2.16E+00</td></tr>
<tr><td>< 4.1+ | 5.1- ></td><td>2.24E+00</td></tr>
<tr><td>< 5.1+ | 6.1+ ></td><td>1.93E+00</td></tr>
<tr><td>< 5.1+ | 6.1- ></td><td>2.10E+00</td></tr>
<tr><td>< 6.1+ | 7.1+ ></td><td>1.92E+00</td></tr>
<tr><td>< 6.1+ | 7.1- ></td><td>1.79E+00</td></tr>
<tr><td>< 7.1+ | 8.1+ ></td><td>1.92E+00</td></tr>
<tr><td>< 7.1+ | 8.1- ></td><td>1.13E-01</td></tr>
<tr><td>< 1.1+ | 3.1+ ></td><td>5.58E-01</td></tr>
<tr><td>< 1.1+ | 3.1- ></td><td>5.36E-03</td></tr>
<tr><td>< 2.1+ | 4.1+ ></td><td>2.29E-01</td></tr>
<tr><td>< 2.1+ | 4.1- ></td><td>3.05E-01</td></tr>
<tr><td>< 3.1+ | 5.1+ ></td><td>9.75E-01</td></tr>
<tr><td>< 3.1+ | 5.1- ></td><td>1.22E+00</td></tr>
<tr><td>< 4.1+ | 6.1+ ></td><td>9.37E-01</td></tr>
<tr><td>< 4.1+ | 6.1- ></td><td>4.08E-01</td></tr>
<tr><td>< 5.1+ | 7.1+ ></td><td>2.26E-01</td></tr>
<tr><td>< 5.1+ | 7.1- ></td><td>6.43E-01</td></tr>
<tr><td>< 6.1+ | 8.1+ ></td><td>4.18E-01</td></tr>
<tr><td>< 6.1+ | 8.1- ></td><td>3.18E-01</td></tr>
<tr><td>< 1.1+ | 4.1+ ></td><td>8.21E-02</td></tr>
<tr><td>< 1.1+ | 4.1- ></td><td>1.03E-01</td></tr>
<tr><td>< 2.1+ | 5.1+ ></td><td>1.52E-01</td></tr>
<tr><td>< 2.1+ | 5.1- ></td><td>2.69E-01</td></tr>
<tr><td>< 3.1+ | 6.1+ ></td><td>9.53E-01</td></tr>
<tr><td>< 3.1+ | 6.1- ></td><td>4.52E-01</td></tr>
<tr><td>< 4.1+ | 7.1+ ></td><td>3.23E-01</td></tr>
<tr><td>< 4.1+ | 7.1- ></td><td>4.02E-01</td></tr>
<tr><td>< 5.1+ | 8.1+ ></td><td>8.24E-02</td></tr>
<tr><td>< 5.1+ | 8.1- ></td><td>2.98E-01</td></tr>
<tr><td>< 1.1+ | 5.1+ ></td><td>8.32E-02</td></tr>
<tr><td>< 1.1+ | 5.1- ></td><td>9.53E-02</td></tr>
<tr><td>< 2.1+ | 6.1+ ></td><td>1.41E-01</td></tr>
<tr><td>< 2.1+ | 6.1- ></td><td>1.65E-01</td></tr>
<tr><td>< 3.1+ | 7.1+ ></td><td>5.44E-01</td></tr>
<tr><td>< 3.1+ | 7.1- ></td><td>1.53E-01</td></tr>
<tr><td>< 4.1+ | 8.1+ ></td><td>1.09E-01</td></tr>
<tr><td>< 4.1+ | 8.1- ></td><td>1.11E-01</td></tr>
<tr><td>< 1.1+ | 6.1+ ></td><td>5.68E-02</td></tr>
<tr><td>< 1.1+ | 6.1- ></td><td>8.23E-02</td></tr>
<tr><td>< 2.1+ | 7.1+ ></td><td>1.72E-01</td></tr>
<tr><td>< 2.1+ | 7.1- ></td><td>3.38E-02</td></tr>
<tr><td>< 3.1+ | 8.1+ ></td><td>2.16E-01</td></tr>
<tr><td>< 3.1+ | 8.1- ></td><td>3.67E-02</td></tr>
<tr><td>< 1.1+ | 7.1+ ></td><td>2.73E-02</td></tr>
<tr><td>< 1.1+ | 7.1- ></td><td>1.20E-02</td></tr>
<tr><td>< 2.1+ | 8.1+ ></td><td>9.11E-02</td></tr>
<tr><td>< 2.1+ | 8.1- ></td><td>7.44E-03</td></tr>
<tr><td>< 1.1+ | 8.1+ ></td><td>2.10E-02</td></tr>
<tr><td>< 1.1+ | 8.1- ></td><td>2.53E-03</td></tr>
</table>

Table S24 Average transition magnetic moment elements between the states in fragment 1 of **2-Dy**, given in μ_B.

Matrix Element<br>< Mult. *i* | Mult. *j* >	Average Value
< 1.1+ | 1.1- >	7.62E-04
< 2.1+ | 2.1- >	8.70E-03
< 3.1+ | 3.1- >	7.64E-01
< 4.1+ | 4.1- >	3.45E+00
< 5.1+ | 5.1- >	1.87E+00
< 6.1+ | 6.1- >	1.79E-01
< 7.1+ | 7.1- >	2.29E+00
< 8.1+ | 8.1- >	3.38E+00
< 1.1+ | 2.1+ >	1.72E+00
< 1.1+ | 2.1- >	2.55E-03
< 2.1+ | 3.1+ >	2.14E+00
< 2.1+ | 3.1- >	3.38E-02
< 3.1+ | 4.1+ >	1.06E+00
< 3.1+ | 4.1- >	2.23E+00
< 4.1+ | 5.1+ >	2.02E+00
< 4.1+ | 5.1- >	1.52E+00
< 5.1+ | 6.1+ >	1.71E+00
< 5.1+ | 6.1- >	1.27E+00
< 6.1+ | 7.1+ >	1.11E+00
< 6.1+ | 7.1- >	8.29E-01
< 7.1+ | 8.1+ >	2.69E+00
< 7.1+ | 8.1- >	2.45E+00
< 1.1+ | 3.1+ >	5.35E-01
< 1.1+ | 3.1- >	1.22E-02
< 2.1+ | 4.1+ >	9.09E-01
< 2.1+ | 4.1- >	5.13E-01
< 3.1+ | 5.1+ >	1.93E+00
< 3.1+ | 5.1- >	3.50E-01
< 4.1+ | 6.1+ >	8.92E-01
< 4.1+ | 6.1- >	1.18E+00
< 5.1+ | 7.1+ >	2.85E+00
< 5.1+ | 7.1- >	1.09E+00
< 6.1+ | 8.1+ >	4.73E-01
< 6.1+ | 8.1- >	5.93E-01
< 1.1+ | 4.1+ >	2.28E-01
< 1.1+ | 4.1- >	1.11E-01
< 2.1+ | 5.1+ >	5.40E-01
< 2.1+ | 5.1- >	1.98E-01
< 3.1+ | 6.1+ >	1.41E+00
< 3.1+ | 6.1- >	3.82E-01
< 4.1+ | 7.1+ >	5.12E-01
< 4.1+ | 7.1- >	3.86E-01
< 5.1+ | 8.1+ >	4.26E-01
< 5.1+ | 8.1- >	5.28E-01
< 1.1+ | 5.1+ >	1.35E-01
< 1.1+ | 5.1- >	5.32E-02
< 2.1+ | 6.1+ >	2.64E-01
< 2.1+ | 6.1- >	8.40E-02
< 3.1+ | 7.1+ >	8.99E-01
< 3.1+ | 7.1- >	2.51E-01
< 4.1+ | 8.1+ >	2.17E-01
< 4.1+ | 8.1- >	3.34E-01
< 1.1+ | 6.1+ >	8.20E-02
< 1.1+ | 6.1- >	3.11E-02
< 2.1+ | 7.1+ >	2.36E-01
< 2.1+ | 7.1- >	1.51E-01
< 3.1+ | 8.1+ >	2.16E-01
< 3.1+ | 8.1- >	2.51E-01
< 1.1+ | 7.1+ >	8.92E-02
< 1.1+ | 7.1- >	6.24E-02
< 2.1+ | 8.1+ >	1.40E-01
< 2.1+ | 8.1- >	1.09E-01
< 1.1+ | 8.1+ >	7.28E-02
< 1.1+ | 8.1- >	2.72E-02

Table S25 Average transition magnetic moment elements between the states in fragment 2 of **2-Dy**, given in μ_B.

<table>
<tr><th>Matrix Element
< Mult. i | Mult. j ></th><th>Average Value</th></tr>
<tr><td>< 1.1+ | 1.1- ></td><td>4.10E-04</td></tr>
<tr><td>< 2.1+ | 2.1- ></td><td>1.40E-02</td></tr>
<tr><td>< 3.1+ | 3.1- ></td><td>7.34E-01</td></tr>
<tr><td>< 4.1+ | 4.1- ></td><td>2.83E+00</td></tr>
<tr><td>< 5.1+ | 5.1- ></td><td>1.12E+00</td></tr>
<tr><td>< 6.1+ | 6.1- ></td><td>6.10E-01</td></tr>
<tr><td>< 7.1+ | 7.1- ></td><td>1.45E+00</td></tr>
<tr><td>< 8.1+ | 8.1- ></td><td>2.39E+00</td></tr>
<tr><td>< 1.1+ | 2.1+ ></td><td>1.71E+00</td></tr>
<tr><td>< 1.1+ | 2.1- ></td><td>2.71E-03</td></tr>
<tr><td>< 2.1+ | 3.1+ ></td><td>2.16E+00</td></tr>
<tr><td>< 2.1+ | 3.1- ></td><td>8.60E-02</td></tr>
<tr><td>< 3.1+ | 4.1+ ></td><td>1.41E+00</td></tr>
<tr><td>< 3.1+ | 4.1- ></td><td>1.92E+00</td></tr>
<tr><td>< 4.1+ | 5.1+ ></td><td>1.59E+00</td></tr>
<tr><td>< 4.1+ | 5.1- ></td><td>1.59E+00</td></tr>
<tr><td>< 5.1+ | 6.1+ ></td><td>1.86E+00</td></tr>
<tr><td>< 5.1+ | 6.1- ></td><td>9.70E-01</td></tr>
<tr><td>< 6.1+ | 7.1+ ></td><td>9.82E-01</td></tr>
<tr><td>< 6.1+ | 7.1- ></td><td>9.48E-01</td></tr>
<tr><td>< 7.1+ | 8.1+ ></td><td>1.87E+00</td></tr>
<tr><td>< 7.1+ | 8.1- ></td><td>1.68E+00</td></tr>
<tr><td>< 1.1+ | 3.1+ ></td><td>5.33E-01</td></tr>
<tr><td>< 1.1+ | 3.1- ></td><td>2.46E-02</td></tr>
<tr><td>< 2.1+ | 4.1+ ></td><td>8.65E-01</td></tr>
<tr><td>< 2.1+ | 4.1- ></td><td>6.56E-01</td></tr>
<tr><td>< 3.1+ | 5.1+ ></td><td>2.38E+00</td></tr>
<tr><td>< 3.1+ | 5.1- ></td><td>3.65E-01</td></tr>
<tr><td>< 4.1+ | 6.1+ ></td><td>7.15E-01</td></tr>
<tr><td>< 4.1+ | 6.1- ></td><td>1.30E+00</td></tr>
<tr><td>< 5.1+ | 7.1+ ></td><td>2.62E+00</td></tr>
<tr><td>< 5.1+ | 7.1- ></td><td>2.00E+00</td></tr>
<tr><td>< 6.1+ | 8.1+ ></td><td>4.54E-01</td></tr>
<tr><td>< 6.1+ | 8.1- ></td><td>4.81E-01</td></tr>
<tr><td>< 1.1+ | 4.1+ ></td><td>2.21E-01</td></tr>
<tr><td>< 1.1+ | 4.1- ></td><td>1.38E-01</td></tr>
<tr><td>< 2.1+ | 5.1+ ></td><td>5.59E-01</td></tr>
<tr><td>< 2.1+ | 5.1- ></td><td>1.19E-01</td></tr>
<tr><td>< 3.1+ | 6.1+ ></td><td>1.06E+00</td></tr>
<tr><td>< 3.1+ | 6.1- ></td><td>2.49E-01</td></tr>
<tr><td>< 4.1+ | 7.1+ ></td><td>5.82E-01</td></tr>
<tr><td>< 4.1+ | 7.1- ></td><td>4.96E-01</td></tr>
<tr><td>< 5.1+ | 8.1+ ></td><td>6.87E-01</td></tr>
<tr><td>< 5.1+ | 8.1- ></td><td>7.76E-01</td></tr>
<tr><td>< 1.1+ | 5.1+ ></td><td>1.94E-01</td></tr>
<tr><td>< 1.1+ | 5.1- ></td><td>3.94E-02</td></tr>
<tr><td>< 2.1+ | 6.1+ ></td><td>3.11E-01</td></tr>
<tr><td>< 2.1+ | 6.1- ></td><td>1.79E-02</td></tr>
<tr><td>< 3.1+ | 7.1+ ></td><td>7.34E-01</td></tr>
<tr><td>< 3.1+ | 7.1- ></td><td>4.81E-01</td></tr>
<tr><td>< 4.1+ | 8.1+ ></td><td>2.36E-01</td></tr>
<tr><td>< 4.1+ | 8.1- ></td><td>1.64E-01</td></tr>
<tr><td>< 1.1+ | 6.1+ ></td><td>3.73E-02</td></tr>
<tr><td>< 1.1+ | 6.1- ></td><td>9.65E-03</td></tr>
<tr><td>< 2.1+ | 7.1+ ></td><td>1.94E-01</td></tr>
<tr><td>< 2.1+ | 7.1- ></td><td>1.15E-01</td></tr>
<tr><td>< 3.1+ | 8.1+ ></td><td>2.74E-01</td></tr>
<tr><td>< 3.1+ | 8.1- ></td><td>1.46E-01</td></tr>
<tr><td>< 1.1+ | 7.1+ ></td><td>9.18E-02</td></tr>
<tr><td>< 1.1+ | 7.1- ></td><td>3.62E-02</td></tr>
<tr><td>< 2.1+ | 8.1+ ></td><td>1.15E-01</td></tr>
<tr><td>< 2.1+ | 8.1- ></td><td>8.41E-02</td></tr>
<tr><td>< 1.1+ | 8.1+ ></td><td>5.39E-02</td></tr>
<tr><td>< 1.1+ | 8.1- ></td><td>2.46E-02</td></tr>
</table>

Table S26 Calculated energies of spin-orbit states (cm^{-1}), the corresponding tunneling splitting's (cm^{-1}) and g_z values of the low-lying exchange doublets of **2-Dy**.

E	Δ_{tun}	g_z
0.000	5.73×10^{-6}	26.403
0.000		
1.038	5.41×10^{-6}	29.269
1.038		

Table S27 Calculated energies of spin-orbit states (cm^{-1}), the corresponding tunneling splittings (cm^{-1}) and g_z values of the low-lying exchange doublets of **2-Tb**.

E	Δ_{tun}	g_z
0.000	2.96×10^{-5}	7.528
0.000		
13.644	2.95×10^{-5}	34.999
13.644		

Table S28 DFT calculated energies of the high spin (HS) and broken symmetry (BS) states for model complex **2-Gd***.

Spin State	Energy / Eh	Spin Value $<S^2>$	$J_{ex}^{Gd\text{-}Gd}$ / cm^{-1}	$J_{ex}^{Dy\text{-}Dy}$ / cm^{-1}	$J_{ex}^{Tb\text{-}Tb}$ / cm^{-1}
HS	24893.268369	56.0549	-2.56	-5.02	-3.48
BS	24893.268940	7.0988			

Table S29 DFT calculated energies of the high spin (HS) and broken symmetry (BS) states for model complex **2-Gd***.

	Spin State	Spin population on Gd1	Spin population on Gd2
**2-Gd***	HS	7.173499	7.173575
	BS	7.302390	-7.302311

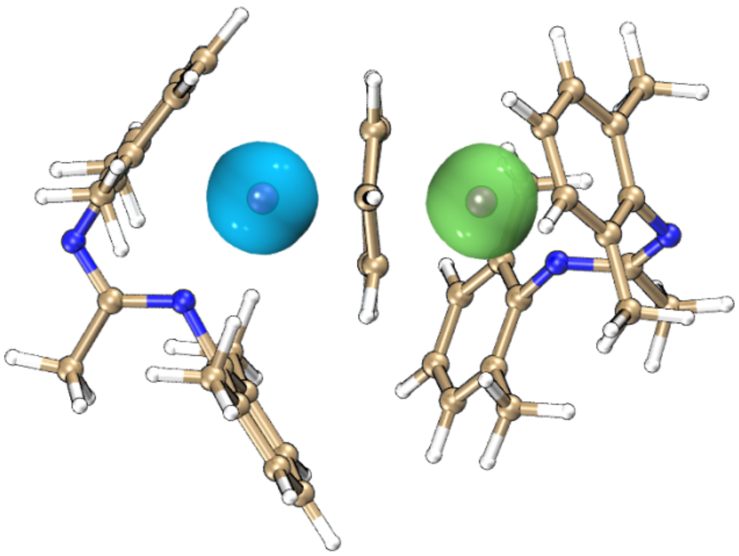

Figure S88 DFT calculated spin density distributions (isovalue = 0.01 a.u.) of the BS state for model complex **2-Gd***. Color codes: Gd, purple; N, blue; C, tan; H, white.

Table S30 Natural population analysis (NPA) for **2-Y^{opt}**. Note: C atoms marked in blue and red correspond to the ones on the central $C_6H_6^{4-}$ bridge and phenyl of terminal Piso ligand.

Atom	Natural Charge	Core	Valence	Rydberg	Total
Y	1.23996	35.97734	1.70044	0.08227	37.76004
Y	1.24124	35.97728	1.69991	0.08157	37.75876
N	-0.74058	1.99917	5.71923	0.02218	7.74058
**C**	**-0.54279**	1.99834	4.51797	0.02648	6.54279
**C**	**-0.53339**	1.99832	4.50773	0.02734	6.53339
**C**	**-0.25813**	1.99887	4.23977	0.01949	6.25813
**C**	**-0.5303**	1.99847	4.50504	0.02679	6.5303
**C**	**-0.56701**	1.99817	4.53994	0.02889	6.56701
**C**	**-0.51932**	1.99847	4.49427	0.02658	6.51932
**C**	**-0.28275**	1.99895	4.26257	0.02123	6.28275
**C**	**-0.04233**	1.99883	4.0197	0.02381	6.04233
**C**	**-0.53008**	1.99846	4.50386	0.02777	6.53008
**C**	**0.15637**	1.99855	3.81731	0.02777	5.84363
**C**	**-0.26734**	1.99886	4.24869	0.01979	6.26734
**C**	**-0.04548**	1.9988	4.02286	0.02382	6.04548
N	-0.74348	1.99917	5.72225	0.02207	7.74348
**C**	**0.15181**	1.99854	3.82129	0.02837	5.84819
**C**	**-0.04626**	1.99879	4.02305	0.02442	6.04626
**C**	**-0.26779**	1.99885	4.24865	0.02029	6.26779
**C**	**-0.04314**	1.99882	4.01986	0.02445	6.04314
**C**	**-0.25495**	1.99887	4.2368	0.01929	6.25495
**C**	**-0.27882**	1.99895	4.25943	0.02043	6.27882
C	0.46898	1.99916	3.50616	0.0257	5.53102
**C**	**0.15641**	1.99881	3.82196	0.02282	5.84359
H	0.30279	0	0.69479	0.00242	0.69721
H	0.30552	0	0.69213	0.00235	0.69448
H	0.268	0	0.72972	0.00228	0.732
H	0.29036	0	0.70732	0.00232	0.70964
H	0.31276	0	0.68483	0.00242	0.68724
H	0.29122	0	0.70639	0.00239	0.70878
H	0.27565	0	0.72211	0.00224	0.72435
C	-0.73812	1.99954	4.72985	0.00874	6.73812
H	0.28586	0	0.71188	0.00226	0.71414
N	-0.56729	1.99927	5.53439	0.03363	7.56729
H	0.27145	0	0.72627	0.00228	0.72855
C	-0.73791	1.99954	4.72949	0.00888	6.73791
C	0.47191	1.99916	3.50394	0.02499	5.52809
**C**	**0.15664**	1.99883	3.82133	0.0232	5.84336
N	-0.5667	1.99928	5.53359	0.03383	7.5667
C	-0.73736	1.99953	4.72903	0.0088	6.73736
H	0.27041	0	0.72725	0.00234	0.72959
C	-0.73877	1.99954	4.73045	0.00878	6.73877
H	0.26811	0	0.72957	0.00232	0.73189
H	0.27559	0	0.7222	0.00221	0.72441
C	-0.78159	1.99951	4.77298	0.0091	6.78159
**C**	**-0.0339**	1.99897	4.01648	0.01846	6.0339
**C**	**-0.04077**	1.99897	4.02345	0.01835	6.04077
H	0.26939	0	0.72815	0.00246	0.73061
H	0.2706	0	0.72701	0.0024	0.7294
C	-0.78305	1.99952	4.77508	0.00845	6.78305

**C**	**-0.03908**	1.99898	4.02234	0.01776	6.03908
**C**	**-0.0338**	1.99898	4.01678	0.01804	6.0338
H	0.27012	0	0.72762	0.00226	0.72988
H	0.26976	0	0.7279	0.00234	0.73024
**C**	**-0.2477**	1.999	4.23278	0.01592	6.2477
C	-0.73207	1.99953	4.72461	0.00793	6.73207
**C**	**-0.25193**	1.999	4.23715	0.01578	6.25193
C	-0.73137	1.99953	4.72404	0.00779	6.73137
C	-0.73367	1.99955	4.72645	0.00767	6.73367
**C**	**-0.24954**	1.99902	4.23533	0.01519	6.24954
C	-0.73275	1.99953	4.72514	0.00808	6.73275
**C**	**-0.24796**	1.99902	4.23333	0.01561	6.24796
H	0.23996	0	0.75766	0.00238	0.76004
**C**	**-0.25596**	1.99907	4.24117	0.01572	6.25596
H	0.25055	0	0.74694	0.00251	0.74945
H	0.23998	0	0.75768	0.00233	0.76002
H	0.25467	0	0.74283	0.0025	0.74533
H	0.25467	0	0.74281	0.00253	0.74533
**C**	**-0.25553**	1.99908	4.24124	0.01521	6.25553
H	0.23945	0	0.7581	0.00245	0.76055
H	0.24974	0	0.74779	0.00247	0.75026
H	0.23901	0	0.75851	0.00249	0.76099
H	0.24452	0	0.75313	0.00234	0.75548
H	0.24449	0	0.75312	0.00239	0.75551
H	0.25153	0	0.74627	0.0022	0.74847
H	0.25255	0	0.74492	0.00252	0.74745
H	0.25427	0	0.74348	0.00225	0.74573
H	0.26003	0	0.7375	0.00248	0.73997
H	0.27659	0	0.72058	0.00283	0.72341
H	0.24945	0	0.74844	0.00212	0.75055
H	0.25068	0	0.74724	0.00208	0.74932
H	0.2772	0	0.72	0.0028	0.7228
H	0.27329	0	0.72451	0.00219	0.72671
H	0.26034	0	0.7375	0.00216	0.73966
H	0.2619	0	0.73597	0.00213	0.7381
H	0.27646	0	0.72076	0.00279	0.72354
H	0.24924	0	0.74872	0.00204	0.75076
H	0.2507	0	0.74727	0.00203	0.7493
H	0.27707	0	0.72015	0.00278	0.72293
H	0.26016	0	0.73767	0.00217	0.73984
H	0.2611	0	0.73674	0.00216	0.7389
H	0.27337	0	0.72453	0.0021	0.72663
H	0.2552	0	0.74257	0.00223	0.7448
H	0.2584	0	0.73909	0.00251	0.7416
H	0.25166	0	0.7462	0.00214	0.74834
H	0.25163	0	0.74583	0.00253	0.74837
Total	0	163.90878	240.91881	1.1724	406

Table S31 Calculated atoms-in-molecules (AIM) charges based on the basin analysis for **2-Y^{opt}**. Note: C atoms marked in blue and red correspond to the ones on the central $C_6H_6^{4-}$ bridge and phenyl of terminal Piso ligand.

Atom	AIM Charge	Atomic Volume/ Bohr^3	Atom	AIM Charge	Atomic Volume/ Bohr^3
Y	1.809891	109.69	H	0.015273	46.499
Y	1.810138	109.9	C	0.061172	65.135
N	-1.318902	86.637	C	-0.021838	65.578
**C**	**-0.365765**	80.396	C	-0.018374	64.108
**C**	**-0.358442**	79.404	H	0.013159	46.103
C	-0.085135	76.405	H	0.013868	46.218
C	-0.349804	81.383	C	-0.019771	82.363
C	-0.363134	65.853	C	0.078635	64.18
C	-0.347536	81.942	C	-0.023666	81.341
C	-0.0944	72.777	C	0.079123	63.709
C	-0.07859	64.109	C	0.080091	63.722
C	-0.327343	69.984	C	-0.023269	81.491
C	0.3649	50.305	C	0.079101	64.46
C	-0.094957	75.572	C	-0.019492	82.022
C	-0.083635	63.419	H	-0.001253	49.323
N	-1.31531	86.779	C	-0.021608	84.323
C	0.361731	50.335	H	-0.018353	48.365
C	-0.084428	63.379	H	-0.002203	48.511
C	-0.0946	75.966	H	-0.017512	49.818
C	-0.076639	64.057	H	-0.016895	49.313
C	-0.083837	76.109	C	-0.022034	84.173
C	-0.093708	72.815	H	-0.00163	48.502
C	1.123632	41.907	H	-0.018768	47.562
C	0.350435	52.081	H	-0.002551	49.298
H	0.035062	46.974	H	0.003564	48.764
H	0.041079	46.447	H	0.00448	48.785
H	0.03064	47.573	H	-0.022051	48.871
H	0.011088	49.656	H	-0.014546	47.861
H	0.056096	41.655	H	-0.016785	50.714
H	0.013285	49.855	H	-0.010091	48.846
H	0.03787	47.081	H	0.018927	47.377
C	0.08063	65.174	H	-0.018755	48.943
H	0.002772	49.789	H	-0.017453	48.811
N	-1.232282	112.177	H	0.020157	47.247
H	0.034193	47.633	H	0.013672	47.327
C	0.077683	65.26	H	-0.002295	46.815
C	1.122136	41.856	H	-0.001657	47.328
C	0.34883	51.969	H	0.018627	47.397
N	-1.233595	112.056	H	-0.020378	48.943
C	0.078738	65.186	H	-0.018014	48.848
H	0.033062	47.69	H	0.019725	47.34
C	0.078929	65.228	H	-0.003289	47.27
H	0.031522	47.548	H	-0.001622	47.004
H	0.038087	46.942	H	0.013403	47.328
C	0.061841	65.215	H	-0.015866	50.954
C	-0.01765	64.037	H	-0.011191	47.967
C	-0.021758	64.949	H	-0.021174	48.84
H	0.013906	45.661	H	-0.015319	47.596

Table S32 The calculated properties of real space functions at BCPs for **2Y^{opt}** (a.u.).

	Y1-N1	Y1-C1	Y1-C2	Y1-C3	Y1-C4	Y1-C5	Y2-N2	Y2-C1
ρ(r)	0.0539	0.0556	0.0503	0.0522	0.0262	0.0274	0.0543	0.0533
H(r)	-0.0052	-0.0071	-0.0048	-0.0055	0.0004	0.0003	-0.0053	-0.0063
$\nabla^2_{\rho(r)}$	0.1708	0.1748	0.1683	0.1722	0.0879	0.0910	0.1720	0.1662
G(r)	0.0478	0.0508	0.0469	0.0486	0.0215	0.0225	0.0483	0.0479
V(r)	-0.0530	-0.0579	-0.0518	-0.0541	-0.0211	-0.0222	-0.0535	-0.0542
|*V*(r)|/*G*(r)	1.1088	1.1398	1.1045	1.1132	0.9814	0.9867	1.1077	1.1315
	Y2-C2	Y2-C3	Y2-C6	Y2-C7				
ρ(r)	0.0532	0.0527	0.0267	0.0274				
H(r)	-0.0061	-0.0056	0.0004	0.0003				
$\nabla^2_{\rho(r)}$	0.1668	0.1774	0.0892	0.0901				
G(r)	0.0478	0.0500	0.0220	0.0223				
V(r)	-0.0540	-0.0557	-0.0216	-0.0220				
|*V*(r)|/*G*(r)	1.1297	1.1140	0.9818	0.9865				

Note: Poincare-Hopf relationship is satisfied, indicating that all CPs may have been found. C1-C3 represent the carbon atoms on the central $C_6H_6^{4-}$ bridge while the rest of carbon atoms C4-C7 are on the terminal Piso ligand.

8. References

1. W. A. Blackmore, G. K. Gransbury, P. Evans, D. P. Mills and N. F Chilton, *Phys. Chem. Chem. Phys*, 2023, **25**, 16735–16744.
2. G. K. Gransbury, S. C. Corner, J. G. C. Kragskow, P. Evans, H. M. Yeung, W. J. A. Blackmore, G. F. S. Whitehead, I. J. Vitorica-Yrezabal, N. F. Chilton and D. P. Mills, *J. Am. Chem. Soc.,* 2023, **145**, 22814‒22825.
3. R. Zorn, *J. Chem. Phys.* 2002, **116**, 3204–3209.
